# Fungal dye-decolorizing peroxidase diversity: roles in either intra- or extracellular processes

**DOI:** 10.1007/s00253-022-11923-0

**Published:** 2022-04-18

**Authors:** Martino Adamo, Sophie Comtet-Marre, Enrico Büttner, Harald Kellner, Patricia Luis, Laurent Vallon, Rocio Prego, Martin Hofrichter, Mariangela Girlanda, Pierre Peyret, Roland Marmeisse

**Affiliations:** 1 Department of Life Sciences and Systems Biology (DBIOS), Università Degli Studi Di Torino, 25 Viale P.A. Mattioli, 10125 Torino, Italy; 2 Univ Lyon, Université Claude Bernard Lyon 1, CNRS, INRAE, UMR Ecologie Microbienne, VetAgro Sup43 Boulevard du 11 Novembre 1918, 69622 Villeurbanne Cedex, France; 3 Université Clermont Auvergne, INRAE, MEDiS, 63000 Clermont-Ferrand, France; 4 Department of Bio- and Environmental Sciences, International Institute Zittau, Technische Universität Dresden, Zittau, Germany; 5 Institut de Systématique, Evolution, Biodiversité (ISYEB), Muséum National d’Histoire Naturelle, CNRS, Sorbonne Université, EPHE, Université Des Antilles, CP39, 57 rue Cuvier, 75005 Paris, France; 6 Institute for Sustainable Plant Protection (IPSP), National Research Council (CNR), 25 Viale P.A. Mattioli, 10125 Torino, Italy

**Keywords:** DyP-type peroxidase, Lignin degradation, *Fungi*, Sequence capture

## Abstract

**Abstract:**

Fungal dye-decolorizing peroxidases (DyPs) have found applications in the treatment of dye-contaminated industrial wastes or to improve biomass digestibility. Their roles in fungal biology are uncertain, although it has been repeatedly suggested that they could participate in lignin degradation and/or modification. Using a comprehensive set of 162 fully sequenced fungal species, we defined seven distinct fungal DyP clades on basis of a sequence similarity network. Sequences from one of these clades clearly diverged from all others, having on average the lower isoelectric points and hydropathy indices, the highest number of *N*-glycosylation sites, and N-terminal sequence peptides for secretion. Putative proteins from this clade are absent from brown-rot and ectomycorrhizal species that have lost the capability of degrading lignin enzymatically. They are almost exclusively present in white-rot and other saprotrophic *Basidiomycota* that digest lignin enzymatically, thus lending support for a specific role of DyPs from this clade in biochemical lignin modification. Additional nearly full-length fungal DyP genes were isolated from the environment by sequence capture by hybridization; they all belonged to the clade of the presumably secreted DyPs and to another related clade. We suggest focusing our attention on the presumably intracellular DyPs from the other clades, which have not been characterized thus far and could represent enzyme proteins with novel catalytic properties.

**Key points:**

• *A fungal DyP phylogeny delineates seven main sequence clades.*

• *Putative extracellular DyPs form a single clade of Basidiomycota sequences.*

• *Extracellular DyPs are associated to white-rot fungi.*

**Supplementary Information:**

The online version contains supplementary material available at 10.1007/s00253-022-11923-0.

## Introduction

Lignin is a complex plant cell-wall polymer made of different cross-linked phenylpropanoid monomers (Schmidt 2006) that represents about 30% of the plant-derived organic carbon available in the biosphere (Boerjan et al. 2003). Besides providing structural rigidity in living plants, another important function is to protect cell wall polysaccharides from degradation by hydrolytic enzymes secreted by microorganisms (lignin barrier). Control of lignin degradation represents an important field of research either for the use of this polymer as a source of aromatic molecules or simply for its removal from plant biomass to access cell-wall polysaccharides for biorefinery and biofuel production (Duval and Lawoko 2014; Chio et al. 2019).

White-rot (WR) fungi, a specific functional group of saprotrophic *Basidiomycota*, are considered the main group of microorganisms able to perform enzymatic ligninolysis. Phylogenomic studies support the assumption that the WR phenotype is an ancestral character that arose once early in the evolution of the *Basidiomycota* (Floudas et al. 2012). Subsequently, the WR phenotype was lost several times independently during the course of evolution to give rise to different lineages of either brown-rot (BR) saprotrophic or mutualistic ectomycorrhizal (EM) *Basidiomycota* that associate to plant roots (Kohler et al. 2015; Martin et al. 2016; Nagy et al. 2017; Miyauchi et al. 2020). Both BR and EM fungi have lost the capacity of performing enzymatic ligninolysis, although being capable of modifying the lignin structure to some extent using oxidizing mechanisms (Arantes et al. 2012; Rineau et al. 2012).

From a mechanistic point of view, biochemical and genetic studies have demonstrated that several secreted enzymes belonging to the so-called heme-containing, fungal-specific class II peroxidases (E.C. 1.11.1.-) participate directly or indirectly in lignin decomposition (Hammel and Cullen 2008; Salame et al. 2014; Ayuso-Fernández et al. 2018). Among these class II peroxidases, lignin (LiP, E.C. 1.11.1.14) and versatile (VP, E.C. 1.11.1.16) peroxidases interact directly with lignin to cleave non-phenolic ether bonds that link phenylpropanoid monomers. A further class II enzyme, manganese peroxidase (MnP, E.C. 1.11.1.13), but also VPs, oxidize Mn^2+^ to Mn^3+^, which is then chelated with organic acids and cleaves as low-molecular mass and diffusible redox-mediator phenolic structures of lignin (Ruiz-Dueñas et al. 2021). Besides biochemical studies, the essential role of class II peroxidases in lignin decomposition was also supported by phylogenomic studies. Diversification of this protein family correlates with diversification of WR fungi and the several transitions from a WR to BR or EM trophic modes almost always went along with a loss or a sharp contraction of class II peroxidase gene family in the fungal genomes (Kohler et al. 2015; Martin et al. 2016; Nagy et al. 2017; Miyauchi et al. 2020; Ruiz-Dueñas et al. 2021).

Despite the undoubted contribution of class II peroxidases to lignin decomposition, their exclusive role in this process is debated. Indeed, substantial lignin degradation seemingly occurs in the absence of organisms producing these enzymes, as in the case of the termite gut (Li et al. 2017). On the other hand, other microbial enzymes have been shown to be able to cleave phenolic and/or non-phenolic bonds of artificial lignin substrates (e.g. β-O-4-dimers = adlerol) and/or oxidize Mn^2+^ to Mn^3+^ and therefore behave similarly to either class II LiP/VPs or MnPs (Fernández-Fueyo et al. 2015). One such enzyme family is that of “dye-decolorizing peroxidase” (DyP) (pfam no. PF04261, E.C. 1.11.1.19) (Celis and Dubois 2015). These heme-containing peroxidases are unrelated to class II peroxidases and were identified for their unique ability to oxidize high-redox potential anthraquinone dyes (Kim et al. 1995), a property which makes them good candidates for the remediation of dye-polluted waste-water (Scheibner et al. 2008). DyPs present a ferredoxin-like fold (Singh and Eltis 2015), which is unique among peroxidases, and are present in all three domains of life (*Bacteria*, *Archaea*, and *Eukarya*) as opposed to class II peroxidases that are restricted to the *Eumycota* (true fungi) (Zámocký et al. 2012; Celis and Dubois 2015). Global phylogenies of the DyP (sub)family show that, with very few exceptions, fungal sequences are monophyletic and form a well-supported clade that does not include sequences from other taxonomic groups (Sugano 2009; Zámocký et al. 2015).

Different levels of biochemical information exist for at least 13 fungal DyPs (12 from *Basidiomycota*, one from *Ascomycota*), including 3D structures (based on protein crystals) for two of them (Yoshida et al. 2011; Strittmatter et al. 2013), identification of amino acids participating to catalysis through site-directed mutagenesis and biophysical measurements (Linde et al. 2015b) as well as reconstruction and characterization of ancestral fungal sequences (Zitare et al. 2021).

DyPs can form surface protein radicals (surface-exposed tryptophan radicals) via a long-range electron transfer (LRET) and are hereby able to oxidize phenolic compounds and synthetic dyes. In *Pleurotus ostreatus Pos*DyP4, a Mn^2+^-oxidation activity located on a different surface site, surrounded by acidic amino acids, was identified (Fernández-Fueyo et al. 2015). This activity is analogous to that accomplished by MnPs and VPs (Fernández-Fueyo et al. 2018), and Krahe et al. (2020) were able to use a closely related DyP of *Pleurotus sapidus* with Mn^2+^-oxidation activity to cleave alkenes (aryl alkenes (*E*)-methyl isoeugenol, *α*-methylstyrene, and *trans*-anethole) in the presence of Mn^2+^, which could be relevant in flavor production. A further Mn^2+^-oxidizing DyP activity has been recently reported for the first characterized ascomycetous DyP from *Xylaria grammica* (Kimani et al. 2021).

The roles of DyPs in fungal biology have never been addressed by forward genetic approaches, and they have been proposed to contribute to the detoxification of naturally occurring soil-borne or plant metabolites (Valette et al. 2017), including natural anthraquinone derivatives (Sugawara et al. 2019). They have also repeatedly been suspected to participate in lignin decomposition and in the oxidation of lignin-related compounds (Liers et al. 2010; Celis and Dubois 2015; Cragg et al. 2015; de Gonzalo et al. 2016; Catucci et al. 2020; de Eugenio et al. 2021), despite the fact that fungal class II peroxidases perform far better on “lignin substrates” than bacterial or fungal DyPs (Linde et al. 2021). In support of their putative roles in lignin oxidation, fungal DyPs have been also often reported as secreted enzymes, i.e. extracellularly acting biocatalysts. Several of them were purified from fungal culture filtrates, and they have been identified as well in the secretomes of fungi growing on lignin-rich substrates (Salvachúa et al. 2016). From an evolutionary perspective, as for class II peroxidases and other gene families, the fungal DyP (sub)family diversification parallels the evolution of WR fungi and regresses in BR and EM lineages (Nagy et al. 2017).

In the absence of forward genetic data and in the presence of uncertain biochemical activities, the objective of the present study has been to further evaluate the potential biological functions of fungal DyPs, including their putative contribution to lignin decomposition using phylogenetic and correlative approaches. Two strategies were followed. One was to establish a comprehensive view on the evolution of the DyP (sub)family in the fungal kingdom, using available genomic data, and to confront these data to our current knowledge on DyPs’ catalytic mechanisms and evolution of trophic modes in fungi. A second strategy was to isolate new fungal DyP genes expressed in different environments (soils and decomposing wood) in an attempt to characterize potentially divergent DyP sequences distantly related to sequences present in public databases. The latter approach could potentially identify new DyPs with potentially useful catalytic properties for novel applications in biotechnology.

## Materials and methods

### Species and sequence datasets

Publicly available DyP sequences were extracted from GenBank and the JGI Mycocosm portal (https://genome.jgi.doe.gov/programs/fungi/index.jsf; Grigoriev et al. 2014) databases as well as from our own unpublished whole-genome sequences. DyP protein sequences were identified in public databases using BlastP searches (Altschul et al. 1990) using as queries the protein sequences of two biochemically characterized DyPs from *Auricularia auricula-judae* (accession No. JQ650250) and *Bjerkandera adusta* (CDN40127.1). A threshold E-value of 10^−5^ was retained for the inclusion of the sequences in the dataset. DyP sequences extraction from the unpublished genomes was performed using HMMER (http://hmmer.org/). A HMMER profile was first created starting from a Clustal Omega alignment (Sievers and Higgins 2014) of 150 DyP sequences from the public databases. Phylogenetically-related bacterial DyP sequences, all belonging to the DyP clade C (Zámocký et al. 2015), were identified following blastp searches using as queries different sequences representative of the phylogenetic diversity of the fungal DyPs.

### Phylogenetic and network analyses

Only putative full-length protein sequences, starting with methionine and ending with a stop codon, corresponding to CDS longer than 1000 bp and without gaps in the regions where amino acids essential for catalysis have been identified (as described in Linde et al. 2015a), were used for a phylogenetic reconstruction of the fungal DyP (sub)family. In the case of the *Aspergillus* and *Penicillium*, genera for which the genomes of numerous species are available, only sequences from *Aspergillus oryzae* and *Penicillium italicum* were included in the phylogenetic analysis. Sequences were aligned using MUSCLE (Edgar 2004). ModelFinder (Kalyaanamoorthy et al. 2017), as implemented on the IQ-Tree web Server (http://iqtree.cibiv.univie.ac.at/; (Trifinopoulos et al. 2016)), was used to find the best substitution model (LG + R). IQ-TREE Web Server was also used to perform a maximum likelihood phylogenetic analysis (Nguyen et al. 2015). One thousand bootstrap replicates were performed to assess tree topology.

A fungal species phylogeny that encompassed all examined fully-sequenced basidiomycetous species was obtained by modifying a publicly available phylogenetic tree (#Tr106951, Zhao et al. 2017), built using an alignment of six genes, downloaded from TreeBase (www.treebase.org/). Species whose genomes have not been sequenced were removed from the original six-genes alignment while the corresponding six genes from newly sequenced species were added to it to construct a new species tree following the approach presented in Zhao et al. (2017). Species taxonomy was assigned using the MycoBank database (www.mycobank.org). Both gene and species trees were edited using the online software iTOL (https://itol.embl.de; (Letunic and Bork 2016)). We inferred gene loss and duplication along the species phylogenetic tree using a comparative genomics method with NOTUNG 2.9 as described in the original article of Chen et al. (2000).

To visualize similarities between fungal DyP protein sequences, a sequence similarity network (SSN) was computed using the EFI-Enzyme Similarity Tool (https://efi.igb.illinois.edu/efi-est/; (Gerlt et al. 2015)) starting from the complete set of DyP protein sequences. The lowest pairwise alignment score limit for the output file was set to 66. The full-network output file was visualized with Cytoscape 3.5.1 (Shannon et al. 2003). The network was represented with an “organic” layout, and only edges with a pairwise alignment score above 110 were visualized. To further evaluate the potential contribution of agaricomycetous DyP genes to organic matter (e.g. wood) and more specifically, to lignin degradation, we compared the prevalence of the different DyP clades in polyphyletic species groups of *Agaricomycota*, which differed with respect to their trophic modes (“trophic guilds”). Using the FUNguild (Nguyen et al. 2016) and experts’ opinions, we defined four trophic modes. “White-rot” (WR), sensu stricto encompasses saprotrophic or facultative pathogenic, lignicolous species (living on wood and coarse woody debris), which degrade lignin enzymatically to produce a “white-rot” phenotype in compact wood (where finally whitish cellulose fibers prevail). All of these species possess mostly numerous class-II peroxidase genes (Mn, versatile, and/or lignin peroxidases) in their genomes (Floudas et al. 2012). “Brown-rot” (BR) species, although also lignicolous, produce a brown-rot phenotype that results from a selective degradation of cell-wall polysaccharides (cellulose and xylan/hemicelluloses) along with a nonenzymatic modification of the lignin polymer. “Ectomycorrhizal” (EM) species are mutualistic symbionts of mainly trees (larger woody plants) that have (almost) lost the ability to degrade plant cell walls (Shah et al. 2016). Finally, a fourth group named “other saprotrophs” (OS) encompasses all other species that are either litter-decomposing saprotrophs (e.g. *Agaricus bisporus*), coprophilic species (e.g. *Coprinopsis cinerea*), or endophytic ones (e.g. *Sebacina vermifera*). Since these OS species do not grow on compact wood, their modes of degrading the different plant polymers do not exactly fit into the white-rot or brown-rot definitions, although several authors classify several of these species as primarily white-rotters (Eastwood et al. 2011; Nagy et al. 2016).

### Prediction of sequence features

Prediction of N-terminal signal peptides for eukaryotic secreted proteins was performed with SignalP (https://services.healthtech.dtu.dk/service.php?SignalP-5.0; (Nielsen, 2017)) with default settings except for the D-cutoff value, which was set at 0.34 for both the noTM (transmembrane domain) and the TM networks. SignalP raw results were reported on the protein phylogenetic tree. The number of putative *N*-glycosylation sites was predicted using NetNGlyc (https://services.healthtech.dtu.dk/service.php?NetNGlyc-1.0) (Gupta et al. 2002). Other protein features, such as protein length, absolute numbers of specific amino acids, predicted protein isoelectric point (pI), and global protein hydropathy using the “grand average of hydropathy” (GRAVY), were predicted using tools of the sequence manipulation suite (https://www.bioinformatics.org/sms2/; (Stothard 2000)).

Principal component analysis (PCA) was performed to cluster DyP protein sequences according to their protein features in the R environment using the R 3.6.3 (R Core Team 2019). Principal components were extracted with the prcomp function of the R base package and visualized with the ggbiplot package v0.55. Kmeans analysis was performed with the kmeans function of the R base package. Variable’s (i.e. protein features) contribution to principal components (PC) was determined using the fviz_contrib function from factoextra v1.0.6 R package. Statistical differences were tested using the nonparametric Kruskal–Wallis test and Dunn’s post hoc tests with dunn.test package v1.3.4 (Dinno 2015). DyP abundance comparisons among different fungal guilds were performed with the Yates-corrected Chi-square test.

### Probes design for gene capture by hybridization

From 1267 publicly available fungal DyP DNA-coding sequences, we designed 69 (70 bp long) degenerated probes (Supplemental Table S1) with the KASpOD software (https://g2im. u-clermont1.fr/kaspod/index.php, (Parisot et al. 2012)). In silico probe coverage evaluation, on the 1267 fungal DyP sequences detects 94.2% of the ascomycetous sequences and 81.5% of the basidiomycetous ones (with four allowed mismatches). Oligonucleotides corresponding to probe sequences were synthesized with two flanking adaptor sequences for their PCR amplification and conversion to biotinylated RNA probes using the T7 RNA polymerase, as described in Bragalini et al. (2014).

### Environmental samples, RNA extraction, and cDNA synthesis

Grassland and forest soil, as well as deadwood samples from six different contrasted geographic sites in Italy and France, were included in this study (Supplemental Table S2). Four of them had already been described in Adamo et al. (2020) and one in Bragalini et al. (2014).

RNA from soil samples was extracted from 2 g of material using the RNA Power Soil extraction kit from MOBIO Laboratories (Carlsbad, CA, USA) according to the manufacturer’s instructions. Deadwood RNA was extracted from 100 mg of decaying wood following the protocol described in Adamo et al. (2017). Eukaryotic cDNAs were synthesized from 2 μg of total environmental RNA using the “Mint-2 cDNA synthesis and amplification kit” according to the manufacturer’s instructions (Evrogen, Moskow, Russian Federation). First-strand synthesis was initiated at the mRNA poly-A end using a modified poly-dT primer (CDS-4 M). All resulting cDNAs were bordered at their 5′ and 3′ extremities by the same M1 sequence (5′-AAGCAGTGGTATCAACGCAGAGT-3′) that can be used for priming cDNA PCR amplification.

### Gene capture by hybridization

DyP cDNA sequence capture was performed as already described in Denonfoux et al. (2013) and Bragalini et al. (2014). Briefly, 2 μg of heat-denatured cDNA were hybridized to 500 ng of a mix of biotinylated RNA probes for 24 h at 65 °C in microcentrifuge tubes. The probes/DyP cDNA hybrids were then captured on streptavidin-coated paramagnetic beads (Dynabeads M-280 Streptavidin, Invitrogen, Waltham, MA USA). After several washing steps to eliminate unbound cDNAs, the captured cDNAs were detached from the beads using 0.1 M NaOH and purified using the “Qiaquick PCR purification kit” (Qiagen, Hilden, Germany). Captured cDNAs were further amplified using “Encyclo Plus PCR kit” and the M1 primer and were purified using the “Qiaquick PCR purification kit.” Captured cDNA was then subjected to the second round of capture identical to the first one. The enrichment in DyP sequences along the capture protocol was evaluated via a semi-quantitative PCR using DyP-specific primers (Bragalini et al. 2014; Kellner et al. 2014).

### Sequencing of captured cDNAs and bioinformatics analysis of the sequencing data

Since known fungal DyP CDSs range in size from 1500 to 2000 bp, captured cDNA was the first size fractionated to sequence only putatively full-length cDNAs and to eliminate truncated and contaminating sequences. Fractionation of 1 µg amplified cDNA (between 1400 and 2000 bp) was performed using a “BluePippin” instrument (Sage Science, Beverly, MA, USA). Fractionated cDNA was quantified by fluorimetry using the “Qubit dsDNA HS Assay Kit” and “Qubit Fluorimeter 2.0” (Thermo Fisher Scientific, Waltham, MA USA) and re-amplified by PCR using the M1 primer and “KAPA HiFi *Taq* polymerase” (KAPA Biosystems, Wilmington, MA USA). The 50 μl PCR mix contained 10 ng of cDNA, 10 μl of 5X KAPA HiFi Fidelity Buffer, 1.5 μl of 10 mM dNTPs, 10 μl of the 10 μM concentrated M1 primer, and 1 U of KAPA HiFi *Taq* polymerase. After an initial denaturation at 94 °C for 3 min, cDNA fragments were amplified for 25 cycles comprising 20 s at 98 °C, 15 s at 66 °C, and 1 min at 72 °C. Amplification was terminated by a final elongation at 72 °C for 3 min. The “Agencourt® AMPure® XP kit” (Beckman Coulter, Brea, CA USA) was used to perform a final purification and partial size fractionation of the captured cDNA. A 0.5X concentration of beads was used in order to remove small unspecific fragments from the eluted material. Purified cDNA quantity was assessed using the “Qubit dsDNA HS Assay Kit” and “Qubit Fluorimeter 2.0”, while DNA purity was assessed by measuring the OD 260/OD 280 and OD 260/OD 230 ratios using a NanoDrop TM 2000 spectrophotometer (Thermo Fisher Scientific, Waltham, MA USA).

All captured cDNA samples were sequenced using the Illumina HiSeq 2000 2 × 250 bp technology (I.G.A. Technologies, Udine, Italy) using the Illumina MiSeq 2 × 250 bp standard protocol for samples preparation.

Adapter sequences were eliminated using Cutadapt (Martin 2011). Sequence quality was evaluated with Trimmomatic (Bolger et al. 2014); bases with quality lower than 20 were eliminated, and only sequences longer than 60 bases were kept. Trimmed sequences were assembled using IDBA-UD (Peng et al. 2012) and the default parameter. To obtain longer contigs, the resulting contigs were then assembled using CAP3 and default parameters (Huang 1999). For the identification of DyP sequences, a similarity search between known fungal DyP sequences and contigs was performed using DIAMOND v0.9.22.123 (Buchfink et al. 2015), with the BLASTx command in “sensitive” mode (i.e. a maximal e-value of 1.e^−05^ and a minimal identity of 50%). Matching cDNA sequences were translated using the Expasy (Gasteiger 2003) translate tool and further analyzed for the presence of the DyP peroxidase domain using ScanProSite and the Prosite database (de Castro et al. 2006; Sigrist et al. 2012). Identified DyP proteins were clustered using a 90% identity threshold using CD-HIT v4.7 (Fu et al. 2012). Sequences are available on NCBI with the accession numbers OM674899-OM674912.

## Results

### Global distribution of DyP genes in the fungal kingdom (Eumycota)

Among the 227 fungal genomes inspected, 454 DyP-coding genes were identified in 162 species belonging exclusively to the *Dikarya* (*Ascomycota* and *Basidiomycota*; Supplemental Table S3) and not in any other fungal taxonomic groups. In the *Ascomycota*, 134 DyP genes were identified in the genomes of 84 species, all from the *Pezizomycotina* and none from the *Saccharomycotina* and *Taphrinomycotina*. For *Basidiomycota*, DyP genes were absent from the early-branching clades of *Ustilagomycotina* (subphylum of smut fungi), and present in the *Pucciniomycotina* (rust fungi) and *Agaricomycotina*. In these latter groups, 306 sequences were identified in the genomes of 78 species (34% of inspected species). Predicted numbers of putative DyP-encoding genes per genome differed greatly, ranging from 0 to 1 up to 64 genes in the case of the basidiomycetous species *Sphaerobolus stellatus* (which is remarkable since this fungus contains also 78 genes of unspecific peroxygenases/UPOs, EC 1.11.2.1; Hofrichter et al. 2020). In *Ascomycota*, the number of predicted DyP genes never exceeded six per genome (e.g. *Paraconiothyrium sporulosum*). A detailed list of DyP genes identified in the inspected species is given in Supplemental Table S4.

### Evolution of fungal DyP proteins

**Fig. 1:**
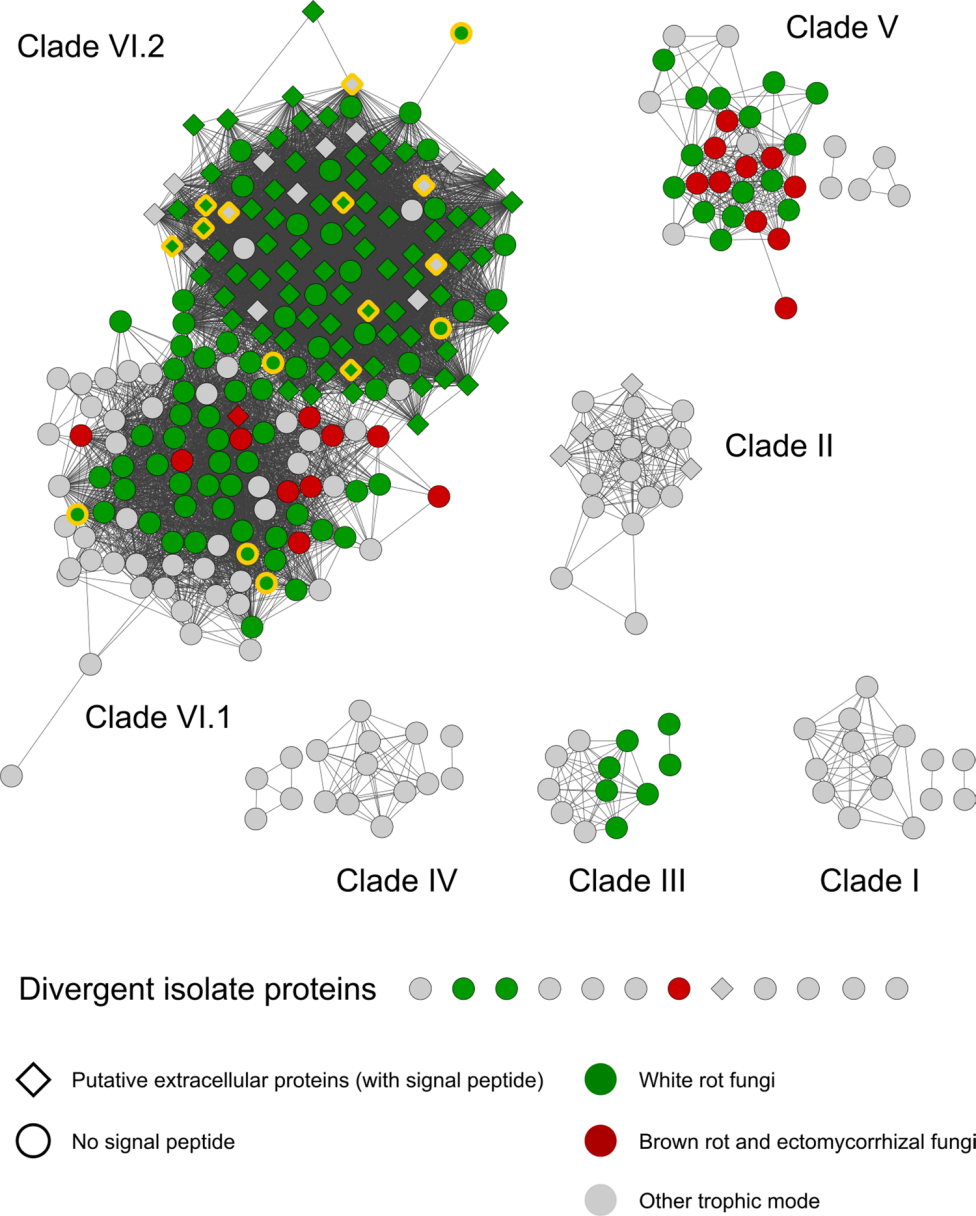
Sequence-similarity clustering of DyP protein sequences retrieved from fully sequenced fungal genomes and of biochemically-characterized fungal DyPs. Clustering using the EFI-EST server identified seven separate clusters (I − VI-1 and VI-2). Symbol shape indicates the presence (diamond) or absence (circle) of a predicted signal peptide. Symbol color indicates the trophic mode of the corresponding fungal species; red, white rot; green, brown rot or ectomycorrhizal; grey, other. Symbols with a yellow contour indicate biochemically-characterized enzymes

**Fig. 2:**
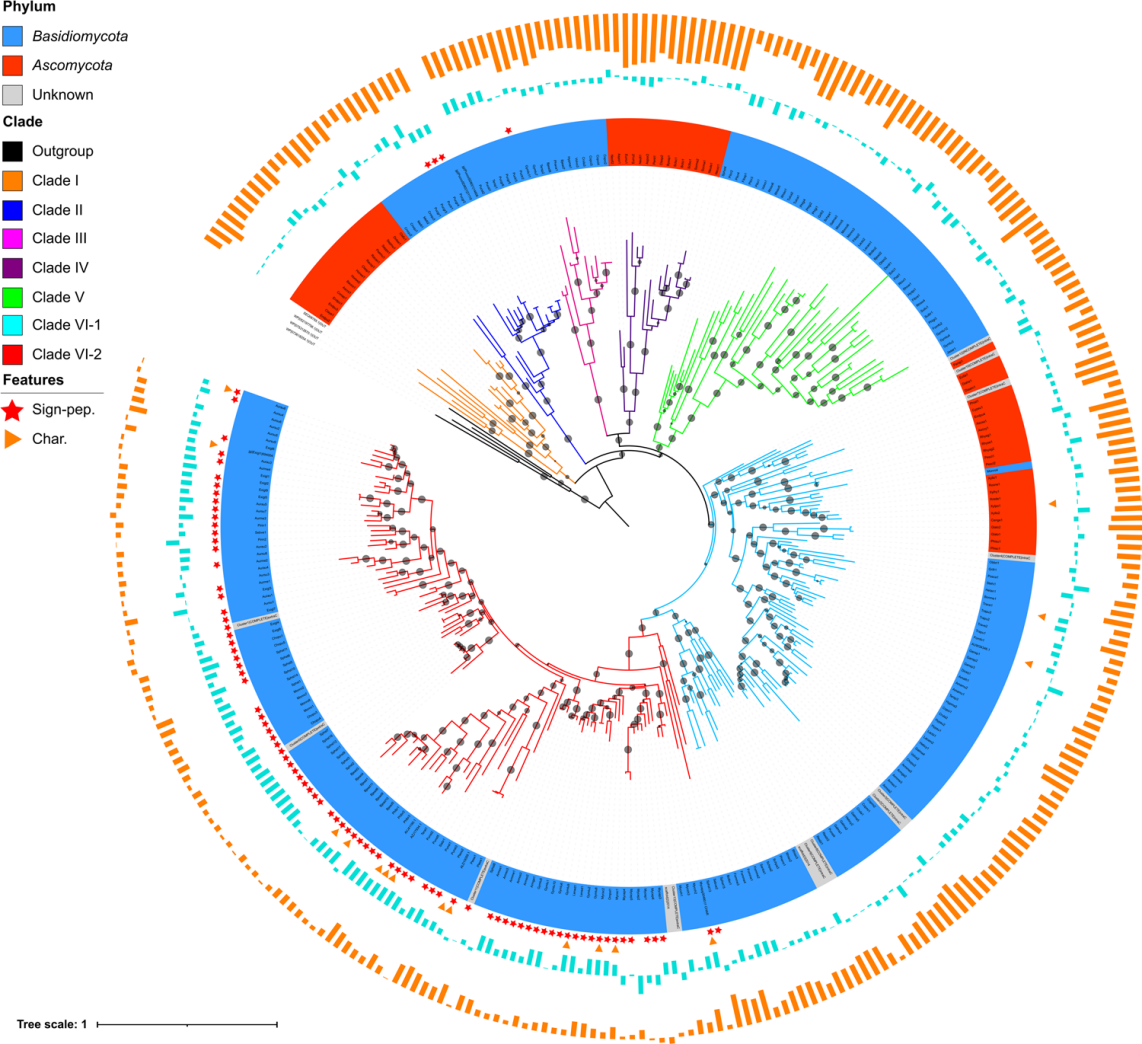
Phylogenetic analysis of the fungal DyP peroxidase family based on the alignment of 301 protein sequences retrieved from fully-sequenced genomes, corresponding to biochemically characterized enzymes, or encoded by environmental cDNAs isolated by gene capture by hybridization. Branch colors correspond to each of the sequence clusters illustrated in Fig. 1. The clade with black branches corresponds to bacterial sequences with the highest similarity values to fungal protein sequences. Grey dots indicate the bootstrap support (1000 replicates) of the corresponding branch; smallest dots, 75%; largest ones, 100%. The inner circle, the taxonomic origin of the sequences; blue, *Basidiomycota*; red, *Ascomycota*; grey, captured environmental sequences. Red stars, the presence of a putative signal peptide; red triangles, biochemically-characterized enzymes. Outer circle (orange bars), theoretical hydropathy (GRAVY) indices of the polypeptides (mostly negatives, a few positive values in cluster VI-2). Inner circle (light blue bars), pI values (expressed as pI-7)

DyP amino acid sequences were used to compute a sequence similarity network, which individualized seven well-separated clusters of sequences (Fig. 1) using a pairwise alignment score of 110. Following a phylogenetic analysis, each cluster defined one single monophyletic clade, with the exception of cluster VI-1, from which emerged clade VI-2 (Fig. 2). From the taxonomic point of view, while clade VI-1 encompassed sequences from both *Basidiomycota* and *Ascomycota*, other clades had a narrower distribution pattern as illustrated for clade II present in the plant pathogens belonging to the class *Pucciniomycotina* (with the exception of one sequence originating from the *Agaricales Termitomyces* sp.).

When representative sequences of each of the seven fungal DyP clades were individually used in blast searches to identify their closest relatives among bacterial DyP sequences, the same set of bacterial sequences was identified. These bacterial sequences all belonged to group C of DyP sequences as defined by (Zámocký et al. 2015). A selection of these bacterial sequences was included in the fungal DyP phylogeny. They all clustered in a single basal clade (Fig. 2).

**Fig. 3:**
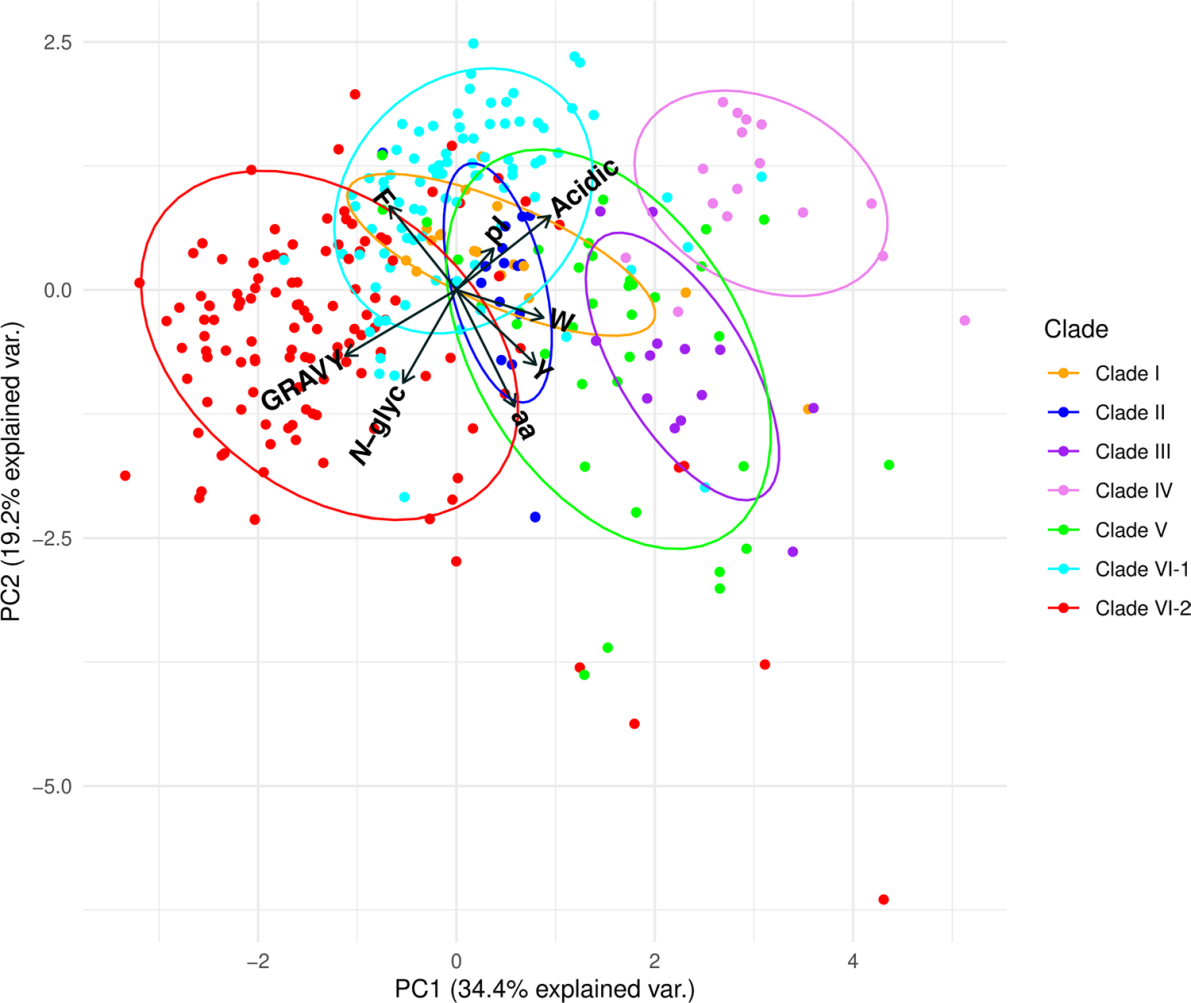
Principal component analysis (PCA) separates fungal DyP protein sequences (dots) belonging to different sequence clusters (same color code as in Fig. 2) according to their structural characteristics (vectors: hydropathy index (GRAVY), theoretical isoelectric point (pI), number of predicted *N*-glycosylation sites (*N*-glyc), polypeptide length (aa), number of specific amino acid residues (acidic, F, W, Y))

For each fungal protein sequence, we predicted a number of features that either reflected the global protein structure (GRAVY hydropathy index, isoelectric point (pI), sequence length, number of putative *N*-glycosylation sites, number of acidic amino acid residues at the putative Mn^2+^ oxidation site), which could be related to catalytic activities as in the case of the number of aromatic residues (tyrosine, tryptophan, and phenylalanine), which can participate in long-range electron transfers (LRETs) (Supplemental Table S4). When incorporated in a PCA analysis, these different variables separated several of the proteins according to the clades they belonged to (Fig. 3). The first two axes of the PCA altogether explained 52% of the variation in the dataset. Clade VI-2 was particularly well separated from all other clades with significantly lower average pI values and significantly higher GRAVY indices (Fig. 2), as well as the number of putative *N*-glycosylation sites per polypeptide (Dunn’s post hoc test, *p* < 0.05) (Supplemental Fig. S1). Furthermore, 69% of the proteins affiliated with clade VI-2 also possessed a predicted N-terminal signal peptide for secretion (Fig. 2). Outside of clade VI-2, only four proteins from *Puccinia* spp., belonging to clade II, also processed a putative signal peptide. Interestingly, 13 of the 16 purified and characterized fungal DyPs identified in the literature belong to clade VI-2; the three others were affiliated with clade VI-1. Most of these characterized proteins indeed appeared to be extracellular as they were purified from fungal culture filtrates presenting a dye-decolorizing activity (toward Reactive Blue5).

Inspection of amino acid residues present at positions essential for catalysis in the AauDyPI enzyme from *A. auricula-judae* (Strittmatter et al. 2013; Linde et al. 2015b) highlighted differences between the different clades (Supplemental Fig. S2). Positions in the distal side of the heme pocket are well conserved across all the clades, except in the case of Clade III, where the aspartate residue is replaced by a glycine one (position 168 in AauDyPI), and phenylalanine frequently replaced by valine (position 359). In the case of the proximal side of the heme pocket, while the histidine and aspartate residues, implicated in Fe^3+^ ligation as well as in peroxide-binding and cleavage, are well conserved across the clades, the central valine (position 253) residue present in the *AauDyPI* protein is frequently replaced by isoleucine, methionine, or phenylalanine residues, the latter two being predominant in clades I and IV, and III, respectively (Supplemental Fig. S2). Finally, the radical-forming residues tyrosine and tryptophan, probably involved in LRETs, present at positions 337 and 377 of the AauDyPI protein are conserved in all clades, except in clades VI-1 and VI-2, where they are frequently replaced by phenylalanine, another aromatic amino acid that can participate in LRETs (Acebes et al. 2017).

### DyPs and the evolution of trophic modes in the Agaricomycotina

**Fig. 4:**
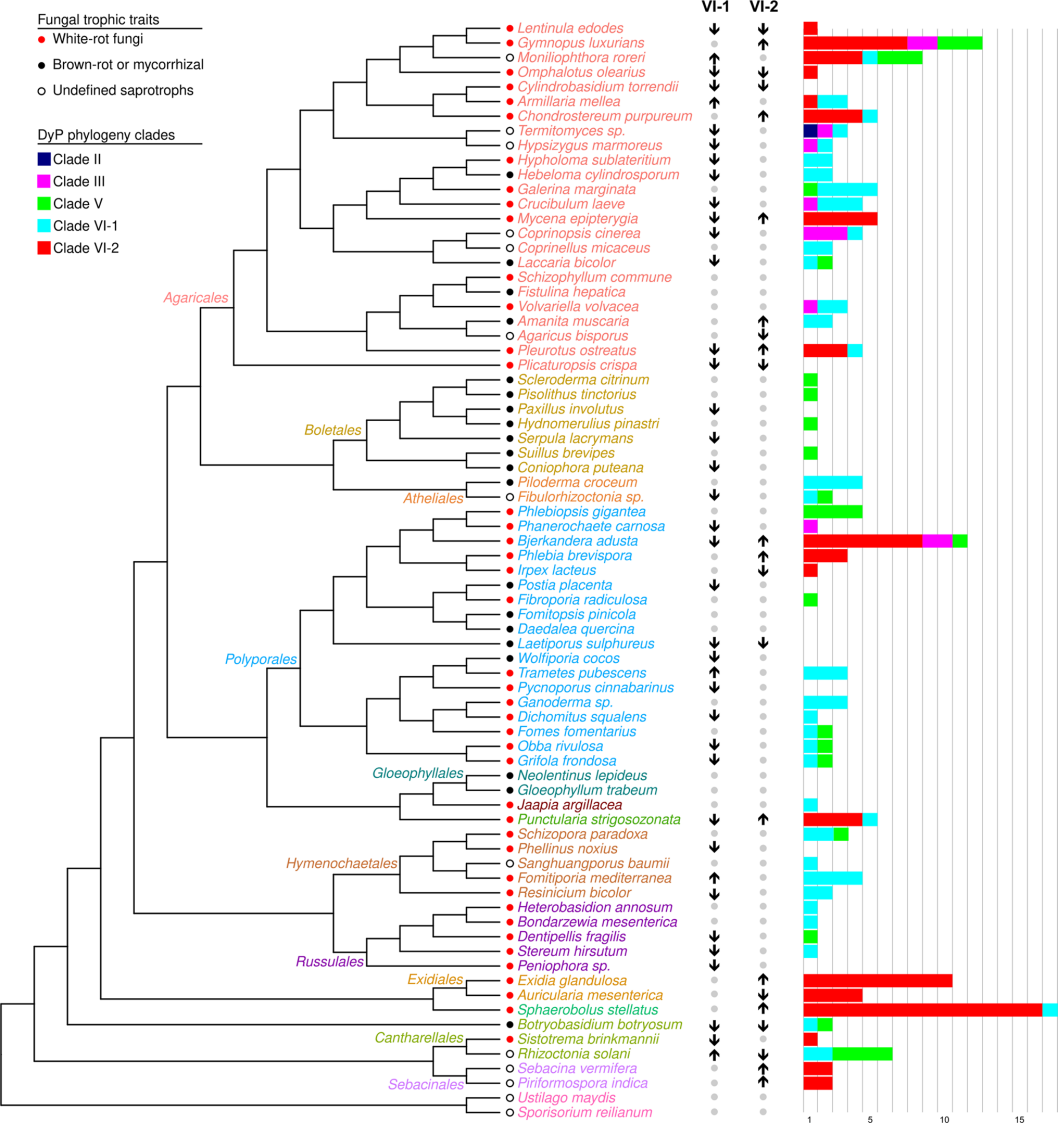
Phylogenetic tree of 128 fully-sequenced *Agaricomycotina* species and two *Ustilaginomicotina* (used as outgroup) computed based on a six genes sequence alignment according to Zhao et al. (2017). Species names are colored according to the order they belong to. Colored dots associated with species names indicate their trophic mode, as reported in the legend. The number of predicted DyPs in each sequenced genome and their distribution in the different sequence clusters (Fig. 1 and 2) are presented in the bar chart. Arrows pointing downwards or upwards indicate significant expansion or reduction in the number of DyP genes affiliated to clusters VI-1 and VI-2 at the leaves of the tree (according to NOTUNG)

We constructed a species phylogenetic tree that included 128 *Agaricomycotina* species and two species belonging to *Ustilaginomycotina* (as an outgroup) with fully sequenced genomes (Fig. 4), based on the tree published by Zhao et al. (2017). According to Zhao et al. (2017), the topology of this tree reflected the time-dependent divergence between the main agaricomycetous orders. Mapping the occurrence of DyP genes and clusters on this consensus species tree showed that most major agaricomycetous DyP clades (II, III, V, and VI, see Fig. 2) diverged early during the evolution of this taxon, being already present in all the most basal taxa of the *Agaricomycotina*, such as *Cantharellales*, *Sebacinales*, *Auriculariales*, and *Tremellales* (Hibbett et al. 2007). The tree also illustrates the recurrent loss, but also duplication, of several of the DyP clades with a differential distribution among the main agaricomycetous orders. For example, current genomic data suggest the loss of DyP genes belonging to clades VI-1 and VI-2 in the ancestor of the *Boletales*, which seemingly only possess members of clade V.

DyP phylogeny did not match fungal species phylogeny. One single species could host genes belonging to up to three clades (as in the case of the agaric species *Gymnopus luxurians* or the polypore *B. adusta* that both possess DyPs from clades VI-1, V, and III), and the presence of genes encoding DyPs representative of one clade never excluded the presence of a gene of another clade. We predicted the number of gene loss or gene gain events, using the NOTUNG software, separately for the most sequence-rich clades VI-1 and VI-2. Diversification at the level of the tree leaves (species-level) of clades VI-1 and VI-2 was not systematically coordinated between DyP clades (Fig. 4).

To further evaluate the potential contribution of agaricomycetous DyP genes to organic matter conversion, and more specifically, to lignin degradation, we compared the prevalence of the different DyP clades in *Agaricomycotina* with respect to their trophic modes. The first observation from the cluster analysis is that clade VI-2 (putatively secreted extracellular DyPs) encompassed sequences exclusively from WR and OS species and none from BR or EM ones (Fig. 1). This distribution is specific to clade VI-2. Indeed, all other clades with agaricomycetous sequences (clades II, III, V, and VI-1) contained sequences from WR, OS, BR, and EM species (the latter being absent from clade III). Although clade VI-2 sequences are absent from nearly two-thirds of the species classified as WR, and more WR species have been sequenced compared to BR and EM ones (Fig. 4), Chi^2^ tests strongly support (*p* < 0.01) the higher prevalence of clade VI-2 sequences in WR (or WR and OS) species than in BR and EM ones. On the contrary, we did not find evidence for a higher prevalence of other clades in groups of species presenting a specific trophic mode (WR, OS, BR, or EM; Chi^2^ test, *p* < 0.5).

### Diversity of fungal DyPs expressed in the environment

A set of 69 biotinylated degenerated RNA probes, whose sequences spanned the known phylogenetic diversity of fungal DyP sequences, was designed and used to capture homologous cDNAs synthesized from mRNA directly extracted from different environmental matrices (decaying wood, forest and grassland leaf-litter, and soils). DNA recovered after two successive rounds of capture (enrichment) was randomly sequenced, and the reads were successfully assembled to recover 14 full-length or nearly full-length DyP cDNAs. All of these environmental sequences encode different DyP not present in the database. They were clearly affiliated with either clade VI-2 (5 sequences) or clade VI-1 (9 sequences) (Fig. 2). In clade VI-1, three environmental DyP sequences were related to reference sequences from *Ascomycota* and six to sequences from *Basidiomycota*. We affiliated “cluster 4” to *Basidiomycota*, although also close to *Ascomycota* sequences in the phylogeny. In clade VI-2, three of the five protein sequences had a predicted N-terminal signal peptide.

## Discussion

We provide an exhaustive and comprehensive view of DyP-encoding genes distribution during the evolution of the *Dikarya* fungi, using all fungal genomes publicly available at the start of the study. Fungal (*Eumycota*) DyP coding genes of the clade D, as defined by Zamocky et al. (2015), are present in some, but not all, *Dikarya* species. Search for the most similar bacterial sequences and their placement in the fungal DyP phylogenetic tree suggest that fungal sequences may have originated from a single horizontal transfer of a bacterial class C gene to an ancestor of the *Dikarya* more than 400 My ago (Taylor et al. 2004), followed by multiple independent events of gene loss, duplication and diversification during the course of evolution of this taxonomic group.

The access to a large (more than 450) number of fungal DyP sequences allowed the identification of distinct clades, defined on the basis of sequence similarities that diverged from each other at different periods during the evolution of the *Dikarya*. In the *Agaricomycotina*, and to a far lesser extent in the *Pezzizomycotina*, DyP diversification did not parallel taxa diversification since DyP genes belonging to different (up to three) clades can co-exist in the genome of a single extant fungal species. The four main DyP clades (III, V, VI-1, and VI-2) present in extant *Agaricomycotina* have thus followed different and apparently independent evolutionary trajectories, being independently eliminated, recruited, or amplified in specific lineages. It seems, for example, that clade VI-2 experienced more frequent episodes of both gene diversification and loss than clade VI-1. Consequently, clade VI-1 is more prevalent in extant agaricomycetous species compared to clade VI-2, but with an average lower number of copies per genome (1.5 versus 3.9). These independent evolutionary trajectories suggest that enzymes from each of the clades may participate in different functions and are under different selective pressures.

DyP clades, which form distinct clusters in network analyses, can also be, to some extent, distinguished from each other based on “global” protein characteristics (hydropathy indices, isoelectric points, absolute numbers of aromatic residues, and abundance of *N*-glycosylation sites or the presence/absence of an N-terminal signal peptide for secretion). As observed for other enzyme families, including enzymes implicated in biomass degradation (Aspeborg et al. 2012; Viborg et al. 2019), these observations suggest that the divergence between DyPs could be associated with significant changes in catalytic properties, substrate range, and cellular functions (Zallot et al. 2021). It is, however, difficult to speculate on the nature of these changes since current structural (e.g. 3D structure) and functional information on fungal DyPs are fragmentary and with regard to related enzymes belonging exclusively to the phylogenetically-related clades VI-1 and VI-2. Enzymes belonging to this latter clade share a number of very distinctive structural features that clearly distinguish them from those of other clades. The most striking one is the presence of a sequence peptide that suggests secretion through the conventional endoplasmic reticulum pathway. Although SignalP may sometimes fail to predict signal peptides, we are confident that in our case, this should be a marginal problem as the presence of a signal peptide was predicted for almost all of the sequences of clade VI-2. This hypothesis is further supported by the presence in clade VI-2 proteins of a higher number of putative *N*-glycosylation sites, a common feature of endoplasmic reticulum-secreted proteins (Medus et al. 2017). Although it is tempting to hypothesize that the emergence of clade VI-2 corresponded to a transition from an intracellular to an extracellular status, this hypothesis would need further experimental validation. On the one hand, a majority of the biochemically characterized fungal DyPs have been purified from culture filtrates that precisely belong to clade VI-2 (see Fig. 2). On the other hand, novel DyPs also purified from culture filtrates belong to clade VI-1, from which emerged clade VI-2. Clade VI-1 is the only clade that encompasses both ascomycetous and basidiomycetous sequences, and one of the characterized enzymes was from the wood-rot *Ascomycota X. grammica*. It could thus be hypothesized that clade VI-1 DyPs are either intracellular or extracellular, secreted through non-conventional secretion systems as proposed for a rhamnosidase of *Xylaria polymorpha* and other fungal proteins (Nghi et al. 2012; Prudovsky et al. 2003). Acquisition or loss of structural elements favoring secretion of DyPs is also documented in the bacteria, where type A DyPs possess a Tat secretion signal not found in the other bacterial clades such as clade C from which fungal DyPs may derive (de Gonzalo et al. 2016). Secreted type A bacterial DyPs are suspected to be involved in chemical lignin modification (Sugano and Yoshida 2021).

Besides clade VI (VI-1 and VI-2) DyPs, no functional information exists for DyPs belonging to other clades, including their putative cellular localization, although several, but not all, DyPs of rust fungi (*Puccinomycotina*) possess signal peptides. We suggest that future functional studies should focus on these other clades, not only to precise their cellular localization but also on their catalytic activities and substrate range to understand their cellular functions and evaluate their potential use as catalysts in biotechnology.

Regarding the most debated role of fungal DyPs, i.e. their contribution to lignin degradation, our correlative analyses suggest a facultative contribution of a subset of these enzymes in this process, as suggested in Nagy et al. (2017), who considered the fungal DyP family as a whole without splitting it in distinct clades with different evolutionary histories. We show a strong association between clade VI-2 and species of *Agaricales* classified as white-rotters or litter-decomposers (soil saprotrophs) that, for many of them, can degrade lignin and humic substances (Steffen et al. 2000, 2002). Intuitively, enzymes active on bulky extracellular polymers need to be secreted as we hypothesized that they are in the case of clade VI-2 DyPs. This strong association contrasts with the complete absence of this clade from the genomes of either brown rot or ectomycorrhizal species sequenced thus far. In the case of the *Boletales*, a taxonomic group composed of BR and ECM species, the loss of clade VI-2 DyPs could even represent an ancestral characteristic. BR and ECM species can, however, possess DyPs belonging to other clades. If clade VI-2 DyPs participate in lignin degradation/modification, this role may be rather accessory as (i) many WR species lack these enzymes and (ii) species that possess clade VI-2 DyPs also possess class II peroxidases, including MnPs, VPs, and/or LiPs. Besides *Agaricales*, clade VI-2 DyPs are also present in saprotrophic or plant symbionts/endophytes of the orders *Cantharellales/Sebacinales* that are not considered WR species (Nagy et al. 2016) but possess a rich set of genes encoding plant cell-wall degrading enzymes in their genomes (Kohler et al. 2015). Among these species is *Sistotrema* sp. (*Cantharellales*), sometimes considered a white-rot fungus although not possessing class-II peroxidases (Riley et al. 2014).

Contribution of clade VI-1 enzymes to lignin degradation by the wood-rot ascomycete *X. grammica* has also been suggested as one corresponding enzyme purified from culture filtrates of this species possesses a Mn^2+^-oxidizing activity (Kimani et al. 2021). Incidentally, we observe that several other ascomycetous species possessing clade VI-1 DyPs are associated with decaying wood (e.g. *Ascocoryne* sp*.*, *Diaporthe ampelina*, *Eutypa lata*, *Kretzschmaria deusta*, *Rosellinia necatrix*, and *Xylaria* spp.). These genera and species could be targeted to study the transcription profile of their DyPs when grown on lignocellulosic substrates, their (extra)cellular localization, and substrate specificity. Other ascomycetous species possessing clade VI-2 DyPs were however not associated with rotting wood, this is also the case for all ascomycetous species processing clade I or IV DyPs, the two other clades present in this phylum.

Regarding DyPs expressed in the environment, we successfully assembled 14 complete or nearly complete DyP sequences following sequence capture of cDNAs retrotranscribed from soil and decaying wood RNAs. All DyP sequences were affiliated to either clades VI-1 or VI-2 despite the fact that capture probes also targeted sequences from the other fungal DyP clades. Clades VI-1 and VI-2 sequences represent altogether about two-thirds of all DyP present in databases. The exclusive presence of clades VI-1 or VI-2 DyPs among environmental sequences suggests that fungal species harboring DyPs belonging to these clades are either overrepresented and/or that the corresponding genes are overexpressed in the studied environments. These observations are comforted by the data of Kellner et al. (2014), who used PCR to obtain partial DyP sequences from different forest soils. Affiliation of these 61 partial sequences to the different clades showed that 81% originated from clades VI-1 (42%) and VI-2 (24%), while a minority of the sequences came from clades I (4 sequences), III (6), and V(1). The absence of environmental sequences that define novel DyP clades may signify that fungal genome sequencing may have already captured the entire phylogenetic diversity of the DyP D subfamily or those additional clades are too divergent to be captured by the designed probes or too rare to be easily identified using this approach. The occurrence of expressed clades VI-1 and VI-2 DyPs, from both basidiomycetous and ascomycetous taxa in organic matter (OM)-rich environments (leaf-litter, uppermost soil) reinforces our hypothesis for their involvement in OM degradation and at the same time should incite us to characterize the catalytic properties of the more enigmatic and somewhat structurally divergent members of clades I to V.

## Supplementary Information

Below is the link to the electronic supplementary material.

Supplementary file1 (PDF 1239 KB)

## Data Availability

Sequences generated during the current study are available in the NCBI-nr repository, with accession numbers OM674899-OM674912. All accession numbers of the sequences analyzed during this study are included in this published article (Supplementary Table S4).
