# Supplementary material for: Fungal dye-decolorizing peroxidase diversity: roles in either intra- or extracellular processes
Source: Appl Microbiol Biotechnol. 2022 Apr 18;106(8):2993–3007. doi: 10.1007/s00253-022-11923-0 (PMC9064869; doi:10.1007/s00253-022-11923-0)
Supplement: Supplementary file 1 — Supplementary file1 (PDF 1239 KB) [file 253_2022_11923_MOESM1_ESM.pdf]

Online Resource 1 - Supplementary Information

Fungal dye-decolorizing peroxidase diversity: roles in either intra- or extracellular processes?

Martino Adamo, Sophie Comtet-Marre, Enrico Büttner, Harald Kellner, Patricia Luis, Laurent Vallon, Rocio Prego, Martin Hofrichter, Mariangela Girlanda, Pierre Peyret, Roland Marmeisse.

File contents

- Supplementary Tables

Table S1 – Sequences of the probes used for gene capture by hybridization.
Table S2 – Study sites and samples description.
Table S3 – List of species included in the study.
Table S4 – List of proteins included in the study.

- Supplementary Figures

Figure S1 - DyP features compared by clade.
Figure S2 - aminoacids present at DyPs key active site positions

- Genome references – cited in Table S3

Table S1 – List of the degenerated probes used in this study to target sequences encoding eukaryotic DyP-type fungal peroxidases. Probes are specific for *Ascomycota* or *Basidiomycota*. Coverage values correspond to the percentage of known fungal DyP sequences (1267) whose sequences can be recognized in silico by each of the probes.

**Degenerated probe sequence (5'-3' oriented)**	**Taxa**	**Coverage**
CAGACAACATTCAGGGCAGCATCTGGCCCCGTCTRCCYAARWMYTATGAATCCTAYCTSTTYTTCAAGAT	*Ascomycota*	11.11
ACACCTYAGAGATATYCTYGACAATGGRGARRTCACCACYGGCACTCAATGCGAGRACCACCTGARGTCA	*Ascomycota*	8.19
TCGGTCGACACGGACAACATCCAAGGGAGCATTTGGCCCCGTCTCCCTAAATACTATGAAACCTACCTCT	*Ascomycota*	6.43
GGGCCACAAAGGTGAYCCSAATMTGAAAAGAGGCCCTGAGTGGGCTAAGGAGGGCAGTTTCCTCGTCTTT	*Ascomycota*	2.34
AGTGGTYGACACYGACAACATCCAGGGMAGCATCTGGCCYCGTCTCCCCAAGTWYKMKGAATCMTACCTC	*Ascomycota*	9.36
CGATATTAATAAGGACAACATYCAAGGCAACATCTGGCCGGGTKTGCCGAAGCTTYATGAATTCTTTTTA	*Ascomycota*	4.09
TGTCCCGGCCAAGATTCWGSCCTTGCCAGGCGTYAACATTGCCTTTGCCTCGACTGGTCTAGCAGCTCTG	*Ascomycota*	8.19
ACATCCGAAACGGATTCAACTTCCTGACTACCCGCTGGGCAAGCAACCACCACTTCCCCGACCGCAAGAG	*Ascomycota*	12.28
ATGGTGTTCCGCACATATGAACAAAGAACACCGGAGTTCGTTGCCTGGTGTGCCGCGAACACCAAGAAAT	*Ascomycota*	2.34
GTCAAGACACMATCGAYCAAGGCRYMATCCTCTGCGGCCGMCCCGGCGACACYCAAGCCTCCAYCCGTCA	*Ascomycota*	4.09
AAGGCGACATCCTCCTCAACGGTCTCCCCAAAGAAGTCGAAACGTTCTGGTTCTTCGACATCGTCGATGC	*Ascomycota*	2.92
ATCACTGATCTCAGCAATGTCCAAGGSGACATTCTCCTCAARGGCTTGACGAAAGAGGTCGAGACYTTTT	*Ascomycota*	2.34
CAAGACCCTTCGCCTCAGCCGGATTGGGCGACGGAAGGTAGCTTCCTGGTATTCCGCAAGCTGCAACAGT	*Ascomycota*	2.34
ATGAATAACACGGATGCCTTCAACTAYMRCCCTRYTGACCAGAYCAAGTGYCCTTAYGCMTCTCAYATGC	*Ascomycota*	5.26
TGCCSTCTGAAGTCRACCCCGCCAACATCCAGGGCAGTATCTGGCCCCGTCTCCCMCGGTACTAYGAAAG	*Ascomycota*	4.68
TCAAGGCRMTGAAGGRRTATCTGACTGGCCCCCCTGATTAYCCGCCAGAGCTTGCCGGCCCTTGAGAGTT	*Ascomycota*	3.51
AATCTAATAACCATGAGCAATTCATCAAGAGCGGTGAACGTATCCATCCAGACTGAAAGTCCCCCGCTGT	*Ascomycota*	2.34
AGRCTWGAYCACCACCCMCCTCGRTCWATCGGTCTSCCTGATGGCAAATATCCMACCGAGGCMCGCATGC	*Ascomycota*	2.34
AGGAGCTYTGGGGAGCTAGGWTGATYGGTCGTTGGAAATCYGGGGCTCCTATAGCATTGTGTCCGTACAA	*Basidiomycota*	1.19
TGGYCTTTGYGCTRCARGGCGTGTATKCTGTMCCAAATCTTYCATCCAATGACCTGAAACCTAGACGAAC	*Basidiomycota*	1.19

CTCGWRRTAATCTTCCTGGAAGCAACGATCATKTATTCAGACGRGCTAGTATTMCYTATGGCGGRGARST	*Basidiomycota*	1.19
TACAAGGCGACATTTTGGTTGGMATGAAGAAACAGAAGGAACGCTTYGTCTTTTTCCAMGTCAATGACGC	*Basidiomycota*	2.58
GGGCCATCGACGGGASYTTCATGGCSTTCCGRCACYTGCARCAGAARGTGCCCGAGTTYMACGCCTACAC	*Basidiomycota*	2.78
CAYCYTSCCKCTGGATAACATCCARGGCGAYATCYTGRTYGGAATGAAGAAACAGAARGAACGCTTCGTC	*Basidiomycota*	1.99
TACCAGTCGAAGAYRTACACACTKCCRSAGTTYGTRATCCCRAAGGGCGGMGARTACTTCTTCATGCCCT	*Basidiomycota*	1.19
AACAYCYTSCCKCTGGATAACATCCARGGCGAYATCYTGRTYGGAATGAAGAAACAGAARGAACGCTTCG	*Basidiomycota*	1.59
TCGGCCTCAGCAACRTYCAAGGCGAYGTCATWCCYGGTCTSCCYAAGGCYCTCGAATWYTTCTACTACTT	*Basidiomycota*	1.39
GTGCGAGGCAGTCGCGCACAACGCCTCTCCTCACSARCTTYCCKGGRCARGCCCCGCTRCCTTCYMTRGA	*Basidiomycota*	0.99
CCTCTSAARSYRTCGGACTCAATCTCGATGATATYCAGGGYGACATYCTYGTKGGCATGAAGAAGGACAA	*Basidiomycota*	3.38
GCTCGAACAGAAYGTGCTTTTTCTCGAAGAYTATGTCAACAAGAACTGGCTGTCTATTCCAGCTRAGCCC	*Basidiomycota*	1.19
ACCGAGCTCTACTACTTCTTCCAAATCACCAACGCGTCAGATTTCCGCAAACATCTCATCAAATTCGCGC	*Basidiomycota*	0.99
CAGCGCGATTAGCAGGCGCACTTACAATCAGCTTGTTACMTGTGCTTACRAGGTCGAAGGAGACACCAGG	*Basidiomycota*	0.99
ATGCTTGCAGCGGATCCTAAGCGCAACAACGATTTCAAGATTGAAGGGGAGATCAACTCACAATTCCGCT	*Basidiomycota*	0.8
TTYARGCGCGACCTYGCGAACTACACMCCSACRACRTCCGAWGAYGTTCTTGACAACCTKCGCCAGATCA	*Basidiomycota*	1.59
GCACGCCGCCCACGAAAGAGGGCGTGTACCGCATCAAGCTGTATGGGCAGAGTCCGGAGCARTGGCGCAT	*Basidiomycota*	0.8
AATACAAGGGAAAGGAACACTTCGGATATCAGGACGGTGTATCYCAGCCATCGTTGAGAGGCTTAACCAC	*Basidiomycota*	0.8
CGGATCCYAAWCAAGCWTGGCTCAAAGAYGGRTCRTTTATGGYTTTCAGAGAGCTCCAACAAYTYGTACC	*Basidiomycota*	4.57
AAAAGGTCAAAAASTTGAYGCCRTSRAMAACTTGGGAGAYCCGGTRGATCAAGCARCGAAAAARTTAAAA	*Basidiomycota*	3.18
ACGCATGGCTCAAAGATGGCTCGTTTATGGTTTTCCGACAGCTCCAACAACTCGTACCCGAGTTTCAAAC	*Basidiomycota*	2.98
ACCTAACCGGGTCGATTTAAAAAACGTCCAAGGCGATATTATCATTGGTTTGCAAAAACGTTACGAGGCG	*Basidiomycota*	1.19
AGTCTCGACTAGCCTTCCGTCGAACATTGAAAGACAAGCTTCTGCCTCTGATCACCACCACTCAACAAGT	*Basidiomycota*	1.59
ATGCGCTGGTGGACTACCTGCGTCGCTCTCACTTCGTTARTACCTTACRCCCTCYTGCCCAGTTATGGGT	*Basidiomycota*	1.19
TCGTKCCCGAGTACAASAAGTKSCTSCTCGACAACGCCGTYCAGAGCCCSGCCGGMAAYCTCACSCAGCA	*Basidiomycota*	1.19

AGGAACAACAAKTTCGACTTYGGTGATTCCTTAGTTCGAGGCGATCAGACGAAGTGCCCTTTYGCTGCTC	*Basidiomycota*	0.8
GCGGCRCGCATGTTCGGYCGYTGGCCAAGYGGCGCACCAAYCGATCTCACSCCCTTYGCSGACGAYCCCG	*Basidiomycota*	1.39
TAGCGGCAACAACCATGGCRTATTCATCATCTGCTCTRRATYCCARSAGAGYTGCCTAKMKGAATCGAAC	*Basidiomycota*	1.79
CTTGGAYGGSATYTCCCAACCSGCRGTCAAGGAWTTCGACACGAAGCCSAACCCMGGMCAGGAGACCGTC	*Basidiomycota*	2.98
CGCMGCSTACCAGAGCAACATCGTYAACGGCTTCCARTTCATCCAGCACAGYTGGGCSAACAMYGTCRRT	*Basidiomycota*	2.39
GCCGGCCTCCCGAAGAAGGTCCAGCACTACCTGYTCTTCCAGATCGAYGACRAYGTCMMSGCHTTCAGGM	*Basidiomycota*	2.58
ATCCCACCCTTCGATCCTGCCAACGTCCAGGGCGACATCCTTGCCGGCCTCCCGAAGAAGGTCCAGCACT	*Basidiomycota*	1.59
ACTKGAYGGCAGCTTCCTYGCCYTCCGGTACCTCTTCCAGCTMGTTCCCGAGTTCRACACSTTCCTGAAG	*Basidiomycota*	1.79
GAAGCCTYSAGTCWCGCGCCGAKCTKYTGGGWGCGCGCATGGTCGGSCGCTGGAARTCGGGAGCGCCCAT	*Basidiomycota*	1.99
TCARKCRCGCGCSGATCTSCTGGGAGCGCGCATGGTYGGSCGMTGGAARTCGGGAGCGCCCATYGATCTG	*Basidiomycota*	3.78
GGSCGMTGGAARTCGGGAGCGCCCATYGATCTGACACCWACCGCGGAYGACCCCGCKCTCGGTGCCGAYG	*Basidiomycota*	1.39
TCCAAGGACGGYTCWTTCCTCGCGTTCCGCCAGCTTCAGCARCTCGTTCCMGARTTCCACAAGTACCTSA	*Basidiomycota*	1.19
CARAYGATATTCAAGGYGACATTCTCGTSGGWATGCAYAAGCARAAGCAGCTRTTCTACTTCTTCGCYAT	*Basidiomycota*	2.19
TTTGGAYCTTRAMAACATYCARGGYGAYATCTTGAGCGGCCTTCCAAAGAAGACCCAAACCTAYTTCTTC	*Basidiomycota*	1.79
TTCCATATTAGGTGACTTTATACTTGTTCCCATTCCTGGAAGCAAATCGACAGGCCAGATATATCCATCC	*Basidiomycota*	0.2
GAAAAGRAWGCCATCAGTTATGACCAGGTGGAAGCTCTTGTGACGAAGTGCTTGMGRCGGARCAAATAGT	*Basidiomycota*	0.4
ACGGGTARYTGGGTYCAGGGCTTYGCAGGAACAAGTRTTCAYGGCGTATTTYTGATTGCGTCYGATACSC	*Basidiomycota*	1.39
GCAAGACWCGYCCSCGTGCCGATCTCGTYGCRCCKGCSAACAGCATCATYCGCAGYGGMATCCCGTACGG	*Basidiomycota*	1.19
ACCGAAAGGAGGAGAATATTTCTTCGTGCCCTCAATATCAGGCCTTCGGGAGGTAATTGTCGGATAAACG	*Basidiomycota*	0.4
GARAGYGTCGGACTCAATCTCGATGATATCCARGGTGACATCCTCGTRGGCATGAAGAAGGACAAGGAAC	*Basidiomycota*	1.99
GACGACGTCCATCCGATGAGATCGAGCCGGAGTGCATCAGGGAGGCACCGCTGCTACCTGATCGTCCTTG	*Basidiomycota*	0.4
TTTCGGTGCTCGATTGATTGGACGGTGGAAGAGTGGTGCTCCTATCGATTTGGCACCTCTTCGTGATGAC	*Basidiomycota*	1.19
CCAGGCYTTCAGGCAGAGGCTCAAGCTGCTCATYCCTCTGATCACCACCACCMCYCAGGTCMWGGATGAC	*Basidiomycota*	0.99

GTACAACGTCTCTRTTRATYAGTCCTCCTGGAATTCCCCCKCTCCCTCAGGCTCRGCCRYCTGCTGCASR	*Basidiomycota*	1.19
GACGAAGATGGGTTCCCTRRWGGATGTCCACTTCAGAAYGACCCTRTRCGTCTTGTYAAYAGGATGGCWA	*Basidiomycota*	0.4
TGATGTCGAAGAAGAAGTAAGTTTGGGTCTTCTTTGGAAGGCCGCTCAAGATGTCGCCCTGAATGTTGTC	*Basidiomycota*	1.59

Table S2 – A brief description of the origin of the different environmental samples from which were extracted RNA for the capture by hybridization of fungal DyP cDNAs. Detailed description of the sites and of the sampling procedures are given in the references cited

**Sample source**	**Site name**	**Geographic coordinates**	**Elevation [m a.s.l.]**	**Habitat**	**Original study**
Forest soil	Puéchabon, Hérault, FRA	43.71N, 3.62E	270	*Quercus ilex* mediterranean forest	Bragalini et al. 2014
Forest soil	Venaria Reale, Torino, ITA	45.18N, 7.55E	300	European oak and oak-hornbeam forest	Adamo et al. 2020
Grassland soil	Venaria Reale, Torino, ITA	45.18N, 7.55E	300	*Molinia caerulea* wet meadow	Adamo et al. 2020
Decaying wood	Venaria Reale, Torino, ITA	45.18N, 7.55E	300	European oak and oak-hornbeam forest	Adamo et al. 2020
Forest soil	Bussoleno Torino, ITA	45.14N, 7.10E	500	Pannonian woods at *Quercus pubescens*	Adamo et al. 2020
Grassland soil	Bussoleno Torino, ITA	45.14N, 7.10E	500	Semi-arid *Bromus* rich meadow	Adamo et al. 2020
Decaying wood	Bussoleno Torino, ITA	45.14N, 7.10E	500	Pannonian woods at *Quercus pubescens*	Adamo et al. 2020
Forest soil	Chiusa di Pesio, Cuneo, ITA	45.18N, 7.55E	1280	Montane acid *Picea abies* forest	Adamo et al. 2020
Grassland soil	Chiusa di Pesio, Cuneo, ITA	45.18N, 7.55E	1280	Mountain hay meadow	Adamo et al. 2020
Decaying wood	Chiusa di Pesio, Cuneo, ITA	45.18N, 7.55E	1280	Montane acid *Picea abies* forest	Adamo et al. 2020
Forest soil	Vinadio, Cuneo, ITA	45.18N, 7.55E	2000	Alpine *Larix decidua* wood	Adamo et al. 2020
Grassland soil	Vinadio, Cuneo, ITA	45.18N, 7.55E	2000	Acid Alpine grassland	Adamo et al. 2020
Decaying wood	Vinadio, Cuneo, ITA	45.18N, 7.55E	2000	Alpine *Larix decidua* wood	Adamo et al. 2020
Forest soil	Arbigny, Saône-et-Loire, FRA*	46.71N, 4.91E	280	*Acer* and *Fraxinus* continental forest	This study
Grassland soil	Arbigny, Saône-et-Loire, FRA*	46.71N, 4.91E	280	Alluvional plan soil	This study
Decaying wood	Arbigny, Saône-et-Loire, FRA*	46.71N, 4.91E	280	*Acer* and *Fraxinus* continental forest	This study

* In the Arbigny site, soil and wood sampling followed the procedure described in Adamo et al. (2020)

Table S3 – List of the studied fungal species, with fully sequenced genomes. For each of the species are indicated the number of predicted DyP genes in their genomes (DyP); the number of predicted DyP polypeptides with a signal peptide for secretion (ext-DyP), the putative trophic mode (trait) of the species according to FUNguild (WR, white rot; BR, brown rot; MYC, ectomycorrhizal; OTH, other trophic modes); if the proteins of the species were included in the protein phylogenetic analysis; the protein network analysis and if the species was included in the species phylogenetic analysis.

**Species**	**Strain**	**Phylum**	**Order**	**Family**	**DyPs**	**ext-DyPs**	**Trait**	**pro_tree**	**netwk**	**sp_tree**	**Reference**
Agaricus bisporus	*Agaricus bisporus* var. *burnettii* JB137-S8	*Basidiomycota*	*Agaricales*	*Agaricaceae*	0	0	OTH	N	N	Y	Morin E et al.,2012
Agaricus bisporus	*Agaricus bisporus* var *bisporus* (H97) v2.0	*Basidiomycota*	*Agaricales*	*Agaricaceae*	0	0	OTH	N	N	N	Morin E et al.,2012
Amanita muscaria	*Amanita muscaria* Koide v1.0	*Basidiomycota*	*Agaricales*	*Amanitaceae*	2	0	MYC	Y	Y	Y	Kohler A et al.,2015
Amanita thiersii	*Amanita thiersii* Skay4041 v1.0	*Basidiomycota*	*Agaricales*	*Amanitaceae*	1	0	OTH	Y	Y	N	Hess J et al.,2014
Armillaria cepistipes	*Armillaria cepistipes* B5	*Basidiomycota*	*Agaricales*	*Physalacriaceae*	0	0	WR	N	N	N	Sipos G et al.,2017
Armillaria gallica	*Armillaria gallica* 21-2 v1.0	*Basidiomycota*	*Agaricales*	*Physalacriaceae*	4	2	WR	Y	Y	N	Sipos G et al.,2017
Armillaria mellea	*Armillaria mellea* DSM 3731	*Basidiomycota*	*Agaricales*	*Physalacriaceae*	4	2	WR	Y	Y	Y	Collins C et al.,2013
Armillaria ostoyae	*Armillaria ostoyae* C18/9	*Basidiomycota*	*Agaricales*	*Physalacriaceae*	4	1	WR	Y	N	N	Sipos G et al.,2017
Armillaria solidipes	*Armillaria solidipes* 28-4 v1.0	*Basidiomycota*	*Agaricales*	*Physalacriaceae*	4	2	WR	Y	Y	N	Sipos G et al.,2017
Ascochyta rabiei	*Ascochyta rabiei* ArDII	*Ascomycota*	*Pleosporales*	*Helotiaceae*	1	0	OTH	Y	Y	N	Verma S et al.,2016
Ascocoryne cylichnium	*Ascocoryne cylichnium*	*Ascomycota*	*Helotiales*	*Helotiaceae*	1	0	OTH	Y	N	N	This study
Ascocoryne sarcoides	*Ascocoryne sarcoides* NRRL50072	*Ascomycota*	*Helotiales*	*Helotiaceae*	1	0	OTH	Y	Y	N	Gianoulis TA et al.,2012
Aspergillus aculeatus	*Aspergillus aculeatus* ATCC16872 v1.1	*Ascomycota*	*Eurotiales*	*Aspergillaceae*	1	0	OTH	N	Y	N	de Vries RP et al.,2017
Aspergillus bombycis	*Aspergillus bombycis* NRRL 26010	*Ascomycota*	*Eurotiales*	*Aspergillaceae*	2	0	OTH	N	Y	N	Moore GG et al.,2016
Aspergillus carbonarius	*Aspergillus carbonarius* ITEM 5010 v3	*Ascomycota*	*Eurotiales*	*Aspergillaceae*	1	0	OTH	N	Y	N	de Vries RP et al.,2017
Aspergillus clavatus	*Aspergillus clavatus* NRRL 1 from AspGD	*Ascomycota*	*Eurotiales*	*Aspergillaceae*	1	0	OTH	N	N	N	Arnaud MB et al.,2012
Aspergillus cristatus	*Aspergillus cristatus* GZAAS20.1005	*Ascomycota*	*Eurotiales*	*Aspergillaceae*	2	0	OTH	N	Y	N	Ge Y et al.,2016
Aspergillus flavus	*Aspergillus flavus* NRRL3357	*Ascomycota*	*Eurotiales*	*Aspergillaceae*	2	0	OTH	N	N	N	Arnaud MB et al.,2012
Aspergillus fumigatus	*Aspergillus fumigatus* A1163	*Ascomycota*	*Eurotiales*	*Aspergillaceae*	1	1	OTH	N	N	N	Fedorova et al.,2008
Aspergillus glaucus	*Aspergillus glaucus* v1.0	*Ascomycota*	*Eurotiales*	*Aspergillaceae*	3	1	OTH	N	Y	N	de Vries RP et al.,2017
Aspergillus lentulus	*Aspergillus lentulus*	*Ascomycota*	*Eurotiales*	*Aspergillaceae*	2	0	OTH	N	Y	N	Kusuya et al., 2016
Aspergillus parasiticus	*Aspergillus parasiticus*	*Ascomycota*	*Eurotiales*	*Aspergillaceae*	1	0	OTH	N	N	N	Linz et al., 2014
Aspergillus sydowii	*Aspergillus sydowii* CBS 593.65 v1.0	*Ascomycota*	*Eurotiales*	*Aspergillaceae*	2	0	OTH	N	Y	N	de Vries RP et al.,2017
Aspergillus terreus	*Aspergillus terreus* NIH 2624	*Ascomycota*	*Eurotiales*	*Aspergillaceae*	1	0	OTH	N	Y	N	Arnaud MB et al.,2012
Aspergillus thermomutatus	*Aspergillus thermomutatus*	*Ascomycota*	*Eurotiales*	*Aspergillaceae*	1	0	OTH	N	N	N	Parent-Michaud et al., 2019
Aspergillus udagawae	*Aspergillus udagawae* IFM 46973	*Ascomycota*	*Eurotiales*	*Aspergillaceae*	3	0	OTH	N	Y	N	Kusuya Y et al.,2015
Aspergillus versicolor	*Aspergillus versicolor* v1.0	*Ascomycota*	*Eurotiales*	*Aspergillaceae*	3	0	OTH	N	Y	N	de Vries RP et al.,2017
Aspergillus wentii	*Aspergillus wentii* v1.0	*Ascomycota*	*Eurotiales*	*Aspergillaceae*	1	0	OTH	N	Y	N	de Vries RP et al.,2017
Auricularia auricula-judae	*Auricularia auricula-judae*	*Basidiomycota*	*Auriculariales*	*Auriculariaceae*	6	5	WR	Y	Y	N	This study

Auricularia mesenterica	*Auricularia mesenterica*	*Basidiomycota*	*Auriculariales*	*Auriculariaceae*	6	5	WR	N	N	Y	This study
Auricularia subglabra	*Auricularia subglabra* v2.0	*Basidiomycota*	*Auriculariales*	*Auriculariaceae*	11	4	WR	Y	Y	N	Floudas D et al.,2012
Auriculariopsis ampla	*Auriculariopsis ampla* NL-1724 v1.0	*Basidiomycota*	*Agaricales*	*Schizophyllaceae*	0	0	WR	N	N	N	Almasi E et al.,2019
Baudoinia compniacensis	*Baudoinia compniacensis* UAMH 10762 (4089826) v1.0	*Ascomycota*	*Capnodiales*	*Teratosphaeriaceae*	1	0	OTH	Y	Y	N	Ohm RA et al.,2012
Bjerkandera adusta	*Bjerkandera adusta* v1.0	*Basidiomycota*	*Polyporales*	*Phanerochaetaceae*	14	8	WR	Y	Y	Y	Binder M et al.,2013
Bondarzewia mesenterica	*Bondarzewia mesenterica*	*Basidiomycota*	*Russulales*	*Bondarzewiaceae*	1	0	WR	Y	Y	Y	This study
Botryobasidium botryosum	*Botryobasidium botryosum* v1.0	*Basidiomycota*	*Cantharellales*	*Botryobasidiaceae*	3	0	BR	N	Y	Y	Riley R et al.,2013
Botrytis cinerea	*Botrytis cinerea* v1.0	*Ascomycota*	*Helotiales*	*Sclerotiniaceae*	1	0	OTH	Y	Y	N	Amselem J et al.,2010
Byssochlamys spectabilis	*Byssochlamys spectabilis* No. 5	*Ascomycota*	*Eurotiales*	*Thermoascaceae*	1	0	OTH	Y	Y	N	Oka T et al.,2013
Calocera cornea	*Calocera cornea* v1.0	*Basidiomycota*	*Dacrymycetales*	*Dacrymycetaceae*	0	0	BR	N	N	N	Nagy LG et al.,2016
Calocera viscosa	*Calocera viscosa* v1.0	*Basidiomycota*	*Dacrymycetales*	*Dacrymycetaceae*	0	0	BR	N	N	N	Nagy LG et al.,2016
Cenococcum geophilum	*Cenococcum geophilum* 1.58 v2.0	*Ascomycota*	*Incertae sedis*	*Gloniaceae*	1	0	OTH	Y	Y	N	Peter M et al.,2016
Ceriporiopsis (Gelatoporia)	*Ceriporiopsis (Gelatoporia) subvermispora* B	*Basidiomycota*	*Polyporales*	*Meruliaceae*	0	0	WR	N	N	N	Fernandez-Fueyo et al.,2012
Chondrostereum purpureum	*Chondrostereum purpureum*	*Basidiomycota*	*Agaricales*	*Cyphellaceae*	5	4	WR	Y	Y	Y	This study
Cladophialophora yegresii	*Cladophialophora yegresii* CBS 114405	*Ascomycota*	*Chaetothyriales*	*Herpotrichiellaceae*	1	0	OTH	Y	Y	N	Teixeira MM et al.,2017
Cladosporium fulvum	*Cladosporium fulvum* v1.0	*Ascomycota*	*Capnodiales*	*Davidiellaceae*	1	0	OTH	Y	Y	N	Ohm RA et al.,2011
Cladosporium sphaerospermum	*Cladosporium sphaerospermum* UM 843	*Ascomycota*	*Capnodiales*	*Davidiellaceae*	2	0	OTH	Y	Y	N	Ng KP et al.,2012
Coniophora olivacea	*Coniophora olivacea* MUCL 20566 v1.0	*Basidiomycota*	*Boletales*	*Coniophoraceae*	0	0	BR	N	N	N	Castanera R et al.,2017
Coniophora puteana	*Coniophora puteana* v1.0	*Basidiomycota*	*Boletales*	*Coniophoraceae*	0	0	BR	N	N	Y	Floudas D et al.,2012
Coniosporium apollinis	*Coniosporium apollinis* CBS 100218	*Ascomycota*	*Chaetothyriales*	*Herpotrichiellaceae*	1	1	OTH	Y	Y	N	Teixeira MM et al.,2017
Coprinellus micaceus	*Coprinellus micaceus* FP101781 v2.0	*Basidiomycota*	*Agaricales*	*Psathyrellaceae*	2	0	OTH	Y	Y	Y	Varga T et al.,2019
Coprinopsis cinerea	*Coprinopsis cinerea* AmutBmut pab1-1 v1.0	*Basidiomycota*	*Agaricales*	*Psathyrellaceae*	4	0	OTH	Y	Y	Y	Muraguchi H et al.,2015
Coprinopsis cinerea	*Coprinopsis cinerea*	*Basidiomycota*	*Agaricales*	*Psathyrellaceae*	4	0	OTH	Y	Y	N	Stajich JE et al.,2010
Coprinopsis marcescibilis	*Coprinopsis marcescibilis* CBS121175 v1.0	*Basidiomycota*	*Agaricales*	*Psathyrellaceae*	1	0	OTH	N	N	N	Varga T et al.,2019
Coriolopsis trogii	*Coriolopsis trogii*	*Basidiomycota*	*Agaricales*	*Polyporaceae*	1	0	WR	N	Y	N	Kolwek et al., 2018
Cronartium quercuum	*Cronartium quercuum f. sp. fusiforme* G11 v1.0	*Basidiomycota*	*Pucciniales*	*Cronartiaceae*	3	0	OTH	Y	Y	N	Pendleton AL et al.,2014
Crucibulum laeve	*Crucibulum laeve* CBS 166.37 v1.0	*Basidiomycota*	*Agaricales*	*Nidulariaceae*	3	0	WR	Y	Y	Y	Varga T et al.,2019
Cryptococcus neoformans	*Cryptococcus neoformans var neoformans* JEC21	*Basidiomycota*	*Tremellales*	*Incertae sedis*	0	0	OTH	N	N	N	Loftus BJ et al.,2005
Cryptococcus neoformans	*Cryptococcus neoformans var. grubii* H99	*Basidiomycota*	*Tremellales*	*Incertae sedis*	0	0	OTH	N	N	N	Janbon G et al.,2014
Cylindrobasidium torrendii	*Cylindrobasidium torrendii* FP15055 v1.0	*Basidiomycota*	*Agaricales*	*Physalacriaceae*	0	0	WR	N	N	Y	Floudas D et al.,2015
Cyphellophora europaea	*Cyphellophora europaea* CBS 101466	*Ascomycota*	*Chaetothyriales*	*Herpotrichiellaceae*	1	0	OTH	Y	Y	N	Teixeira MM et al.,2017
Dacryopinax primogenitus	*Dacryopinax primogenitus* DJM 731 SSP1 v1.0	*Basidiomycota*	*Dacrymycetales*	*Dacrymycetaceae*	0	0	BR	N	N	N	Floudas D et al.,2012
Daedalea quercina	*Daedalea quercina* v1.0	*Basidiomycota*	*Polyporales*	*Fomitopsidaceae*	0	0	BR	N	N	Y	Nagy LG et al.,2016
Dendrothele bispora	*Dendrothele bispora* CBS 962.96 v1.0	*Basidiomycota*	*Agaricales*	*Lachnellaceae*	16	5	WR	N	N	N	Varga T et al.,2019

Dentipellis fragilis	*Dentipellis fragilis*	*Basidiomycota*	*Russulales*	*Hericiaceae*	1	0	WR	Y	Y	Y	This study
Dentipellis sp.	*Dentipellis sp.* KUC8613 v1.0	*Basidiomycota*	*Russulales*	*Hericiaceae*	2	0	WR	N	N	N	Park H et al.,2019
Diaporthe ampelina	*Diaporthe ampelina* UCDDA912	*Ascomycota*	*Diaporthales*	*Diaporthaceae*	1	0	OTH	Y	Y	N	Morales-Cruz A et al.,2015
Diaporthe helianthi	*Diaporthe helianthi*	*Ascomycota*	*Diaporthales*	*Diaporthaceae*	1	0	OTH	Y	Y	N	Baroncelli R et al.,2016
Dichomitus squalens	*Dichomitus squalens* CBS464.89 v1.0	*Basidiomycota*	*Polyporales*	*Polyporaceae*	2	0	WR	Y	Y	Y	Casado Lopez S et al.,2019
Dothistroma septosporum	*Dothistroma septosporum* NZE10 v1.0	*Ascomycota*	*Capnodiales*	*Mycosphaerellaceae*	1	0	OTH	Y	Y	N	Ohm RA et al.,2011
Endocarpon pusillum	*Endocarpon pusillum* Z07020	*Ascomycota*	*Verrucariales*	*Verrucariaceae*	4	0	OTH	Y	Y	N	Wang YY et al.,2013
Eurotium rubrum	*Eurotium rubrum* v1.0	*Ascomycota*	*Eurotiales*	*Aspergillaceae*	2	0	OTH	Y	Y	N	Kis-Papo T et al.,2013
Eutypa lata	*Eutypa lata* UCREL1	*Ascomycota*	*Xylariales*	*Diatrypaceae*	1	0	OTH	Y	Y	N	Blanco-Ulate B et al.,2012
Exidia glandulosa	*Exidia glandulosa* v1.0	*Basidiomycota*	*Auriculariales*	*Exidiaceae*	13	10	WR	Y	Y	Y	Nagy LG et al.,2015
Fibroporia radiculosa	*Fibroporia radiculosa* TFFH 294	*Basidiomycota*	*Polyporales*	*Fomitopsidaceae*	1	0	WR	Y	Y	Y	Tang JD et al.,2011
Fibulorhizoctonia sp.	*Fibulorhizoctonia sp.* CBS 109695 v1.0	*Basidiomycota*	*Atheliales*	*Atheliaceae*	2	0	OTH	Y	Y	Y	Nagy LG et al.,2015
Fistulina hepatica	*Fistulina hepatica* v1.0	*Basidiomycota*	*Agaricales*	*Fistulinaceae*	1	0	BR	N	N	Y	Floudas D et al.,2014
Fomes fomentarius	*Fomes fomentarius*	*Basidiomycota*	*Polyporales*	*Polyporaceae*	2	0	WR	Y	N	Y	This study
Fomitiporia mediterranea	*Fomitiporia mediterranea* v1.0	*Basidiomycota*	*Hymenochaetales*	*Hymenochaetaceae*	3	0	WR	Y	Y	Y	Floudas D et al.,2012
Fomitopsis pinicola	*Fomitopsis pinicola* FP-58527 SS1 v3.0	*Basidiomycota*	*Polyporales*	*Fomitopsidaceae*	0	0	BR	N	N	Y	Floudas D et al.,2012
Galerina marginata	*Galerina marginata* v1.0	*Basidiomycota*	*Agaricales*	*Strophariaceae*	5	0	WR	Y	Y	Y	Riley R et al.,2014
Ganoderma sp.	*Ganoderma sp.* 10597 SS1 v1.0	*Basidiomycota*	*Polyporales*	*Ganodermataceae*	3	0	WR	Y	Y	Y	Binder M et al.,2013
Glarea lozoyensis	*Glarea lozoyensis* ATCC 20868	*Ascomycota*	*Helotiales*	*Helotiaceae*	1	0	OTH	N	N	N	Chen L et al.,2013
Gloeophyllum trabeum	*Gloeophyllum trabeum* v1.0	*Basidiomycota*	*Gloeophyllales*	*Gloeophyllaceae*	0	0	BR	N	N	Y	Floudas D et al.,2012
Gymnopus luxurians	*Gymnopus luxurians* v1.0	*Basidiomycota*	*Agaricales*	*Omphalotaceae*	12	7	WR	Y	Y	Y	Kohler A et al.,2015
Hebeloma cylindrosporum	*Hebeloma cylindrosporum* h7 v2.0	*Basidiomycota*	*Agaricales*	*Strophariaceae*	2	0	MYC	Y	Y	Y	Kohler A et al.,2014
Heliocybe sulcata	*Heliocybe sulcata* OMC1185 v1.0	*Basidiomycota*	*Gloeophyllales*	*Gloeophyllaceae*	0	0	BR	N	N	N	Varga T et al.,2019
Heterobasidion annosum	*Heterobasidion annosum* v2.0	*Basidiomycota*	*Russulales*	*Bondarzewiaceae*	1	0	WR	Y	Y	Y	Olson A et al.,2012
Hortaea werneckii	*Hortaea werneckii* EXF-2000 M0 v1.0	*Ascomycota*	*Dothideales*	*Dothideaceae*	2	0	OTH	Y	Y	N	Lenassi M et al.,2013
Hydnomerulius pinastri	*Hydnomerulius pinastri* v2.0	*Basidiomycota*	*Boletales*	*Paxillaceae*	1	0	BR	Y	Y	Y	Kohler A et al.,2015
Hypholoma sublateritium	*Hypholoma sublateritium* v1.0	*Basidiomycota*	*Agaricales*	*Strophariaceae*	1	0	WR	Y	Y	Y	Kohler A et al.,2015
Jaapia argillacea	*Jaapia argillacea* v1.0	*Basidiomycota*	*Jaapiales*	*Jaapiaceae*	1	0	WR	Y	Y	Y	Riley R et al.,2014
Kockovaella imperatae	*Kockovaella imperatae* NRRL Y-17943 v1.0	*Basidiomycota*	*Tremellales*	*Incertae sedis*	0	0	OTH	N	N	N	Mondo SJ et al.,2017
Kretzschmaria deusta	*Kretzschmaria deusta*	*Ascomycota*	*Xylariales*	*Xylariaceae*	1	0	OTH	Y	Y	N	This study
Laccaria amethystina	*Laccaria amethystina* LaAM-08-1 v2.0	*Basidiomycota*	*Agaricales*	*Hydnangiaceae*	2	0	MYC	Y	N	N	Kohler A et al.,2015
Laccaria bicolor	*Laccaria bicolor* v2.0	*Basidiomycota*	*Agaricales*	*Hydnangiaceae*	2	0	MYC	Y	Y	Y	Martin F et al.,2008
Laetiporus sulphureus	*Laetiporus sulphureus* var. *sulphureus* v1.0	*Basidiomycota*	*Polyporales*	*Laetiporaceae*	0	0	BR	N	N	Y	Nagy LG et al.,2016
Lentinula edodes	*Lentinula edodes* W1-26 v1.0	*Basidiomycota*	*Agaricales*	*Omphalotaceae*	2	1	WR	Y	Y	Y	Chen L et al.,2016

Leucoagaricus gongylophorus	*Leucoagaricus gongylophorus* Ac12	*Basidiomycota*	*Agaricales*	*Agaricaceae*	0	0	OTH	N	N	N	Aylward FO et al.,2013
Leucosporidiella creatinivora	*Leucosporidiella creatinivora* 62-1032 v1.0	*Basidiomycota*	*Leucosporidiales*	*Leucosporidiaceae*	0	0	OTH	N	N	N	Mondo SJ et al.,2017
Malassezia globosa	*Malassezia globosa*	*Basidiomycota*	*Malasseziales*	*Malasseziaceae*	0	0	OTH	N	N	N	Xu J et al.,2007
Malassezia sympodialis	*Malassezia sympodialis* ATCC 42132	*Basidiomycota*	*Malasseziales*	*Malasseziaceae*	0	0	OTH	N	N	N	Gioti A et al.,2013
Melampsora lini	*Melampsora lini* CH5	*Basidiomycota*	*Pucciniales*	*Melampsoraceae*	2	0	OTH	Y	Y	N	Nemri A et al.,2014
Metarhizium majus	*Metarhizium majus* ARSEF 297	*Ascomycota*	*Hypocreales*	*Clavicipitaceae*	1	0	OTH	N	N	N	Hu X et al., 2014
Microbotryum lychnidis-dioicae	*Microbotryum lychnidis-dioicae* p1A1 Lamole	*Basidiomycota*	*Microbotryales*	*Microbotryaceae*	0	0	OTH	N	N	N	Perlin MH et al.,2015
Mixia osmundae	*Mixia osmundae* IAM 14324 v1.0	*Basidiomycota*	*Mixiales*	*Mixiaceae*	0	0	OTH	N	N	N	Toome M et al.,2014
Moesziomyces aphidis	*Moesziomyces aphidis* DSM 70725	*Basidiomycota*	*Ustilaginales*	*Ustilaginaceae*	0	0	OTH	N	N	N	Lorenz S et al.,2014
Moniliophthora perniciosa	*Moniliophthora perniciosa* FA553	*Basidiomycota*	*Agaricales*	*Marasmiaceae*	6	5	WR	N	N	N	Mondego JM et al.,2008
Mycena epipterygia	*Mycena epipterygia*	*Basidiomycota*	*Agaricales*	*Mycenaceae*	15	10	WR	Y	Y	Y	This study
Mycosphaerella graminicola	*Mycosphaerella graminicola* v2.0	*Ascomycota*	*Capnodiales*	*Mycosphaerellaceae*	1	0	OTH	N	N	N	Goodwin SB et al.,2011
Neolentinus lepideus	*Neolentinus lepideus* v1.0	*Basidiomycota*	*Gloeophyllales*	*Gloeophyllaceae*	0	0	BR	N	N	Y	Nagy LG et al.,2016
Neosartorya fischeri	*Neosartorya fischeri* NRRL 181	*Ascomycota*	*Eurotiales*	*Trichocomaceae*	2	0	OTH	Y	Y	N	Lonial S et al.,1996
Neurospora crassa	*Neurospora crassa* FGSC 73 trp-3 v1.0	*Ascomycota*	*Sordariales*	*Sordariaceae*	1	0	OTH	Y	Y	N	Baker SE et al.,2015
Neurospora tetrasperma	*Neurospora tetrasperma* FGSC 2508 mat A v2.0	*Ascomycota*	*Sordariales*	*Sordariaceae*	1	0	OTH	Y	Y	N	Ellison CE et al.,2011
Obba rivulosa	*Obba rivulosa* 3A-2 v1.0	*Basidiomycota*	*Polyporales*	*Incertae sedis*	2	0	WR	Y	Y	Y	Miettinen O et al.,2016
Omphalotus olearius	*Omphalotus olearius*	*Basidiomycota*	*Agaricales*	*Omphalotaceae*	1	1	WR	Y	Y	Y	Wawrzyn GT et al.,2012
Paraconiothyrium sporulosum	*Paraconiothyrium sporulosum* AP3s5-JAC2a v1.0	*Ascomycota*	*Pleosporales*	*Montagnulaceae*	1	0	OTH	Y	Y	N	Zeiner CA et al.,2016
Paxillus adelphus	*Paxillus adelphus* Ve08.2h10 v2.0	*Basidiomycota*	*Boletales*	*Paxillaceae*	0	0	MYC	N	N	N	Kohler A et al.,2015
Paxillus involutus	*Paxillus involutus* ATCC 200175 v1.0	*Basidiomycota*	*Boletales*	*Paxillaceae*	0	0	MYC	N	N	Y	Kohler A et al.,2015
Penicillium antarcticum	*Penicillium antarcticum* IBT 31811	*Ascomycota*	*Eurotiales*	*Aspergillaceae*	3	0	OTH	N	Y	N	Nielsen JC et al.,2017
Penicillium chrysogenum	*Penicillium chrysogenum* Wisconsin 54-1255	*Ascomycota*	*Eurotiales*	*Aspergillaceae*	4	0	OTH	N	Y	N	van den Berg MA et al.,2008
Penicillium coprophilum	*Penicillium coprophilum* IBT 31321	*Ascomycota*	*Eurotiales*	*Aspergillaceae*	1	0	OTH	N	Y	N	Nielsen JC et al.,2017
Penicillium decumbens	*Penicillium decumbens* IBT 11843	*Ascomycota*	*Eurotiales*	*Aspergillaceae*	3	0	OTH	N	Y	N	Nielsen JC et al.,2017
Penicillium flavigenum	*Penicillium flavigenum* IBT 14082	*Ascomycota*	*Eurotiales*	*Aspergillaceae*	5	0	OTH	N	Y	N	Nielsen JC et al.,2017
Penicillium griseofulvum	*Penicillium griseofulvum* PG3	*Ascomycota*	*Eurotiales*	*Aspergillaceae*	1	0	OTH	N	Y	N	Banani H et al.,2016
Penicillium nalgiovense	*Penicillium nalgiovense* FM193	*Ascomycota*	*Eurotiales*	*Aspergillaceae*	8	0	OTH	N	Y	N	Nielsen JC et al.,2017
Penicillium nordicum	*Penicillium nordicum* DAOMC 185683	*Ascomycota*	*Eurotiales*	*Aspergillaceae*	5	0	OTH	N	N	N	Wingfield BD et al.,2015
Penicillium polonicum	*Penicillium polonicum* IBT 4502	*Ascomycota*	*Eurotiales*	*Aspergillaceae*	3	0	OTH	N	Y	N	Nielsen JC et al.,2017
Penicillium solitum	*Penicillium solitum* IBT 29525	*Ascomycota*	*Eurotiales*	*Aspergillaceae*	4	0	OTH	N	N	N	Nielsen JC et al.,2017
Penicillium steckii	*Penicillium steckii* IBT 24891	*Ascomycota*	*Eurotiales*	*Aspergillaceae*	2	0	OTH	N	Y	N	Nielsen JC et al.,2017
Penicillium subrubescens	*Penicillium subrubescens* FBCC1632 / CBS132785	*Ascomycota*	*Eurotiales*	*Aspergillaceae*	2	0	OTH	N	Y	N	Peng M et al.,2017
Penicillium thymicola	*Penicillium thymicola* DAOMC 180753 v1.0	*Ascomycota*	*Eurotiales*	*Aspergillaceae*	5	0	OTH	N	Y	N	Nguyen HDT et al.,2016

Penicillium vulpinum	*Penicillium vulpinum* IBT 29486	*Ascomycota*	*Eurotiales*	*Aspergillaceae*	2	0	OTH	N	Y	N	Nielsen JC et al.,2017
Peniophora sp.	*Peniophora sp.* CONTA v1.0	*Basidiomycota*	*Russulales*	*Peniophoraceae*	0	0	WR	N	N	Y	Varga T et al.,2019
Peniophora sp.	*Peniophora sp.* v1.0	*Basidiomycota*	*Russulales*	*Peniophoraceae*	0	0	WR	N	N	N	Nagy LG et al.,2016
Pestalotiopsis clavispora	*Pestalotiopsis clavispora*	*Ascomycota*	*Xylariales*	*Amphisphaeriaceae*	2	0	OTH	Y	Y	N	This study
Phanerochaete carnosa	*Phanerochaete carnosa* HHB-10118-Sp v1.0	*Basidiomycota*	*Polyporales*	*Phanerochaetaceae*	1	0	WR	Y	Y	Y	Suzuki H et al.,2012
Phanerochaete chrysosporium	*Phanerochaete chrysosporium* RP-78 v2.2	*Basidiomycota*	*Polyporales*	*Phanerochaetaceae*	0	0	WR	N	N	N	Ohm RA et al.,2014
Phialocephala scopiformis	*Phialocephala scopiformis* 5WS22E1 v1.0	*Ascomycota*	*Helotiales*	*Vibrisseaceae*	1	0	OTH	Y	Y	N	Walker AK et al.,2016
Phlebia brevispora	*Phlebia brevispora* HHB-7030 SS6 v1.0	*Basidiomycota*	*Corticiales*	*Corticiaceae*	3	2	WR	Y	Y	Y	Binder M et al.,2013
Phlebia centrifuga	*Phlebia centrifuga* FBCC195	*Basidiomycota*	*Corticiales*	*Corticiaceae*	2	2	WR	N	N	N	Makela MR et al.,2018
Phlebia radiata	*Phlebia radiata* isolate 79, FBCC0043	*Basidiomycota*	*Corticiales*	*Corticiaceae*	1	1	WR	N	N	N	Kuuskeri J et al.,2016
Phlebiopsis gigantea	*Phlebiopsis gigantea* v1.0	*Basidiomycota*	*Polyporales*	*Phanerochaetaceae*	5	0	WR	Y	Y	Y	Hori C et al.,2014
Piloderma croceum	*Piloderma croceum* F 1598 v1.0	*Basidiomycota*	*Atheliales*	*Atheliaceae*	4	0	MYC	Y	Y	Y	Kohler A et al.,2015
Piriformospora indica	*Piriformospora indica* DSM 11827 from MPI	*Basidiomycota*	*Sebacinales*	*Sebacinaceae*	2	2	OTH	Y	Y	Y	Zuccaro A et al.,2011
Pisolithus microcarpus	*Pisolithus microcarpus* 441 v1.0	*Basidiomycota*	*Boletales*	*Pisolithaceae*	0	0	MYC	N	N	N	Kohler A et al.,2015
Pisolithus tinctorius	*Pisolithus tinctorius* Marx 270 v1.0	*Basidiomycota*	*Boletales*	*Pisolithaceae*	1	0	MYC	Y	Y	Y	Kohler A et al.,2015
Pleurotus ostreatus	*Pleurotus ostreatus* PC15 v2.0	*Basidiomycota*	*Agaricales*	*Pleurotaceae*	4	3	WR	Y	Y	N	Ruiz-Duenas et al., 2010
Pleurotus ostreatus	*Pleurotus ostreatus* PC9 v1.0	*Basidiomycota*	*Agaricales*	*Pleurotaceae*	4	3	WR	Y	Y	Y	Alfaro M et al.,2016
Plicaturopsis crispa	*Plicaturopsis crispa* v1.0	*Basidiomycota*	*Agaricales*	*Incertae sedis*	0	0	WR	N	N	Y	Kohler A et al.,2015
Pluteus cervinus	*Pluteus cervinus* NL-1719 v1.0	*Basidiomycota*	*Agaricales*	*Pluteaceae*	3	0	WR	N	N	N	Varga T et al.,2019
Polyporus arcularius	*Polyporus arcularius* v1.0	*Basidiomycota*	*Polyporales*	*Polyporaceae*	1	0	WR	N	N	N	Varga T et al.,2019
Polyporus brumalis	*Polyporus brumalis* BRFM 1820 v1.0	*Basidiomycota*	*Polyporales*	*Polyporaceae*	1	0	WR	N	N	N	Miyauchi S et al.,2018
Postia caesia	*Postia caesia*	*Basidiomycota*	*Polyporales*	*Fomitopsidaceae*	2	0	BR	Y	Y	N	This study
Postia placenta	*Postia placenta* MAD-698-R-SB12 v1.0	*Basidiomycota*	*Polyporales*	*Fomitopsidaceae*	2	0	BR	N	N	Y	Gaskell J et al.,2017
Pseudocercospora musae	*Pseudocercospora musae*	*Ascomycota*	*Capnodiales*	*Mycosphaerellaceae*	1	0	OTH	Y	Y	N	Chang et al., 2015
Pseudozyma antarctica	*Pseudozyma antarctica* T-34	*Basidiomycota*	*Ustilaginales*	*Ustilaginaceae*	0	0	OTH	N	N	N	Morita T et al.,2013
Pseudozyma hubeiensis	*Pseudozyma hubeiensis* SY62	*Basidiomycota*	*Ustilaginales*	*Ustilaginaceae*	0	0	OTH	N	N	N	Konishi M et al.,2013
Psilocybe cubensis	*Psilocybe cubensis* v1.0	*Basidiomycota*	*Agaricales*	*Strophariaceae*	2	0	OTH	N	N	N	Fricke J et al.,2017
Psilocybe serbica	*Psilocybe serbica* v1.0	*Basidiomycota*	*Agaricales*	*Strophariaceae*	3	0	OTH	N	N	N	Fricke J et al.,2017
Pterula gracilis	*Pterula gracilis* CBS309.79 v1.0	*Basidiomycota*	*Agaricales*	*Pterulaceae*	1	0	OTH	N	N	N	Varga T et al.,2019
Puccinia coronata	*Puccinia coronata avenae* 12NC29	*Basidiomycota*	*Pucciniales*	*Pucciniaceae*	3	0	OTH	Y	Y	N	Nazareno ES et al.,2018
Puccinia coronata	*Puccinia coronata avenae* 12SD80	*Basidiomycota*	*Pucciniales*	*Pucciniaceae*	3	0	OTH	Y	Y	N	Nazareno ES et al.,2018
Puccinia coronata avenae	*Puccinia coronata avenae*	*Basidiomycota*	*Pucciniales*	*Pucciniaceae*	3	0	OTH	N	Y	N	Nazareno ES et al.,2018
Puccinia graminis	*Puccinia graminis* f. sp. *tritici* v2.0	*Basidiomycota*	*Pucciniales*	*Pucciniaceae*	7	3	OTH	Y	Y	N	Duplessis S et al.,2010
Puccinia striiformis	*Puccinia striiformis* f. sp. *tritici* 104 E137 A-	*Basidiomycota*	*Pucciniales*	*Pucciniaceae*	2	1	OTH	Y	Y	N	Schwessinger B et al.,2018

Puccinia striiformis	*Puccinia striiformis* f. sp. *tritici* PST-130	*Basidiomycota*	*Pucciniales*	*Pucciniaceae*	2	1	OTH	Y	Y	N	Schwessinger B et al.,2018
Puccinia triticina	*Puccinia triticina* 1-1 BBBD Race 1	*Basidiomycota*	*Pucciniales*	*Pucciniaceae*	3	2	OTH	Y	Y	N	Cuomo CA et al.,2017
Punctularia strigosozonata	*Punctularia strigosozonata* v1.0	*Basidiomycota*	*Corticiales*	*Punctulariaceae*	5	4	WR	Y	Y	Y	Floudas D et al.,2012
Pycnoporus cinnabarinus	*Pycnoporus cinnabarinus* BRFM 137	*Basidiomycota*	*Polyporales*	*Polyporaceae*	0	0	WR	N	N	Y	Levasseur A et al.,2014
Resinicium bicolor	*Resinicium bicolor*	*Basidiomycota*	*Hymenochaetales*	*Rickenellaceae*	3	0	WR	Y	N	Y	This study
Rhizoctonia solani	*Rhizoctonia solani* AG-1 IB	*Basidiomycota*	*Cantharellales*	*Ceratobasidiaceae*	6	0	OTH	Y	Y	Y	Wibberg D et al.,2013
Rhizopogon vesiculosus	*Rhizopogon vesiculosus* Smith	*Basidiomycota*	*Boletales*	*Rhizopogonaceae*	1	0	MYC	Y	Y	N	Mujic AB et al.,2017
Rhizopogon vinicolor	*Rhizopogon vinicolor* AM-OR11-026 v1.0	*Basidiomycota*	*Boletales*	*Rhizopogonaceae*	5	0	MYC	Y	Y	N	Mujic AB et al.,2017
Rhodosporidium toruloides	*Rhodosporidium toruloides* IFO0880 v4.0	*Basidiomycota*	*Sporidiobolales*	*Incertae sedis*	0	0	OTH	N	N	N	Coradetti ST et al.,2018
Rhodosporidium toruloides	*Rhodosporidium toruloides* IFO0559_1	*Basidiomycota*	*Sporidiobolales*	*Incertae sedis*	0	0	OTH	N	N	N	Coradetti ST et al.,2018
Rhodosporidium toruloides	*Rhodosporidium toruloides* NP11	*Basidiomycota*	*Sporidiobolales*	*Incertae sedis*	0	0	OTH	N	N	N	Coradetti ST et al.,2018
Rhodotorula graminis	*Rhodotorula graminis* strain WP1 v1.1	*Basidiomycota*	*Sporidiobolales*	*Sporidiobolaceae*	0	0	OTH	N	N	N	Firrincieli A et al.,2015
Rickenella mellea	*Rickenella mellea* v1.0 (SZMC22713)	*Basidiomycota*	*Hymenochaetales*	*Repetobasidiaceae*	3	0	OTH	N	N	N	Krizsan K et al.,2019
Rosellinia necatrix	*Rosellinia necatrix*	*Ascomycota*	*Xylariales*	*Xylariaceae*	1	0	OTH	Y	Y	N	Kanematsu et al., 2014
Schizophyllum commune	*Schizophyllum commune* H4-8 v3.0	*Basidiomycota*	*Agaricales*	*Schizophyllaceae*	0	0	WR	N	N	Y	Ohm RA et al.,2010
Schizopora paradoxa	*Schizopora paradoxa* KUC8140 v1.0	*Basidiomycota*	*Hymenochaetales*	*Schizoporaceae*	4	0	WR	Y	N	Y	Min B et al.,2015
Scleroderma citrinum	*Scleroderma citrinum* Foug A v1.0	*Basidiomycota*	*Boletales*	*Sclerodermataceae*	1	0	MYC	Y	Y	Y	Kohler A et al.,2015
Sclerotinia borealis	*Sclerotinia borealis*	*Ascomycota*	*Helotiales*	*Sclerotiniaceae*	1	0	OTH	Y	Y	N	Mardanov et al., 2014
Sclerotinia sclerotiorum	*Sclerotinia sclerotiorum* v1.0	*Ascomycota*	*Helotiales*	*Sclerotiniaceae*	1	0	OTH	Y	Y	N	Amselem J et al.,2011
Sebacina vermifera	*Sebacina vermifera* MAFF 305830 v1.0	*Basidiomycota*	*Sebacinales*	*Sebacinaceae*	2	0	OTH	N	Y	Y	Kohler A et al.,2015
Septoria musiva	*Septoria musiva* SO2202 v1.0	*Ascomycota*	*Capnodiales*	*Mycosphaerellaceae*	1	0	OTH	Y	Y	N	Ohm RA et al.,2011
Septoria populicola	*Septoria populicola* v1.0	*Ascomycota*	*Capnodiales*	*Mycosphaerellaceae*	1	0	OTH	Y	N	N	Ohm RA et al.,2011
Serpula himantioides	*Serpula himantioides* MUCL38935 v1.0	*Basidiomycota*	*Boletales*	*Serpulaceae*	0	0	BR	N	N	N	Balasundaram SV et al.,2018
Serpula lacrymans	*Serpula lacrymans* S7.9 v2.0	*Basidiomycota*	*Boletales*	*Serpulaceae*	0	0	BR	N	N	Y	Eastwood DC et al.,2011
Serpula lacrymans	*Serpula lacrymans* S7.3 v2.0	*Basidiomycota*	*Boletales*	*Serpulaceae*	0	0	BR	N	N	N	Eastwood DC et al.,2011
Sistotrema brinkmannii	*Sistotrema brinkmannii*	*Basidiomycota*	*Cantharellales*	*Hydnaceae*	2	0	WR	N	N	Y	This study
Sistotremastrum niveocremeum	*Sistotremastrum niveocremeum* HHB9708 ss-1 1.0	*Basidiomycota*	*Trechisporales*	*Trechisporaceae*	0	0	OTH	N	N	N	Nagy LG et al.,2016
Sistotremastrum suecicum	*Sistotremastrum suecicum* v1.0	*Basidiomycota*	*Trechisporales*	*Trechisporaceae*	0	0	OTH	N	N	N	Nagy LG et al.,2016
Sordaria macrospora	*Sordaria macrospora*	*Ascomycota*	*Sordariales*	*Sordariaceae*	2	0	OTH	Y	Y	N	Nowrousian et al., 2010
Sphaerobolus stellatus	*Sphaerobolus stellatus* v1.0	*Basidiomycota*	*Geastrales*	*Sphaerobolaceae*	32	12	WR	N	N	Y	Kohler A et al.,2015
Sporisorium reilianum	*Sporisorium reilianum* SRZ2	*Basidiomycota*	*Ustilaginales*	*Ustilaginaceae*	0	0	OTH	N	N	Y	Schirawski J et al.,2010
Stereum hirsutum	*Stereum hirsutum* FP-91666 SS1 v1.0	*Basidiomycota*	*Russulales*	*Stereaceae*	2	0	WR	N	Y	Y	Floudas D et al.,2012
Suillus brevipes	*Suillus brevipes* Sb2 v2.0	*Basidiomycota*	*Boletales*	*Suillaceae*	1	0	MYC	Y	N	Y	Branco S et al.,2015
Suillus luteus	*Suillus luteus* UH-Slu-Lm8-n1 v3.0	*Basidiomycota*	*Boletales*	*Suillaceae*	1	0	MYC	Y	N	N	Kohler A et al.,2015

Termitomyces albuminosus	*Termitomyces albuminosus*	*Basidiomycota*	*Agaricales*	*Lyophyllaceae*	1	1	OTH	Y	Y	N	Liers et al., 2010
Tilletiaria anomala	*Tilletiaria anomala* UBC 951 v1.0	*Basidiomycota*	*Georgefischeriales*	*Tilletiariaceae*	0	0	OTH	N	N	N	Toome M et al.,2014
Trametes pubescens	*Trametes pubescens* FBCC735	*Basidiomycota*	*Polyporales*	*Polyporaceae*	3	0	WR	Y	Y	Y	Granchi Z et al.,2017
Trametes versicolor	*Trametes versicolor* v1.0	*Basidiomycota*	*Polyporales*	*Polyporaceae*	2	0	WR	Y	Y	N	Floudas D et al.,2012
Tremella encephala	*Naematelia encephela* UCDFST 68-887.2 v1.0	*Basidiomycota*	*Tremellales*	*Tremellaceae*	0	0	OTH	N	N	N	Mondo et al., 2017
Tremella mesenterica	*Tremella mesenterica* Fries v1.0	*Basidiomycota*	*Tremellales*	*Tremellaceae*	0	0	OTH	N	N	N	Floudas D et al.,2012
Trichosporon asahii	*Trichosporon asahii* var. *asahii* CBS 8904	*Basidiomycota*	*Tremellales*	*Trichosporonaceae*	0	0	OTH	N	N	N	Yang RY et al.,2012
Trichosporon asahii	*Trichosporon asahii* var. *asahii* CBS 2479	*Basidiomycota*	*Tremellales*	*Trichosporonaceae*	0	0	OTH	N	N	N	Yang RY et al.,2012
Trichosporon oleaginosus	*Trichosporon oleaginosus* IBC0246 v1.0	*Basidiomycota*	*Tremellales*	*Trichosporonaceae*	0	0	OTH	N	N	N	Kourist R et al.,2015
Tulasnella calospora	*Tulasnella calospora* AL13/4D v1.0	*Basidiomycota*	*Cantharellales*	*Tulasnellaceae*	2	0	OTH	Y	Y	N	Kohler A et al.,2015
Ustilago hordei	*Ustilago hordei* Uh4857_4	*Basidiomycota*	*Ustilaginales*	*Ustilaginaceae*	0	0	OTH	N	N	N	Laurie JD et al.,2012
Ustilago maydis	*Ustilago maydis* 521 v2.0	*Basidiomycota*	*Ustilaginales*	*Ustilaginaceae*	0	0	OTH	N	N	Y	Kamper J et al.,2006
Volvariella volvacea	*Volvariella volvacea* V23	*Basidiomycota*	*Agaricales*	*Pluteaceae*	4	1	WR	N	N	Y	Bao D et al.,2013
Wallemia ichthyophaga	*Wallemia ichthyophaga* EXF-994	*Basidiomycota*	*Wallemiales*	*Wallemiaceae*	0	0	OTH	N	N	N	Zajc J et al.,2013
Wallemia sebi	*Wallemia sebi*	*Basidiomycota*	*Wallemiales*	*Wallemiaceae*	0	0	OTH	N	N	N	Padamsee et al., 2012
Wolfiporia cocos	*Wolfiporia cocos* MD-104 SS10 v1.0	*Basidiomycota*	*Polyporales*	*Incertae sedis*	0	0	BR	N	N	Y	Floudas D et al.,2012
Xylaria hypoxylon	*Xylaria hypoxylon*	*Ascomycota*	*Xylariales*	*Xylariaceae*	1	0	OTH	Y	Y	N	This study
Xylaria longipes	*Xylaria longipes*	*Ascomycota*	*Xylariales*	*Xylariaceae*	2	0	OTH	Y	Y	N	This study
Xylaria polymorfa	*Xylaria polymorfa*	*Ascomycota*	*Xylariales*	*Xylariaceae*	1	0	OTH	Y	Y	N	This study
Zymoseptoria ardabiliae	*Zymoseptoria ardabiliae* STIR04_1.1.1	*Ascomycota*	*Capnodiales*	*Mycosphaerellaceae*	1	0	OTH	N	N	N	Stukenbrock EH et al.,2010

* References of the table are reported at the end of this file

Table S4 – Accession numbers, origins and characteristics of the fungal DyP proteins included in the study. Length refers to the number of amino acids; sign pep, presence of a putative N-terminal signal peptide for secretion; Y and W, number of tyrosine and tryptophan residues; *N*-Glyc, number of predicted *N*-glycosylation sites; Acidic, number of acidic amino acid residues at the putative Mn^{2+} oxidation site; Tree ID, abbreviation of the corresponding protein in the protein phylogenetic tree.

Accession num. (JGI or GenBank)	Organism	Phylum	Length	GRAVY	pI	Sign. pep.	Y	W	*N*-glyc	Acidic	Tree ID	Clade
jgi|Amamu1|133842	*Amanita muscaria* Koide v1.0	*Basidiomycota*	1455	-0.402	6.8	N	4	5	0	57	Amamu1	VI-1
jgi|Amamu1|89285	*Amanita muscaria* Koide v1.0	*Basidiomycota*	1434	-0.312	7.1	N	4	5	1	46	Amamu2	VI-1
jgi|Amath1|68188	*Amanita thiersii* Skay4041 v1.0	*Basidiomycota*	1419	-0.42	6.8	N	5	5	0	55	Amath1	VI-1
jgi|Armga1|1086205	*Armillaria gallica* 21-2 v1.0	*Basidiomycota*	1533	-0.12	5.5	Y	5	4	2	53	Armga1	VI-2
jgi|Armga1|477527	*Armillaria gallica* 21-2 v1.0	*Basidiomycota*	324	-0.182	6.2	N	3	1	0	8	-	-
jgi|Armga1|85486	*Armillaria gallica* 21-2 v1.0	*Basidiomycota*	1191	-0.313	4.7	Y	4	3	1	48	-	-
jgi|Armga1|977077	*Armillaria gallica* 21-2 v1.0	*Basidiomycota*	1473	-0.348	6.2	N	3	4	0	64	Armga2	VI-1
jgi|Armme1_1|1112	*Armillaria mellea* DSM 3731	*Basidiomycota*	1521	-0.234	4.4	Y	6	4	2	58	Armme1	VI-2
jgi|Armme1_1|3330	*Armillaria mellea* DSM 3731	*Basidiomycota*	1470	-0.125	6	Y	6	2	1	48	-	-
jgi|Armme1_1|3547	*Armillaria mellea* DSM 3731	*Basidiomycota*	1467	-0.342	5.4	N	6	3	1	65	Armme1	VI-1
jgi|Armme1_1|5556	*Armillaria mellea* DSM 3731	*Basidiomycota*	1560	-0.293	5.9	N	4	4	0	67	Armme1	VI-1
jgi|Armosto1|263432	*Armillaria ostoyae* C18/9	*Basidiomycota*	3876	-0.028	7.5	N	25	18	0	115	Armos1	VI-2
jgi|Armosto1|266051	*Armillaria ostoyae* C18/9	*Basidiomycota*	1476	-0.337	6	N	4	4	0	64	Armos2	VI-1
jgi|Armosto1|271631	*Armillaria ostoyae* C18/9	*Basidiomycota*	1047	-0.314	6	N	8	9	0	37	-	-
jgi|Armosto1|271634	*Armillaria ostoyae* C18/9	*Basidiomycota*	1533	-0.106	5.9	Y	5	4	2	51	Armos3	VI-2
jgi|Armost1|1026769	*Armillaria solidipes* 28-4 v1.0	*Basidiomycota*	1482	-0.317	5.5	N	4	4	1	66	Armso1	VI-1
jgi|Armost1|1027462	*Armillaria solidipes* 28-4 v1.0	*Basidiomycota*	1533	-0.091	5.5	Y	5	4	1	52	Armso2	VI-2
jgi|Armost1|451317	*Armillaria solidipes* 28-4 v1.0	*Basidiomycota*	1521	-0.253	4.4	Y	6	4	2	60	Armso3	VI-2
jgi|Armost1|800972	*Armillaria solidipes* 28-4 v1.0	*Basidiomycota*	456	-0.452	8.1	N	0	2	2	16	-	-
jgi|Ascra1|4790	*Ascochyta rabiei* ArDII	*Ascomycota*	1404	-0.376	7.2	N	5	6	0	48	Ascra1	I
OM386674	*Ascocoryne cylichnium* This study	*Ascomycota*	2799	-0.335	5.2	N	12	17	0	117	Asccy1	VI-1
jgi|Ascsa1|2371	*Ascocoryne sarcoides* NRRL50072	*Ascomycota*	1503	-0.211	6.5	N	4	5	0	54	Ascsa1	VI-1
jgi|Aspac1|26619	*Aspergillus aculeatus* ATCC16872 v1.1	*Ascomycota*	1629	-0.789	5.9	N	14	7	0	91	-	-
jgi|Aspbom1|2205	*Aspergillus bombycis* NRRL 26010	*Ascomycota*	1428	-0.674	8	N	11	8	2	54	-	-
jgi|Aspbom1|9769	*Aspergillus bombycis* NRRL 26010	*Ascomycota*	2283	-0.8	5.8	N	23	14	1	131	-	-
jgi|Aspca3|60793	*Aspergillus carbonarius* ITEM 5010 v3	*Ascomycota*	1338	-0.761	5.4	N	11	6	2	76	-	-
jgi|Aspcl1|1589	*Aspergillus clavatus* NRRL 1 from AspGD	*Ascomycota*	2265	-0.768	6.4	N	17	14	0	121	-	-
jgi|Aspcri1|3941	*Aspergillus cristatus* GZAAS20.1005	*Ascomycota*	1560	-0.807	5.7	N	14	7	1	40	-	-

jgi|Aspcri1|4914	*Aspergillus cristatus* GZAAS20.1005	*Ascomycota*	936	-0.618	9.6	N	7	1	0	34	-	-
jgi|Aspfl1|30156	*Aspergillus flavus* NRRL3357	*Ascomycota*	2256	-0.816	5.4	N	21	14	2	66	-	-
jgi|Aspfl1|33821	*Aspergillus flavus* NRRL3357	*Ascomycota*	1104	-0.598	9.8	N	8	7	1	107	-	-
jgi|Aspfu_A1163_1|108139	*Aspergillus fumigatus* Af293 from AspGD	*Ascomycota*	1026	-0.525	9.3	Y	5	4	0	71	-	-
jgi|Aspgl1|32757	*Aspergillus glaucus* v1.0	*Ascomycota*	1545	-0.492	5	Y	11	15	2	60	-	-
jgi|Aspgl1|61596	*Aspergillus glaucus* v1.0	*Ascomycota*	1737	-0.84	5.2	N	15	6	0	91	-	-
jgi|Aspgl1|83659	*Aspergillus glaucus* v1.0	*Ascomycota*	1389	-0.681	6.4	N	7	6	0	1	-	-
GAQ05944.1	*Aspergillus lentulus*	*Ascomycota*	1440	-0.597	7.9	N	10	6	2	0	-	-
GAQ11689.1	*Aspergillus lentulus*	*Ascomycota*	1644	-0.837	6.4	N	13	8	1	2	-	-
EIT82921.1	*Aspergillus oryzae* RIB40	*Ascomycota*	1425	-0.646	7.2	N	11	8	2	0	Aspor1	IV
XP_001825715.2	*Aspergillus oryzae* RIB40	*Ascomycota*	2253	-0.811	5.4	N	21	14	0	1	Aspor2	IV
KJK60833.1	*Aspergillus parasiticus* SU-1	*Ascomycota*	2253	-0.81	5.4	N	21	14	2	1	-	-
jgi|Aspsy1|55945	*Aspergillus sydowii* CBS 593.65 v1.0	*Ascomycota*	1509	-0.647	6.8	N	14	7	1	0	-	-
jgi|Aspsy1|86233	*Aspergillus sydowii* CBS 593.65 v1.0	*Ascomycota*	1473	-0.843	7.5	N	13	11	0	2	-	-
jgi|Aspte1|9822	*Aspergillus terreus* NIH 2624	*Ascomycota*	1422	-0.878	6.3	N	18	8	1	0	-	-
OXS06033.1	*Aspergillus thermomutatus*	*Ascomycota*	2190	-0.866	6.3	N	22	13	1	2	-	-
OXN12252.1	*Aspergillus turcosus*	*Ascomycota*	1644	-0.848	5.6	N	14	8	1	2	-	-
OXN33247.1	*Aspergillus turcosus*	*Ascomycota*	1644	-0.804	6	N	14	8	1	2	-	-
jgi|Aspuda1|3672	*Aspergillus udagawae* IFM 46973	*Ascomycota*	1647	-0.867	5.4	N	13	7	0	2	-	-
jgi|Aspuda1|3715	*Aspergillus udagawae* IFM 46973	*Ascomycota*	1443	-0.629	6.9	N	8	8	2	0	-	-
jgi|Aspuda1|6450	*Aspergillus udagawae* IFM 46973	*Ascomycota*	1539	-0.746	8.4	N	9	8	0	2	-	-
jgi|Aspve1|177810	*Aspergillus versicolor* v1.0	*Ascomycota*	1557	-0.771	6.5	N	9	7	1	1	-	-
jgi|Aspve1|23672	*Aspergillus versicolor* v1.0	*Ascomycota*	1473	-0.771	7.4	N	13	10	0	1	-	-
jgi|Aspve1|88553	*Aspergillus versicolor* v1.0	*Ascomycota*	1629	-0.717	6.3	N	16	10	1	1	-	-
jgi|Aspwe1|166972	*Aspergillus wentii* v1.0	*Ascomycota*	1602	-0.792	5.4	N	18	7	1	0	-	-
OM386675	*Auricularia auricula-judae*	*Basidiomycota*	1320	-0.213	9.4	N	6	5	2	0	Aurau1	VI-2
OM386676	*Auricularia auricula-judae*	*Basidiomycota*	1464	-0.121	5.2	Y	10	6	0	0	Aurau2	VI-2
OM386677	*Auricularia auricula-judae*	*Basidiomycota*	1449	0.042	5.4	Y	2	3	1	0	Aurau3	VI-2
OM386678	*Auricularia auricula-judae*	*Basidiomycota*	1299	-0.118	7.3	N	6	5	2	0	Aurau4	VI-2
OM386679	*Auricularia auricula-judae*	*Basidiomycota*	1497	-0.122	4.7	Y	8	4	2	0	Aurau5	VI-2
Auraj|g9546.t1_CHAR|	*Auricularia auricula-judae*	*Basidiomycota*	1527	0.014	6	Y	7	4	0	0	Aurau6	VI-2
OM386680	*Auricularia mesenterica*	*Basidiomycota*	675	-0.018	9.1	Y	6	3	1	0	-	-
OM386681	*Auricularia mesenterica*	*Basidiomycota*	726	0.178	4.3	N	3	2	1	0	-	-
OM386682	*Auricularia mesenterica*	*Basidiomycota*	1518	-0.033	8.4	Y	7	5	2	0	Aurme1	VI-2

OM386683	*Auricularia mesenterica*	*Basidiomycota*	1593	-0.083	5.7	Y	7	6	2	0	Aurme2	VI-2
OM386684	*Auricularia mesenterica*	*Basidiomycota*	1500	0.096	4.6	Y	7	4	5	0	Aurme3	VI-2
OM386685	*Auricularia mesenterica*	*Basidiomycota*	1503	0.054	5.1	Y	2	4	1	0	Aurme4	VI-2
jgi|Aurde3_1|1046076	*Auricularia subglabra* v2.0	*Basidiomycota*	1494	-0.043	7	Y	8	5	0	0	Aurde3	VI-2
jgi|Aurde3_1|1160422	*Auricularia subglabra* v2.0	*Basidiomycota*	1296	-0.164	5.6	N	8	4	3	0	Aurde3	VI-2
jgi|Aurde3_1|1173779	*Auricularia subglabra* v2.0	*Basidiomycota*	1404	-0.077	6.5	N	6	5	2	0	Aurde3	VI-2
jgi|Aurde3_1|1211070	*Auricularia subglabra* v2.0	*Basidiomycota*	1614	0.053	5.4	Y	7	4	1	0	Aurde3	VI-2
jgi|Aurde3_1|1332164	*Auricularia subglabra* v2.0	*Basidiomycota*	1365	-0.206	6.4	N	7	5	1	0	Aurde3	VI-2
jgi|Aurde3_1|1338449	*Auricularia subglabra* v2.0	*Basidiomycota*	1449	-0.155	7.2	N	5	4	0	0	Aurde3	VI-2
jgi|Aurde3_1|1409596	*Auricularia subglabra* v2.0	*Basidiomycota*	1512	-0.076	5.9	Y	6	4	2	0	Aurde3	VI-2
jgi|Aurde3_1|145937	*Auricularia subglabra* v2.0	*Basidiomycota*	1308	-0.083	5.4	N	8	4	0	0	Aurde3	VI-2
jgi|Aurde3_1|173588	*Auricularia subglabra* v2.0	*Basidiomycota*	1596	-0.293	5.4	N	9	4	1	1	Aurde3	VI-2
jgi|Bauco1|571758	*Baudoinia compniacensis* UAMH 10762 (4089826) v1.0	*Ascomycota*	1443	-0.358	7	N	6	6	0	1	Bauco1	I
jgi|Bjead1_1|122562	*Bjerkandera adusta* v1.0	*Basidiomycota*	1494	-0.022	7	Y	12	6	0	0	Bjead1	VI-2
jgi|Bjead1_1|122578	*Bjerkandera adusta* v1.0	*Basidiomycota*	1008	0.058	7.7	Y	7	4	0	0	Bjead1	VI-2
jgi|Bjead1_1|169535	*Bjerkandera adusta* v1.0	*Basidiomycota*	1959	-0.441	7.5	N	23	12	1	1	Bjead1	V
jgi|Bjead1_1|172310	*Bjerkandera adusta* v1.0	*Basidiomycota*	1497	-0.061	5.7	Y	10	5	0	0	Bjead1	VI-2
jgi|Bjead1_1|184870	*Bjerkandera adusta* v1.0	*Basidiomycota*	1440	-0.087	6.6	N	8	5	0	0	Bjead1	VI-2
jgi|Bjead1_1|184878	*Bjerkandera adusta* v1.0	*Basidiomycota*	1500	-0.133	7.6	Y	7	5	2	1	Bjead1	VI-2
jgi|Bjead1_1|28449	*Bjerkandera adusta* v1.0	*Basidiomycota*	423	-0.783	10	N	6	2	1	0	Bjead1	-
jgi|Bjead1_1|287732	*Bjerkandera adusta* v1.0	*Basidiomycota*	1491	-0.166	5	Y	5	5	1	0	Bjead1	VI-2
jgi|Bjead1_1|371576	*Bjerkandera adusta* v1.0	*Basidiomycota*	1920	-0.47	7.1	N	20	9	0	0	Bjead1	III
jgi|Bjead1_1|37610	*Bjerkandera adusta* v1.0	*Basidiomycota*	2421	-0.689	6.4	N	18	16	1	0	Bjead1	III
jgi|Bjead1_1|381455	*Bjerkandera adusta* v1.0	*Basidiomycota*	504	-0.266	5.6	N	6	1	0	0	Bjead1	-
jgi|Bjead1_1|457625	*Bjerkandera adusta* v1.0	*Basidiomycota*	1494	-0.174	5.1	Y	7	5	1	0	Bjead1	VI-2
jgi|Bjead1_1|72253	*Bjerkandera adusta* v1.0	*Basidiomycota*	1497	-0.156	4.6	Y	6	5	3	1	Bjead1	VI-2
jgi|Bjead1_1|72268	*Bjerkandera adusta* v1.0	*Basidiomycota*	1488	-0.089	5.6	Y	8	4	2	0	Bjead1	VI-2
THH17068	*Bondarzewia mesenterica*	*Basidiomycota*	1491	-0.48	7.3	N	5	4	0	3	Bonme1	VI-1
jgi|Botbo1|106029	*Botryobasidium botryosum* v1.0	*Basidiomycota*	1389	-0.3	8.1	N	7	4	1	0	-	-
jgi|Botbo1|147513	*Botryobasidium botryosum* v1.0	*Basidiomycota*	1440	-0.513	6.4	N	9	7	0	0	Botbo1	III
jgi|Botbo1|180290	*Botryobasidium botryosum* v1.0	*Basidiomycota*	1608	-0.437	8.5	N	13	4	1	1	Botbo2	V
CCD33857.1	*Botrytis cinerea* v1.0	*Ascomycota*	1539	-0.762	6.4	N	9	7	0	2	Botci1	IV
jgi|Byssp1|921	*Byssochlamys spectabilis* No. 5	*Ascomycota*	1560	-0.803	7.2	N	10	6	0	2	Byssp1	IV

jgi|Cenge3|669393	*Cenococcum geophilum* 1.58 v2.0	*Ascomycota*	1482	-0.508	6.3	N	7	5	1	2	Cenge1	VI-1
OM386686	*Chondrostereum purpureum*	*Basidiomycota*	1461	-0.157	4.8	Y	6	4	0	0	Chopu1	VI-2
OM386687	*Chondrostereum purpureum*	*Basidiomycota*	1434	-0.416	6.7	N	4	4	0	2	Chopu2	VI-1
OM386688	*Chondrostereum purpureum*	*Basidiomycota*	1512	-0.053	4.5	Y	4	4	1	0	Chopu3	VI-2
OM386689	*Chondrostereum purpureum*	*Basidiomycota*	1512	-0.153	4.5	Y	4	4	1	0	Chopu4	VI-2
OM386690	*Chondrostereum purpureum*	*Basidiomycota*	1512	-0.129	4	Y	7	4	1	1	Chopu5	VI-2
jgi|Claye1|2521	*Cladophialophora yegresii* CBS 114405	*Ascomycota*	1446	-0.406	6.8	N	9	5	1	0	Claye1	I
jgi|Clafu1|193965	*Cladosporium fulvum v*1.0	*Ascomycota*	1530	-0.404	5.3	N	5	6	0	1	Clafu1	I
jgi|Clasph1|2468	*Cladosporium sphaerospermum* UM 843	*Ascomycota*	1440	-0.383	7.6	N	3	6	0	1	Clasp1	I
jgi|Clasph1|4947	*Cladosporium sphaerospermum* UM 843	*Ascomycota*	1539	-0.732	6.9	N	10	7	1	1	Clasp2	IV
jgi|Conap1|99894	*Coniosporium apollinis* CBS 100218	*Ascomycota*	1569	-0.374	6.2	Y	4	6	1	0	Conap1	I
Copmi|g12295.t1|	*Coprinellus micaceus*	*Basidiomycota*	1476	-0.535	7.3	N	3	5	0	2	Copmi1	VI-1
Copmi|g8426.t1|	*Coprinellus micaceus*	*Basidiomycota*	1389	-0.594	5.2	N	6	6	0	2	Copmi2	VI-1
jgi|Copci_AmutBmut1|407604	*Coprinopsis cinerea*	*Basidiomycota*	1482	-0.559	6.3	N	6	4	0	2	Copci1	VI-1
jgi|Copci_AmutBmut1|441320	*Coprinopsis cinerea*	*Basidiomycota*	1947	-0.596	6	N	23	8	0	0	Copci2	III
jgi|Copci1|16393	*Coprinopsis cinerea*	*Basidiomycota*	1317	-0.591	6.5	N	5	4	1	2	Copci3	-
jgi|Copci1|20054	*Coprinopsis cinerea*	*Basidiomycota*	1854	-0.557	6.5	N	23	8	2	0	Copci3	III
jgi|Copci1|2157	*Coprinopsis cinerea*	*Basidiomycota*	1944	-0.601	6.1	N	23	8	2	0	Copci4	III
jgi|Croqu1|655914	*Cronartium quercuum f. sp. fusiforme* G11	*Basidiomycota*	1413	-0.226	7.3	N	7	5	0	0	Croqu1	II
jgi|Croqu1|656486	*Cronartium quercuum f. sp. fusiforme* G11	*Basidiomycota*	1440	-0.233	8	N	10	4	0	1	Croqu2	II
jgi|Croqu1|67268	*Cronartium quercuum f. sp. fusiforme* G11	*Basidiomycota*	1476	-0.368	8.7	N	5	4	1	0	Croqu3	II
jgi|Crula1|631188	*Crucibulum laeve*	*Basidiomycota*	1449	-0.455	6.6	N	6	6	0	2	Crula1	VI-1
jgi|Crula1|651384	*Crucibulum laeve*	*Basidiomycota*	1407	-0.505	6.5	N	5	4	0	2	Crula2	VI-1
jgi|Crula1|689698	*Crucibulum laeve*	*Basidiomycota*	1944	-0.52	6.3	N	26	7	0	0	Crula3	III
jgi|Cypeu1|112934	*Cyphellophora europaea* CBS 101466	*Ascomycota*	1563	-0.699	6.3	N	5	10	0	0	Cypeu1	VI-1
TFY71849	*Dentipellis fragilis*	*Basidiomycota*	1632	-0.266	6.4	N	9	6	0	1	Denfr1	V
jgi|Diaam1|3634	*Diaporthe ampelina* UCDDA912	*Ascomycota*	1431	-0.504	6.1	N	4	5	0	3	Diaam1	VI-1
OCW44408.1	*Diaporthe helianthi*	*Ascomycota*	1431	-0.432	6.9	N	3	5	0	0	Diahe1	VI-1
jgi|Dicsq1|150405	*Dichomitus squalens* LYAD-421 SS1 v1.0	*Basidiomycota*	1452	-0.283	6.1	N	5	5	0	3	Dicsq1	VI-1
jgi|Dotse1|27026	*Dothistroma septosporum* NZE10 v1.0	*Ascomycota*	1527	-0.344	8.6	N	5	6	0	1	Dotse1	I
jgi|EndpusZ1|1894	*Endocarpon pusillum* Z07020	*Ascomycota*	1443	-0.434	6.8	N	8	6	2	0	Endpu1	I
jgi|EndpusZ1|2484	*Endocarpon pusillum* Z07020	*Ascomycota*	1374	-0.318	6.9	N	6	6	1	0	Endpu2	I
jgi|EndpusZ1|7749	*Endocarpon pusillum* Z07020	*Ascomycota*	1569	-0.54	7.1	N	10	7	0	1	Endpu3	I
jgi|EndpusZ1|8716	*Endocarpon pusillum* Z07020	*Ascomycota*	1275	-0.624	6.3	N	5	4	0	3	Endpu4	VI-1

jgi|Eurhe1|452470	*Eurotium rubrum* v1.0	*Ascomycota*	1596	-0.916	6.5	N	18	8	0	0	Eurru1	IV
jgi|Eurhe1|525910	*Eurotium rubrum* v1.0	*Ascomycota*	1710	-0.863	6.2	N	13	10	1	0	Eurru2	IV
jgi|Eutla1|8775	*Eutypa lata* UCREL1	*Ascomycota*	1476	-0.507	6.8	N	4	5	0	1	Eutla1	VI-1
jgi|Exigl1|618969	*Exidia glandulosa* v1.0	*Basidiomycota*	1401	0.028	4.7	N	2	4	3	0	Exigl1	VI-2
jgi|Exigl1|628493	*Exidia glandulosa* v1.0	*Basidiomycota*	1386	-0.156	4.6	N	6	4	4	0	-	-
jgi|Exigl1|646034	*Exidia glandulosa* v1.0	*Basidiomycota*	1545	-0.138	5.3	Y	5	4	1	0	Exigl2	VI-2
jgi|Exigl1|662601	*Exidia glandulosa* v1.0	*Basidiomycota*	906	0.117	6.4	Y	1	3	1	0	-	-
jgi|Exigl1|677651	*Exidia glandulosa* v1.0	*Basidiomycota*	1539	-0.06	6.9	Y	4	6	2	0	Exigl3	VI-2
jgi|Exigl1|694004	*Exidia glandulosa* v1.0	*Basidiomycota*	771	-0.18	5.7	N	4	3	1	0	-	-
jgi|Exigl1|694247	*Exidia glandulosa* v1.0	*Basidiomycota*	591	-0.258	8.5	N	3	2	1	0	-	-
jgi|Exigl1|714830	*Exidia glandulosa* v1.0	*Basidiomycota*	1503	0.113	5.4	Y	9	5	0	0	Exigl4	VI-2
jgi|Exigl1|724930	*Exidia glandulosa* v1.0	*Basidiomycota*	1542	-0.158	5.5	Y	6	5	1	0	Exigl5	VI-2
jgi|Exigl1|767058	*Exidia glandulosa* v1.0	*Basidiomycota*	1506	0.027	5.6	Y	6	4	1	0	Exigl6	VI-2
jgi|Exigl1|770104	*Exidia glandulosa* v1.0	*Basidiomycota*	1506	-0.088	6.4	Y	7	6	0	0	Exigl7	VI-2
jgi|Exigl1|838806	*Exidia glandulosa* v1.0	*Basidiomycota*	1530	0.09	6.1	Y	7	5	0	0	Exigl8	VI-2
jgi|Exigl1|845450	*Exidia glandulosa* v1.0	*Basidiomycota*	1545	-0.121	5.2	Y	5	4	3	1	Exigl9	VI-2
jgi|Fibra1|2048	*Fibroporia radiculosa* TFFH 294	*Basidiomycota*	1680	-0.424	6.5	N	11	6	2	0	Fibra1	V
jgi|Fibsp1|1047202	*Fibulorhizoctonia sp.* CBS 109695 v1.0	*Basidiomycota*	1470	-0.275	7	N	5	5	2	0	Fibsp1	VI-1
jgi|Fibsp1|967594	*Fibulorhizoctonia sp.* CBS 109695 v1.0	*Basidiomycota*	2103	-0.39	7.8	N	23	8	2	1	Fibsp2	V
jgi|Fishe1|57906	*Fistulina hepatica* v1.0	*Basidiomycota*	915	-0.238	8.7	N	5	6	3	0	-	-
OM386691	*Fomes fomentarius*	*Basidiomycota*	2223	-0.172	6.5	N	11	6	2	1	Fomfo1	VI-1
OM386692	*Fomes fomentarius*	*Basidiomycota*	2829	-0.45	5.9	N	26	10	0	0	Fomfo2	V
jgi|Fomme1|161969	*Fomitiporia mediterranea* v1.0	*Basidiomycota*	1422	-0.344	5.1	N	4	5	2	2	Fomme1	VI-2
jgi|Fomme1|17134	*Fomitiporia mediterranea* v1.0	*Basidiomycota*	1410	-0.386	7.1	N	4	5	1	1	Fomme2	VI-2
jgi|Fomme1|97143	*Fomitiporia mediterranea* v1.0	*Basidiomycota*	1413	-0.165	4.8	N	3	4	0	1	Fomme3	VI-2
XP_007271420.1	*Fomitiporia mediterranea* v1.0	*Basidiomycota*	1413	-0.237	4.8	N	3	5	1	0	Fomme4	VI-2
AUW34346.1	*Coriolopsis trogii*	*Basidiomycota*	1452	-0.28	5.7	N	5	5	1	0	-	VI-2
jgi|Galma1|143869	*Galerina marginata* v1.0	*Basidiomycota*	1533	-0.295	7.7	N	5	5	0	0	Galma1	VI-1
jgi|Galma1|159433	*Galerina marginata* v1.0	*Basidiomycota*	1506	-0.409	6.8	N	5	5	1	3	Galma2	VI-1
jgi|Galma1|208622	*Galerina marginata* v1.0	*Basidiomycota*	1602	-0.448	6.4	N	19	5	0	2	Galma3	V
jgi|Galma1|252241	*Galerina marginata* v1.0	*Basidiomycota*	1461	-0.338	6	N	3	5	0	2	Galma4	VI-1
jgi|Galma1|65104	*Galerina marginata* v1.0	*Basidiomycota*	1500	-0.424	6.9	N	4	5	0	1	Galma5	VI-1
jgi|Gansp1|112748	*Ganoderma sp.* 10597 SS1 v1.0	*Basidiomycota*	1479	-0.219	7.1	N	6	4	0	2	Gansp1	VI-1
jgi|Gansp1|123427	*Ganoderma sp.* 10597 SS1 v1.0	*Basidiomycota*	1464	-0.286	9.7	N	5	5	0	2	Gansp2	VI-1

jgi|Gansp1|167328	*Ganoderma sp.* 10597 SS1 v1.0	*Basidiomycota*	1470	-0.288	7.2	N	5	5	1	3	Gansp3	VI-1
EHL03291.1	*Glarea lozoyensis* ATCC 20868	*Ascomycota*	1359	-0.417	8.1	N	6	9	0	2	Glalo1	VI-1
jgi|Glalo1|7636	*Glarea lozoyensis* 74030	*Ascomycota*	1428	-0.554	6.9	N	7	6	0	3	Glalo2	VI-1
OBZ72595.1	*Grifola frondosa* strain 9006-11	*Basidiomycota*	1521	-0.26	6.5	N	8	5	0	1	Grifr1	VI-1
OBZ77251.1	*Grifola frondosa* strain 9006-11	*Basidiomycota*	2739	-0.325	6.5	N	23	10	0	1	Grifr2	V
jgi|Gymlu1|1024286	*Gymnopus luxurians* v1.0	*Basidiomycota*	1530	-0.176	4.3	Y	7	4	2	3	Gymlu1	VI-2
jgi|Gymlu1|166740	*Gymnopus luxurians* v1.0	*Basidiomycota*	1527	-0.245	6.1	Y	9	4	1	0	Gymlu2	VI-2
jgi|Gymlu1|208034	*Gymnopus luxurians* v1.0	*Basidiomycota*	1611	-0.375	7.9	N	15	6	0	1	Gymlu3	V
jgi|Gymlu1|233483	*Gymnopus luxurians* v1.0	*Basidiomycota*	1713	-0.349	5.2	N	17	6	3	0	Gymlu4	V
jgi|Gymlu1|241961	*Gymnopus luxurians* v1.0	*Basidiomycota*	1551	-0.061	5.9	Y	6	4	3	0	Gymlu5	VI-2
jgi|Gymlu1|241962	*Gymnopus luxurians* v1.0	*Basidiomycota*	1599	-0.132	7	Y	5	4	1	1	Gymlu6	VI-2
jgi|Gymlu1|260319	*Gymnopus luxurians* v1.0	*Basidiomycota*	2055	-0.674	7.6	N	26	6	1	1	Gymlu7	III
jgi|Gymlu1|261090	*Gymnopus luxurians* v1.0	*Basidiomycota*	1509	-0.228	4.4	Y	6	5	3	0	Gymlu8	VI-2
jgi|Gymlu1|41146	*Gymnopus luxurians* v1.0	*Basidiomycota*	1551	-0.163	6.2	Y	5	5	2	0	Gymlu9	VI-2
jgi|Gymlu1|41170	*Gymnopus luxurians* v1.0	*Basidiomycota*	1503	-0.089	4.4	Y	6	4	3	0	Gymlu10	VI-2
jgi|Gymlu1|506542	*Gymnopus luxurians* v1.0	*Basidiomycota*	1578	-0.681	7.9	N	16	4	1	0	Gymlu11	III
jgi|Gymlu1|68574	*Gymnopus luxurians* v1.0	*Basidiomycota*	2721	-0.396	6.3	N	30	9	2	0	Gymlu12	V
jgi|Hebcy2|450072	*Hebeloma cylindrosporum* h7 v2.0	*Basidiomycota*	1464	-0.362	6.5	N	5	4	4	0	Hebcy1	VI-1
jgi|Hebcy2|68035	*Hebeloma cylindrosporum* h7 v2.0	*Basidiomycota*	1506	-0.513	9.2	N	6	5	1	0	Hebcy2	VI-1
jgi|Hetan2|40020	*Heterobasidion annosum* v2.0	*Basidiomycota*	1470	-0.388	7.4	N	6	5	0	1	Hetan1	VI-1
jgi|Horwer1|13489	*Hortaea werneckii* EXF-2000 M0 v1.0	*Ascomycota*	1383	-0.363	8.9	N	3	6	0	2	Horwe1	I
jgi|Horwer1|7185	*Hortaea werneckii* EXF-2000 M0 v1.0	*Ascomycota*	1383	-0.341	8.6	N	3	6	0	1	Horwe2	I
jgi|Hydpi2|122607	*Hydnomerulius pinastri* v2.0	*Basidiomycota*	1641	-0.526	6	N	11	6	2	3	Hydpi1	V
jgi|Hypsu1|147511	*Hypholoma sublateritium* v1.0	*Basidiomycota*	1476	-0.353	7.6	N	3	4	2	1	Hypsu1	VI-1
jgi|Hypsu1|47206	*Hypholoma sublateritium* v1.0	*Basidiomycota*	1470	-0.222	6.2	N	4	4	2	1	Hypsu2	VI-1
KYQ38963.1	*Hypsizygus marmoreus* 51987-8	*Basidiomycota*	1944	-0.515	6.2	N	19	8	2	1	Hypma1	III
KYQ39957.1	*Hypsizygus marmoreus* 51987-8	*Basidiomycota*	1455	-0.351	7.7	N	5	4	0	2	Hypma2	VI-1
AZJ17936.1	*Irpex lacteus* F17-CD2	*Basidiomycota*	1545	-0.179	4.5	N	10	6	0	2	-	-
AZJ17937.1	*Irpex lacteus* F17-CD2	*Basidiomycota*	1542	-0.114	6.4	Y	9	5	1	0	-	-
AZJ17935.1	*Irpex lacteus* F17-CD2	*Basidiomycota*	1518	-0.217	6.5	Y	10	5	3	1	-	-
AZJ17934.1	*Irpex lacteus* F17-CD2	*Basidiomycota*	1497	-0.275	4.6	Y	8	6	3	0	-	-
AVJ41190.1	*Irpex lacteus* F17	*Basidiomycota*	1440	-0.355	4.7	N	10	5	1	0	-	-
jgi|Jaaar1|57097	*Jaapia argillacea* v1.0	*Basidiomycota*	1443	-0.327	6.8	N	4	4	2	0	Jaaar1	VI-1
OM386693	*Kretzchmaria deusta*	*Ascomycota*	1518	-0.413	7.4	N	3	5	4	1	Krede1	VI-1

jgi|Lacam2|458683	*Laccaria amethystina* LaAM-08-1 v2.0	*Basidiomycota*	2658	-0.389	5.2	N	25	11	4	1	Lacam1	V
jgi|Lacam2|680685	*Laccaria amethystina* LaAM-08-1 v2.0	*Basidiomycota*	1518	-0.263	8	Y	5	4	0	2	Lacam2	VI-1
jgi|Lacbi2|392869	*Laccaria bicolor* v2.0	*Basidiomycota*	1518	-0.276	6.4	N	5	4	0	1	Lacbi1	VI-1
jgi|Lacbi2|399707	*Laccaria bicolor* v2.0	*Basidiomycota*	1620	-0.391	6.6	N	16	7	1	1	Lacbi2	V
ALL98468.1	*Lentinula edodes*	*Basidiomycota*	1500	-0.09	4.2	Y	5	4	0	1	Lened1	VI-2
jgi|Melli1|203247	*Melampsora lini* CH5	*Basidiomycota*	1494	-0.353	7	N	5	4	0	1	Melli1	II
jgi|Melli1|204152	*Melampsora lini* CH5	*Basidiomycota*	2838	-0.43	8.4	N	9	8	0	1	Melli2	II
jgi|Monpe1_1|82311	*Moniliophthora perniciosa* FA553	*Basidiomycota*	411	0.142	8.5	Y	0	0	5	0	-	-
jgi|Monpe1_1|84765	*Moniliophthora perniciosa* FA553	*Basidiomycota*	408	-0.41	6.8	N	0	2	1	1	-	-
jgi|Monpe1_1|85031	*Moniliophthora perniciosa* FA553	*Basidiomycota*	405	0.259	7.4	Y	0	0	3	0	-	-
jgi|Monpe1_1|86964	*Moniliophthora perniciosa* FA552	*Basidiomycota*	1332	-0.182	6.2	Y	4	4	0	1	-	-
jgi|Monpe1_1|89521	*Moniliophthora perniciosa* FA553	*Basidiomycota*	411	0.38	5.7	Y	0	0	0	0	-	-
jgi|Monpe1_1|92614	*Moniliophthora perniciosa* FA553	*Basidiomycota*	867	-0.428	5.6	N	3	3	0	0	-	-
KTB27642.1	*Moniliophthora roreri* MCA 2997	*Basidiomycota*	1794	-0.126	6.1	Y	8	4	0	0	Monro1	VI-2
KTB34230.1	*Moniliophthora roreri* MCA 2997	*Basidiomycota*	1602	-0.16	5.4	Y	7	4	0	0	Monro2	VI-2
KTB36252.1	*Moniliophthora roreri* MCA 2997	*Basidiomycota*	1575	-0.478	6.6	N	18	5	0	0	Monro3	V
XP_007830391.1	*Moniliophthora roreri* MCA 2997	*Basidiomycota*	1524	-0.48	6.6	N	4	6	1	0	Monro4	VI-1
XP_007845269.1	*Moniliophthora roreri* MCA 2997	*Basidiomycota*	1590	-0.445	5.6	N	18	4	1	0	Monro4	VI-1
XP_007845274.1	*Moniliophthora roreri* MCA 2997	*Basidiomycota*	1599	-0.471	6.9	N	18	5	0	1	Monro4	VI-1
XP_007847605.1	*Moniliophthora roreri* MCA 2997	*Basidiomycota*	1587	-0.166	4.7	Y	4	4	0	1	Monro4	VI-1
XP_007847612.1	*Moniliophthora roreri* MCA 2997	*Basidiomycota*	1362	-0.197	5.2	Y	3	4	0	2	Monro4	VI-1
XP_007847613.1	*Moniliophthora roreri* MCA 2997	*Basidiomycota*	1359	-0.154	5.3	Y	5	3	0	1	Monro4	VI-1
XP_007854757.1	*Moniliophthora roreri* MCA 2997	*Basidiomycota*	1212	-0.278	4.8	N	3	3	0	1	Monro4	VI-1
XP_007859567.1	*Moniliophthora roreri* MCA 2997	*Basidiomycota*	1182	-0.157	6.2	Y	3	3	0	1	Monro4	VI-1
XP_007860005.1	*Moniliophthora roreri* MCA 2997	*Basidiomycota*	1341	-0.192	6	Y	2	4	0	0	Monro4	VI-1
GAT51416.1	*Mycena chlorophos*	*Basidiomycota*	4059	-0.236	5	N	42	31	0	0	Mycch1	VI-2
GAT57708.1	*Mycena chlorophos*	*Basidiomycota*	3564	-0.366	4.9	N	19	19	0	0	Mycch2	VI-2
GAT57709.1	*Mycena chlorophos*	*Basidiomycota*	3303	-0.168	6.5	N	18	11	0	0	Mycch3	VI-2
OM386694	*Mycena epipterygia*	*Basidiomycota*	1395	0.025	8.3	Y	5	4	0	0	Mycep1	VI-2
OM386695	*Mycena epipterygia*	*Basidiomycota*	1326	-0.147	10.1	N	4	4	2	1	Mycep2	VI-2
OM386696	*Mycena epipterygia*	*Basidiomycota*	1194	-0.212	5.9	N	3	6	1	1	-	-
OM386697	*Mycena epipterygia*	*Basidiomycota*	1398	0.046	5	Y	4	4	1	1	-	-
OM386698	*Mycena epipterygia*	*Basidiomycota*	1350	0.103	5	Y	3	4	3	0	Mycep3	VI-2
OM386699	*Mycena epipterygia*	*Basidiomycota*	1347	-0.039	8.5	Y	2	4	2	0	Mycep4	VI-2

OM386700	*Mycena epipterygia*	*Basidiomycota*	1278	-0.067	6.8	Y	2	3	0	1	Mycep5	VI-2
OM386701	*Mycena epipterygia*	*Basidiomycota*	1068	-0.217	10.2	N	3	5	4	0	-	-
OM386702	*Mycena epipterygia*	*Basidiomycota*	1287	-0.066	4.5	Y	5	4	4	0	-	-
OM386703	*Mycena epipterygia*	*Basidiomycota*	1521	0.074	8.7	Y	5	4	2	1	Mycep6	VI-2
Mycep|g30480.t1_CHAR|	*Mycena epipterygia*	*Basidiomycota*	1509	-0.067	4.4	Y	4	5	3	1	Mycep7	VI-2
OM386704	*Mycena epipterygia*	*Basidiomycota*	798	0.051	3.9	N	2	2	1	0	-	-
OM386705	*Mycena epipterygia*	*Basidiomycota*	1392	-0.051	9.8	Y	4	4	0	1	-	-
OM386706	*Mycena epipterygia*	*Basidiomycota*	918	-0.03	4.4	Y	0	2	2	0	-	-
OM386707	*Mycena epipterygia*	*Basidiomycota*	1521	-0.059	6.9	N	8	5	0	0	-	-
B0BK71.1	*Mycetinis scorodonius*	*Basidiomycota*	1539	-0.196	5.1	Y	4	4	0	0	Mycsc1	VI-2
B0BK72.1	*Mycetinis scorodonius*	*Basidiomycota*	1530	-0.152	4.4	Y	5	4	1	0	Mycsc2	VI-2
jgi|Mycgr3|30068	*Mycosphaerella graminicola* v2.0	*Ascomycota*	789	-0.397	6.4	N	1	5	1	0	-	-
jgi|Neofi1|3716	*Neosartorya fischeri* NRRL 181	*Ascomycota*	1647	-0.829	5.6	N	14	8	2	0	Neofi1	IV
jgi|Neofi1|6389	*Neosartorya fischeri* NRRL 181	*Ascomycota*	1443	-0.633	8.3	N	10	6	4	0	Neofi2	IV
jgi|Neucr_trp3_1|1552	*Neurospora crassa* FGSC 73 trp-3 v1.0	*Ascomycota*	1542	-0.728	8.1	N	9	8	1	0	Neucr1	IV
jgi|Neute_matA2|125857	*Neurospora tetrasperma* FGSC 2508 mat A v2.0	*Ascomycota*	1542	-0.742	7.8	N	8	8	1	1	Neute1	IV
jgi|Obbri1|50669	*Obba rivulosa* 3A-2 v1.0	*Basidiomycota*	1455	-0.203	5.8	N	5	5	1	2	Obbri1	VI-1
jgi|Obbri1|819011	*Obba rivulosa* 3A-2 v1.0	*Basidiomycota*	2007	-0.381	5.4	N	20	5	2	0	Obbri2	V
jgi|Ompol1|5621	*Omphalotus olearius*	*Basidiomycota*	1527	-0.102	4.5	Y	5	4	0	1	Ompol1	VI-2
jgi|Parsp1|1183956	*Paraconiothyrium sporulosum* AP3s5-JAC2a v1.0	*Ascomycota*	1509	-0.437	4.7	N	5	5	0	1	Parsp1	VI-1
jgi|Penant1|2254	*Penicillium antarcticum* IBT 31811	*Ascomycota*	1485	-0.579	5.3	N	12	11	0	3	-	-
jgi|Penant1|303	*Penicillium antarcticum* IBT 31811	*Ascomycota*	1410	-0.73	6.3	N	7	7	0	1	-	-
jgi|Penant1|9644	*Penicillium antarcticum* IBT 31811	*Ascomycota*	1608	-0.679	6.4	N	14	10	2	0	-	-
jgi|PenchWisc1_1|137222	*Penicillium chrysogenum* Wisconsin 54-1255	*Ascomycota*	1686	-0.73	8.1	N	13	12	1	1	-	-
jgi|PenchWisc1_1|146662	*Penicillium chrysogenum* Wisconsin 54-1255	*Ascomycota*	1455	-0.55	5.9	N	7	13	1	1	-	-
jgi|PenchWisc1_1|147116	*Penicillium chrysogenum* Wisconsin 54-1255	*Ascomycota*	1599	-0.894	5.8	N	17	9	0	1	-	-
jgi|PenchWisc1_1|148935	*Penicillium chrysogenum* Wisconsin 54-1255	*Ascomycota*	1569	-0.639	6.6	N	14	11	0	1	-	-
jgi|Pencop1|6403	*Penicillium coprophilum* IBT 31321	*Ascomycota*	1581	-0.662	6.3	N	9	7	0	1	-	-
jgi|Pendec1|4654	*Penicillium decumbens* IBT 11843	*Ascomycota*	1542	-0.822	8	N	10	6	1	1	-	-
jgi|Pendec1|5191	*Penicillium decumbens* IBT 11843	*Ascomycota*	1593	-0.622	6.8	N	13	6	0	1	-	-

jgi|Pendec1|6065	*Penicillium decumbens* IBT 11843	*Ascomycota*	1596	-0.895	6.4	N	19	8	1	1	-	-
jgi|Penfla1|1455	*Penicillium flavigenum* IBT 14082	*Ascomycota*	1455	-0.525	6	N	7	13	0	2	-	-
jgi|Penfla1|6774	*Penicillium flavigenum* IBT 14082	*Ascomycota*	1581	-0.724	6.3	N	9	7	0	0	-	-
jgi|Penfla1|7862	*Penicillium flavigenum* IBT 14082	*Ascomycota*	1557	-0.842	5.6	N	15	6	1	1	-	-
jgi|Penfla1|8477	*Penicillium flavigenum* IBT 14082	*Ascomycota*	1590	-0.665	6.8	N	17	10	2	0	-	-
jgi|Penfla1|9909	*Penicillium flavigenum* IBT 14082	*Ascomycota*	1599	-0.893	5.8	N	18	9	0	1	-	-
jgi|Pengri1|7481	*Penicillium griseofulvum* PG3	*Ascomycota*	1581	-0.659	6.6	N	8	7	0	2	-	-
KGO78067.1	*Penicillium italicum*	*Ascomycota*	1575	-0.647	6.4	N	9	7	1	2	Penit1	IV
jgi|Pennal1|2226	*Penicillium nalgiovense* FM193	*Ascomycota*	1656	-0.643	7.4	N	11	7	1	0	-	-
jgi|Pennal1|2538	*Penicillium nalgiovense* FM193	*Ascomycota*	753	-0.72	5.6	N	4	4	1	0	-	-
jgi|Pennal1|2539	*Penicillium nalgiovense* FM193	*Ascomycota*	669	-0.66	9.3	N	3	3	2	0	-	-
jgi|Pennal1|4344	*Penicillium nalgiovense* FM193	*Ascomycota*	1560	-0.81	5.1	N	16	6	2	0	-	-
jgi|Pennal1|4584	*Penicillium nalgiovense* FM193	*Ascomycota*	498	-0.278	4.6	N	4	4	0	2	-	-
jgi|Pennal1|5336	*Penicillium nalgiovense* FM193	*Ascomycota*	1557	-0.735	6.3	N	9	7	1	1	-	-
jgi|Pennal1|5338	*Penicillium nalgiovense* FM193	*Ascomycota*	1572	-0.735	5.7	N	15	12	0	2	-	-
jgi|Pennal1|558	*Penicillium nalgiovense* FM193	*Ascomycota*	1380	-0.71	6.9	N	16	5	1	0	-	-
KOS36077.1	*Penicillium nordicum*	*Ascomycota*	789	-0.773	6.1	N	3	4	0	1	-	-
KOS40929.1	*Penicillium nordicum*	*Ascomycota*	1575	-0.659	6.5	N	10	7	2	0	-	-
KOS43242.1	*Penicillium nordicum*	*Ascomycota*	1590	-0.57	5.3	N	13	10	0	0	-	-
KOS45950.1	*Penicillium nordicum*	*Ascomycota*	1632	-0.581	6.2	N	11	7	0	2	-	-
KOS46351.1	*Penicillium nordicum*	*Ascomycota*	744	-0.654	5.9	N	4	3	0	2	-	-
jgi|Penpol1|2152	*Penicillium polonicum* IBT 4502	*Ascomycota*	1578	-0.659	6.2	N	9	7	0	2	-	-
jgi|Penpol1|5119	*Penicillium polonicum* IBT 4502	*Ascomycota*	1545	-0.814	6.4	N	9	6	1	1	-	-
jgi|Penpol1|9541	*Penicillium polonicum* IBT 4502	*Ascomycota*	1563	-0.645	5.8	N	14	11	1	2	-	-
jgi|Penste1|30	*Penicillium steckii* IBT 24891	*Ascomycota*	1557	-0.807	6.3	N	9	7	1	0	-	-
jgi|Penste1|5653	*Penicillium steckii* IBT 24891	*Ascomycota*	1377	-0.742	8.2	N	16	6	2	0	-	-
jgi|Pensub1|10674	*Penicillium subrubescens* FBCC1632 / CBS132785	*Ascomycota*	1569	-0.765	6.7	N	14	10	0	0	-	-
jgi|Pensub1|10677	*Penicillium subrubescens* FBCC1632 / CBS132785	*Ascomycota*	1164	-0.861	5.1	N	9	6	1	1	-	-
jgi|Penth1|166580	*Penicillium thymicola* DAOMC 180753	*Ascomycota*	1515	-0.461	6.5	N	7	6	0	0	-	-
jgi|Penth1|166618	*Penicillium thymicola* DAOMC 180753	*Ascomycota*	1650	-0.607	6	N	12	7	2	0	-	-
jgi|Penth1|231665	*Penicillium thymicola* DAOMC 180753	*Ascomycota*	1554	-0.632	5.6	N	12	10	0	1	-	-
jgi|Penth1|234339	*Penicillium thymicola* DAOMC 180753	*Ascomycota*	1557	-0.747	6.2	N	9	7	0	2	-	-
jgi|Penth1|242869	*Penicillium thymicola* DAOMC 180753	*Ascomycota*	1578	-0.673	6.6	N	10	7	1	0	-	-

jgi|Penvul1|2753	*Penicillium vulpinum* IBT 29486	*Ascomycota*	1572	-0.688	6.9	N	13	9	1	2	-	-
jgi|Penvul1|3003	*Penicillium vulpinum* IBT 29486	*Ascomycota*	1581	-0.647	6.4	N	8	7	0	0	-	-
KAF7541624	Pestalopsis clavispora	*Ascomycota*	1581	-0.608	9	N	3	5	0	2	Pescl1	VI-1
KAF7540984	*Pestalopsis clavispora*	*Ascomycota*	1530	-0.434	6.2	N	4	6	1	1	Pescl2	VI-1
jgi|Phaca1|249176	*Phanerochaete carnosa* HHB-10118-Sp	*Basidiomycota*	1734	-0.594	5.5	N	14	8	1	0	Phaca1	III
PAV16561.1	*Phellinus noxius* ASM228747 v1	*Basidiomycota*	1407	-0.366	6.1	ND	5	5	0	2	Pheno1	VI-2
jgi|Phisc1|648163	*Phialocephala scopiformis* 5WS22E1 v1.0	*Ascomycota*	1554	-0.396	6.5	N	9	8	1	0	Phisc1	VI-1
CZR60780.1	*Phialocephala subalpina*	*Ascomycota*	1533	-0.39	6.4	N	7	7	0	1	Phisu1	VI-1
XP_017995750.1	*Phialophora attae*	*Ascomycota*	1725	-0.641	5.9	N	7	8	0	1	Phiat1	VI-1
jgi|Phlbr1|150942	*Phlebia brevispora* HHB-7030 SS6 v1.0	*Basidiomycota*	1515	-0.082	4.2	Y	8	7	1	1	Phlbr1	VI-2
jgi|Phlbr1|163671	*Phlebia brevispora* HHB-7030 SS6 v1.0	*Basidiomycota*	1512	-0.064	4.6	Y	11	5	0	1	Phlbr2	VI-2
jgi|Phlbr1|75878	*Phlebia brevispora* HHB-7030 SS6 v1.0	*Basidiomycota*	1425	-0.119	4.4	Y	12	5	0	0	Phlbr3	VI-2
jgi|Phlgi1|125681	*Phlebiopsis gigantea* v1.0	*Basidiomycota*	1593	-0.389	6.9	N	10	9	0	2	Phlgi1	V
jgi|Phlgi1|33977	*Phlebiopsis gigantea* v1.0	*Basidiomycota*	1734	-0.478	6.7	N	17	9	1	1	Phlgi2	V
jgi|Phlgi1|71660	*Phlebiopsis gigantea* v1.0	*Basidiomycota*	1833	-0.417	6.8	N	12	10	2	1	Phlgi3	V
jgi|Phlgi1|78526	*Phlebiopsis gigantea* v1.0	*Basidiomycota*	1329	-0.096	4.8	N	7	8	1	0	-	-
jgi|Phlgi1|85295	*Phlebiopsis gigantea* v1.0	*Basidiomycota*	1578	-0.375	6.6	N	11	8	1	1	Phlgi4	V
jgi|Pilcr1|16772	*Piloderma croceum* F 1598 v1.0	*Basidiomycota*	1398	-0.398	6.8	N	5	4	0	2	Pilcr1	VI-1
jgi|Pilcr1|57880	*Piloderma croceum* F 1598 v1.0	*Basidiomycota*	1398	-0.31	5	N	5	4	0	0	Pilcr2	VI-1
jgi|Pilcr1|810622	*Piloderma croceum* F 1598 v1.0	*Basidiomycota*	1410	-0.309	4.8	N	6	4	0	1	Pilcr3	VI-1
jgi|Pilcr1|816749	*Piloderma croceum* F 1598 v1.0	*Basidiomycota*	1422	-0.344	5.2	N	4	4	0	0	Pilcr4	VI-1
jgi|Pirin1|76752	*Piriformospora indica* DSM 11827 from MPI	*Basidiomycota*	1716	-0.126	6.3	Y	14	9	0	2	Pirin1	VI-2
jgi|Pirin1|76753	*Piriformospora indica* DSM 11827 from MPI	*Basidiomycota*	1710	-0.002	9.3	Y	7	8	1	1	Pirin2	VI-2
jgi|Pisti1|999122	*Pisolithus tinctorius* Marx 270 v1.0	*Basidiomycota*	1590	-0.333	5.4	N	9	3	0	2	Pisti1	V
jgi|PleosPC15_2|52170	*Pleurotus ostreatus* PC15 v2.0	*Basidiomycota*	1440	-0.24	7.5	N	12	8	2	1	Pleos1	VI-2
jgi|PleosPC15_2|62271	*Pleurotus ostreatus* PC15 v2.0	*Basidiomycota*	1551	-0.332	5.6	Y	16	11	1	0	Pleos2	VI-2
ALD10059.1	*Pleurotus ostreatus* PC15 v2.0	*Basidiomycota*	1494	-0.374	5.4	N	15	9	1	0	-	-
ALD10060.1	*Pleurotus ostreatus* PC15 v2.0	*Basidiomycota*	1512	-0.38	6.7	N	5	4	0	1	-	-
CFW94145.1	Pleurotus sapidus	*Basidiomycota*	1548	-0.32	6	Y	16	11	0	2	Plesa1	VI-2
OM386708	*Postia caesia*	*Basidiomycota*	1632	-0.167	9.6	N	5	6	2	0	Posca1	VI-1
OM386709	*Postia caesia*	*Basidiomycota*	885	0.089	4.9	N	4	3	3	1	-	-
jgi|PosplRSB12_1|1159926	*Postia placenta* MAD 698-R v1.0	*Basidiomycota*	2205	-0.495	6.3	N	29	10	2	0	-	-
KXS94273.1	*Pseudocercospora musae*	*Ascomycota*	1527	-0.432	7.1	N	5	7	0	1	Psemu1	I

jgi|PuccoSD80_1|14606	*Puccinia coronata avenae* 12NC29	*Basidiomycota*	1521	-0.518	4.9	N	9	5	0	1	-	II
jgi|PuccoSD80_1|21110	*Puccinia coronata avenae* 12SD80	*Basidiomycota*	1182	-0.62	5.1	N	7	4	0	1	-	II
jgi|Pucgr2|3711	*Puccinia striiformis* f. sp. *tritici* v2.0	*Basidiomycota*	1326	-0.491	5.7	N	5	4	2	0	Pucgr1	II
jgi|Pucgr2|3712	*Puccinia striiformis* f. sp. *tritici* v2.0	*Basidiomycota*	1572	-0.433	5.4	Y	6	6	0	1	Pucgr2	II
jgi|Pucgr2|3713	*Puccinia striiformis* f. sp. *tritici* v2.0	*Basidiomycota*	1737	-0.34	9.4	N	13	6	2	0	Pucgr3	II
jgi|Pucgr2|3714	*Puccinia striiformis* f. sp. *tritici* v2.0	*Basidiomycota*	1557	-0.354	5.4	Y	7	5	0	0	Pucgr4	II
jgi|Pucgr2|3715	*Puccinia striiformis* f. sp. *tritici* v2.0	*Basidiomycota*	1098	-0.363	5.1	Y	9	5	1	1	-	-
jgi|Pucgr2|9831	*Puccinia striiformis* f. sp. *tritici* v2.0	*Basidiomycota*	1488	-0.467	8.9	N	5	5	2	0	Pucgr5	II
jgi|Pucgr2|9855	*Puccinia striiformis* f. sp. *tritici* v2.0	*Basidiomycota*	1488	-0.456	9.1	N	5	5	2	0	Pucgr6	II
jgi|Pucst_PST78_1|5980	*Puccinia striiformis* f. sp. *tritici* PST-130	*Basidiomycota*	1464	-0.44	9.2	N	5	5	3	0	Pucst1	II
jgi|Pucst1|499941	*Puccinia striiformis* f. sp. *tritici* PST-130	*Basidiomycota*	1503	-0.406	9	N	5	5	0	1	Pucst2	II
jgi|Puctr1|2976	*Puccinia triticina* 1-1 BBBD Race 1	*Basidiomycota*	1560	-0.418	5.3	Y	9	6	1	0	Puctr1	II
jgi|Puctr1|5533	*Puccinia triticina* 1-1 BBBD Race 1	*Basidiomycota*	1503	-0.393	6.9	Y	6	5	1	0	Puctr2	II
jgi|Puctr1|5534	*Puccinia triticina* 1-1 BBBD Race 1	*Basidiomycota*	1473	-0.469	6.6	N	5	5	1	0	Puctr3	II
jgi|Punst1|114678	*Punctularia strigosozonata* v1.0	*Basidiomycota*	1518	-0.135	5	Y	16	6	1	4	Punst1	VI-2
jgi|Punst1|120493	*Punctularia strigosozonata* v1.0	*Basidiomycota*	1533	-0.154	4.4	Y	9	5	1	0	Punst2	VI-2
jgi|Punst1|135879	*Punctularia strigosozonata* v1.0	*Basidiomycota*	1527	0.004	4.5	Y	8	6	0	1	Punst3	VI-2
jgi|Punst1|135919	*Punctularia strigosozonata* v1.0	*Basidiomycota*	1485	-0.376	7.9	N	3	7	0	1	Punst4	VI-2
jgi|Punst1|136727	*Punctularia strigosozonata* v1.0	*Basidiomycota*	1524	-0.127	4.6	Y	8	4	2	1	Punst5	VI-2
OQO09220.1	*Rachicladosporium antarcticum* CCFEE 5527	*Ascomycota*	1329	-0.232	5.6	N	3	6	1	2	Racan1	I
OQO26976.1	*Rachicladosporium* sp. CCFEE 5018	*Ascomycota*	1380	-0.23	6	N	3	6	0	1	Racsp1	I
OM386710	*Resinicium bicolor*	*Basidiomycota*	2148	-0.207	7.1	N	9	8	0	1	Resbi1	VI-2
OM386711	*Resinicium bicolor*	*Basidiomycota*	2430	-0.279	6.2	N	8	10	0	0	-	-
OM386712	*Resinicium bicolor*	*Basidiomycota*	1374	-0.062	6.8	N	3	4	1	0	Resbi2	VI-2
jgi|Rhiso1|11100	*Rhizoctonia solani* AG-1 IB	*Basidiomycota*	1551	-0.709	6.2	N	8	8	2	0	Rhiso1	V
jgi|Rhiso1|11347	*Rhizoctonia solani* AG-1 IB	*Basidiomycota*	1413	-0.35	6.3	N	6	6	1	0	Rhiso2	VI-2
jgi|Rhiso1|11648	*Rhizoctonia solani* AG-1 IB	*Basidiomycota*	1443	-0.35	7	N	6	6	1	0	Rhiso3	VI-2
jgi|Rhiso1|13767	*Rhizoctonia solani* AG-1 IB	*Basidiomycota*	1572	-0.722	5	N	12	7	2	0	Rhiso4	V
jgi|Rhiso1|14226	*Rhizoctonia solani* AG-1 IB	*Basidiomycota*	1551	-0.709	7	N	6	8	2	0	Rhiso5	V
jgi|Rhiso1|8461	*Rhizoctonia solani* AG-1 IB	*Basidiomycota*	1539	-0.501	5.8	N	8	8	1	0	Rhiso6	V
jgi|Rhives1|5103	*Rhizopogon vesiculosus* Smith	*Basidiomycota*	1716	-0.48	6.6	N	15	5	1	0	Rhive1	V
jgi|Rhivi1|781585	*Rhizopogon vinicolor* AM-OR11-026 v1.0	*Basidiomycota*	1677	-0.491	6.8	N	15	4	2	0	Rhivi1	V
CZT00265.1	*Rhynchosporium agropyri*	*Ascomycota*	1611	-0.636	8	N	12	7	1	0	Rhyag1	VI-1

CZT01209.1	*Rhynchosporium agropyri*	*Ascomycota*	1479	-0.535	6.3	N	8	5	1	0	Rhyag2	VI-1
CZT53304.1	*Rhynchosporium secalis*	*Ascomycota*	1479	-0.513	6.3	N	8	5	2	0	Rhyse1	VI-1
GAP93476.1	*Rosellinia necatrix* W97	*Ascomycota*	1428	-0.497	8.3	N	5	5	4	0	Rosne1	VI-1
OCB85293.1	*Sanghuangporus baumii*	*Basidiomycota*	1488	-0.391	6.3	N	4	6	3	1	Sanba1	VI-2
jgi|Schpa1|289551	*Schizopora paradoxa* KUC8140 v1.0	*Basidiomycota*	2199	-0.659	6.8	N	29	13	0	1	Schpa1	V
jgi|Schpa1|52072	*Schizopora paradoxa* KUC8140 v1.0	*Basidiomycota*	2232	-0.319	5.3	N	21	8	3	0	-	-
jgi|Schpa1|818443	*Schizopora paradoxa* KUC8140 v1.0	*Basidiomycota*	1431	-0.337	6.5	N	4	5	0	1	Schpa2	VI-2
jgi|Schpa1|974630	*Schizopora paradoxa* KUC8140 v1.0	*Basidiomycota*	1434	-0.23	5.7	N	3	5	0	1	Schpa3	VI-2
jgi|Sclci1|1218585	*Scleroderma citrinum* Foug A v1.0	*Basidiomycota*	1464	-0.266	4.7	N	11	3	3	0	Sclci1	V
ESZ93323.1	*Sclerotinia borealis* F-4128	*Ascomycota*	1581	-0.699	8.1	N	9	8	3	0	Sclbo1	IV
jgi|Sclsc1|7968	*Sclerotinia sclerotiorum* v1.0	*Ascomycota*	1542	-0.77	7.3	N	10	9	2	0	Sclsc1	IV
jgi|Sebvebe1|812493	*Sebacina vermifera* MAFF 305830 v1.0	*Basidiomycota*	1722	-0.081	5.9	Y	9	9	2	0	Sebve1	VI-2
jgi|Sebvebe1|790407	*Sebacina vermifera* MAFF 305830 v1.0	*Basidiomycota*	1452	-0.441	6.5	N	9	5	4	1	Sebve2	VI-2
jgi|Sepmu1|88414	*Septoria musiva* SO2202 v1.0	*Ascomycota*	1452	-0.373	6.2	N	4	7	0	1	Sepmu1	I
jgi|Seppo1|51868	*Septoria populicola* v1.0	*Ascomycota*	2193	-0.472	6.9	N	13	15	0	0	Seppo1	I
OM386713	*Sistotrema brinkmannii*	*Basidiomycota*	1830	-0.237	6.2	N	15	7	2	0	-	-
OM386714	*Sistotrema brinkmannii*	*Basidiomycota*	1569	-0.258	6	N	14	6	0	0	Sisbr1	VI-2
CCC14577.1	*Sordaria macrospora* k-hell	*Ascomycota*	1374	-0.708	6.9	N	13	6	2	1	Sorma1	IV
XP_003347355.1	*Sordaria macrospora* k-hell	*Ascomycota*	1446	-0.707	6.8	N	13	6	2	1	Sorma2	IV
jgi|Sphst1|137411	*Sphaerobolus stellatus* v1.0	*Basidiomycota*	282	-0.074	10.7	N	0	0	1	1	-	-
jgi|Sphst1|163272	*Sphaerobolus stellatus* v1.0	*Basidiomycota*	1377	-0.205	4.9	N	7	4	1	1	-	-
jgi|Sphst1|165527	*Sphaerobolus stellatus* v1.0	*Basidiomycota*	1458	-0.406	6.6	N	13	5	1	1	-	-
jgi|Sphst1|176170	*Sphaerobolus stellatus* v1.0	*Basidiomycota*	900	-0.285	9.4	N	6	3	2	1	-	-
jgi|Sphst1|178598	*Sphaerobolus stellatus* v1.0	*Basidiomycota*	1437	-0.139	4.6	N	5	4	1	0	Sphst1	VI-2
jgi|Sphst1|179814	*Sphaerobolus stellatus* v1.0	*Basidiomycota*	1446	-0.122	4.5	Y	4	5	1	0	Sphst2	VI-2
jgi|Sphst1|179975	*Sphaerobolus stellatus* v1.0	*Basidiomycota*	1551	-0.017	4.6	Y	5	4	0	1	Sphst3	VI-2
jgi|Sphst1|191312	*Sphaerobolus stellatus* v1.0	*Basidiomycota*	1308	-0.018	4.8	N	5	5	2	0	-	-
jgi|Sphst1|194503	*Sphaerobolus stellatus* v1.0	*Basidiomycota*	1011	-0.068	4.9	N	3	5	0	0	-	-
jgi|Sphst1|194507	*Sphaerobolus stellatus* v1.0	*Basidiomycota*	804	-0.362	5.6	N	4	4	0	2	-	-
jgi|Sphst1|226547	*Sphaerobolus stellatus* v1.0	*Basidiomycota*	1416	-0.221	6.9	N	4	4	0	1	Sphst4	VI-1
jgi|Sphst1|245377	*Sphaerobolus stellatus* v1.0	*Basidiomycota*	1443	-0.041	4.4	Y	5	5	1	2	Sphst5	VI-2
jgi|Sphst1|245397	*Sphaerobolus stellatus* v1.0	*Basidiomycota*	1092	-0.056	5.1	Y	4	3	2	0	-	-
jgi|Sphst1|245419	*Sphaerobolus stellatus* v1.0	*Basidiomycota*	1470	-0.071	6.1	Y	4	6	0	1	Sphst6	VI-2
jgi|Sphst1|245420	*Sphaerobolus stellatus* v1.0	*Basidiomycota*	858	-0.49	8.6	N	11	3	1	1	-	-

jgi|Sphst1|248368	*Sphaerobolus stellatus* v1.0	*Basidiomycota*	363	-0.404	4.4	N	3	1	0	1	-	-
jgi|Sphst1|257065	*Sphaerobolus stellatus* v1.0	*Basidiomycota*	492	-0.246	4.2	N	4	1	0	0	-	-
jgi|Sphst1|257082	*Sphaerobolus stellatus* v1.0	*Basidiomycota*	1557	-0.074	4.4	Y	7	4	1	1	Sphst7	VI-2
jgi|Sphst1|258579	*Sphaerobolus stellatus* v1.0	*Basidiomycota*	1458	-0.329	6.2	N	6	5	1	2	Sphst8	VI-2
jgi|Sphst1|261266	*Sphaerobolus stellatus* v1.0	*Basidiomycota*	786	-0.32	6.5	N	3	3	0	2	-	-
jgi|Sphst1|262542	*Sphaerobolus stellatus* v1.0	*Basidiomycota*	846	-0.133	9.8	Y	4	2	1	0	-	-
jgi|Sphst1|263792	*Sphaerobolus stellatus* v1.0	*Basidiomycota*	1551	-0.075	4.2	Y	6	4	2	1	Sphst9	VI-2
jgi|Sphst1|27288	*Sphaerobolus stellatus* v1.0	*Basidiomycota*	1290	-0.289	5.6	N	4	6	1	0	Sphst10	VI-2
jgi|Sphst1|275065	*Sphaerobolus stellatus* v1.0	*Basidiomycota*	810	-0.462	6	N	6	3	0	1	-	-
jgi|Sphst1|29451	*Sphaerobolus stellatus* v1.0	*Basidiomycota*	1512	-0.112	5	Y	5	6	0	1	Sphst11	VI-2
jgi|Sphst1|32449	*Sphaerobolus stellatus* v1.0	*Basidiomycota*	1545	-0.093	4.3	Y	6	4	0	0	Sphst12	VI-2
jgi|Sphst1|35569	*Sphaerobolus stellatus* v1.0	*Basidiomycota*	1788	-0.558	7.9	Y	6	4	2	0	Sphst13	VI-2
jgi|Sphst1|777943	*Sphaerobolus stellatus* v1.0	*Basidiomycota*	1467	-0.259	4.8	N	4	4	2	0	Sphst14	VI-2
jgi|Sphst1|778263	*Sphaerobolus stellatus* v1.0	*Basidiomycota*	936	-0.409	5.4	N	9	3	1	0	-	-
jgi|Sphst1|779978	*Sphaerobolus stellatus* v1.0	*Basidiomycota*	1191	-0.404	5	N	11	4	0	0	Sphst15	VI-2
jgi|Sphst1|780852	*Sphaerobolus stellatus* v1.0	*Basidiomycota*	1557	-0.06	4.4	Y	6	4	2	0	Sphst16	VI-2
jgi|Sphst1|780853	*Sphaerobolus stellatus* v1.0	*Basidiomycota*	1557	-0.034	4.3	Y	6	4	2	2	Sphst17	VI-2
jgi|Stehi1|49405	*Stereum hirsutum* FP-91666 SS1	*Basidiomycota*	1515	-0.376	6.2	N	6	4	2	1	-	-
jgi|Stehi1|94645	*Stereum hirsutum* FP-91666 SS1	*Basidiomycota*	1401	-0.352	5	N	3	4	1	1	Stehi1	VI-1
jgi|Suibr2|955369	*Suillus brevipes* Sb2 v2.0	*Basidiomycota*	2754	-0.35	6.6	N	26	9	1	1	Suibr1	V
jgi|Suilu4|16282	*Suillus luteus* UH-Slu-Lm8-n1 v3.0	*Basidiomycota*	2754	-0.357	6	N	26	9	2	0	Suilu1	V
AAM21606.1	*Termitomyces albuminosus*	*Basidiomycota*	1512	-0.21	4.6	Y	11	5	0	0	Teral1	VI-2
KNZ49722.1	*Termitomyces* sp. J132	*Basidiomycota*	1401	-0.398	8.1	N	5	5	1	0	Tersp1	II
KNZ72538.1	*Termitomyces* sp. J132	*Basidiomycota*	1890	-0.557	5.8	N	25	11	2	0	Tersp2	III
KNZ82347.1	*Termitomyces* sp. J132	*Basidiomycota*	1437	-0.426	6.5	N	4	4	0	1	Tersp3	VI-1
jgi|Trapub1|3942	*Trametes pubescens* FBCC735	*Basidiomycota*	1467	-0.283	5.6	N	6	5	0	1	Trapu1	VI-1
jgi|Trapub1|3943	*Trametes pubescens* FBCC735	*Basidiomycota*	1467	-0.423	6.6	N	8	5	4	0	Trapu2	VI-1
jgi|Trapub1|3947	*Trametes pubescens* FBCC735	*Basidiomycota*	1452	-0.315	5	N	5	5	3	1	Trapu3	VI-1
jgi|Trave1|48870	*Trametes versicolor* v1.0	*Basidiomycota*	1458	-0.272	5.3	N	5	5	1	0	Trave1	VI-1
jgi|Trave1|48874	*Trametes versicolor* v1.0	*Basidiomycota*	1470	-0.228	5.6	N	5	5	3	1	Trave2	VI-1
XP_018666204.1	*Trichoderma gamsii*	*Ascomycota*	621	-0.602	9.6	N	5	2	1	0	-	-
jgi|Tulca1|22166	*Tulasnella calospora* AL13/4D v1.0	*Basidiomycota*	1794	-0.478	7.1	N	8	5	0	0	Tulca1	V
jgi|Tulca1|71445	*Tulasnella calospora* AL13/4D v1.0	*Basidiomycota*	1506	-0.471	5.9	N	7	5	2	1	Tulca2	V
OM674912	Environmental sample	-	1449	-0.441	5.06	N	7	4	1	65	scaffold|3	VI-1

OM674911	Environmental sample	-	1278	-0.058	4.79	N	5	4	3	40	scaffold|2	VI-2
OM674910	Environmental sample	-	1530	-0.149	5.11	Y	5	4	1	50	cluster0	VI-2
OM674909	Environmental sample	-	1518	-0.033	5.88	Y	9	6	5	44	cluster1	VI-2
OM674908	Environmental sample	-	1488	-0.486	6.90	N	6	5	0	67	cluster2	VI-1
OM674907	Environmental sample	-	1476	-0.517	7.01	N	4	6	2	64	cluster3	VI-1
OM674906	Environmental sample	-	1470	-0.306	6.80	N	5	4	1	51	cluster4	VI-1
OM674905	Environmental sample	-	1443	-0.353	6.61	N	3	3	1	58	cluster7	VI-1
OM674904	Environmental sample	-	1506	-0.428	7.15	N	9	7	0	62	cluster8	VI-1
OM674903	Environmental sample	-	1428	-0.449	7.44	N	7	4	1	55	cluster9	VI-1
OM674902	Environmental sample	-	1359	-0.219	7.81	N	3	5	0	50	cluster10	VI-1
OM674901	Environmental sample	-	1506	-0.220	6.97	Y	12	8	1	44	cluster11	VI-2
OM674900	Environmental sample	-	1368	-0.294	5.13	N	3	6	2	61	cluster12	VI-1
OM674899	Environmental sample	-	1263	-0.092	8.25	N	5	4	1	32	cluster13	VI-2
jgi|Volvo1|117411	*Volvariella volvacea* V23	*Basidiomycota*	531	-0.574	5.4	Y	6	6	0	24	-	-
jgi|Volvo1|117412	*Volvariella volvacea* V23	*Basidiomycota*	1437	-0.296	5	N	8	5	0	50	Volvo1	V
jgi|Volvo1|120941	*Volvariella volvacea* V23	*Basidiomycota*	1719	-0.419	7.3	N	7	5	2	76	Volvo2	VI-1
jgi|Volvo1|121121	*Volvariella volvacea* V23	*Basidiomycota*	2013	-0.502	6.3	N	25	7	2	79	Volvo3	III
TGJ80762	*Xylaria hypoxylon*	*Ascomycota*	1485	-0.411	7.1	N	3	5	0	62	Xylhy1	VI-1
RYC54895	*Xylaria longipes*	*Ascomycota*	1416	-0.388	8.1	N	4	5	0	59	Xyllo1	VI-1
RYC65969	*Xylaria longipes*	*Ascomycota*	1488	-0.441	9	N	3	5	4	60	Xyllo2	VI-1
KAH8164444	*Xylaria polymorfa*	*Ascomycota*	1494	-0.382	6.9	N	3	5	3	58	Xylpo1	VI-1
jgi|Zymar1|767462	*Zymoseptoria ardabiliae* STIR04_1.1.1	*Ascomycota*	933	-0.36	5.4	N	1	6	2	40	-	-

Figure S1 – Box plots illustrating, for each of the protein clades, the distributions of the values of the different protein features. Significant differences (Dunn's post-hoc test, $p<0.05$) are reported under each boxplot with letters. The red line corresponds to the average value of the protein feature among the whole set of DyPs.

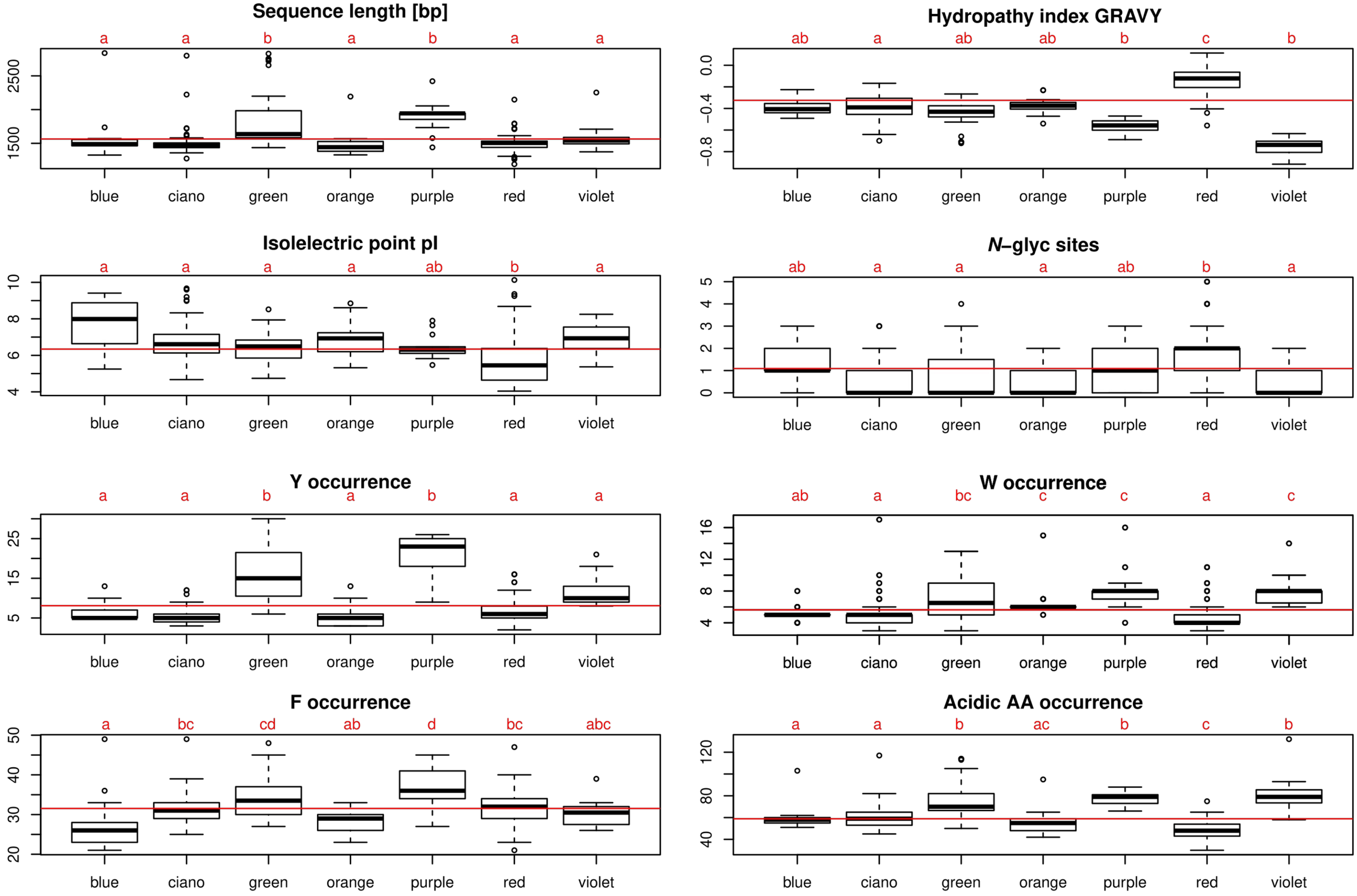

Figure S2 – Most abundant amino acid residues present at positions known or suspected to be essential for catalysis. These amino acids, which are not next to each other in the polypeptide sequences are homologous to the amino acids of the *Auricularia auricula-judae* AauDyPI indicated at the bottom of the figure. Residues variability is visualized using the MEME motif style and separated according to the clade of origin (see Fig. 2).

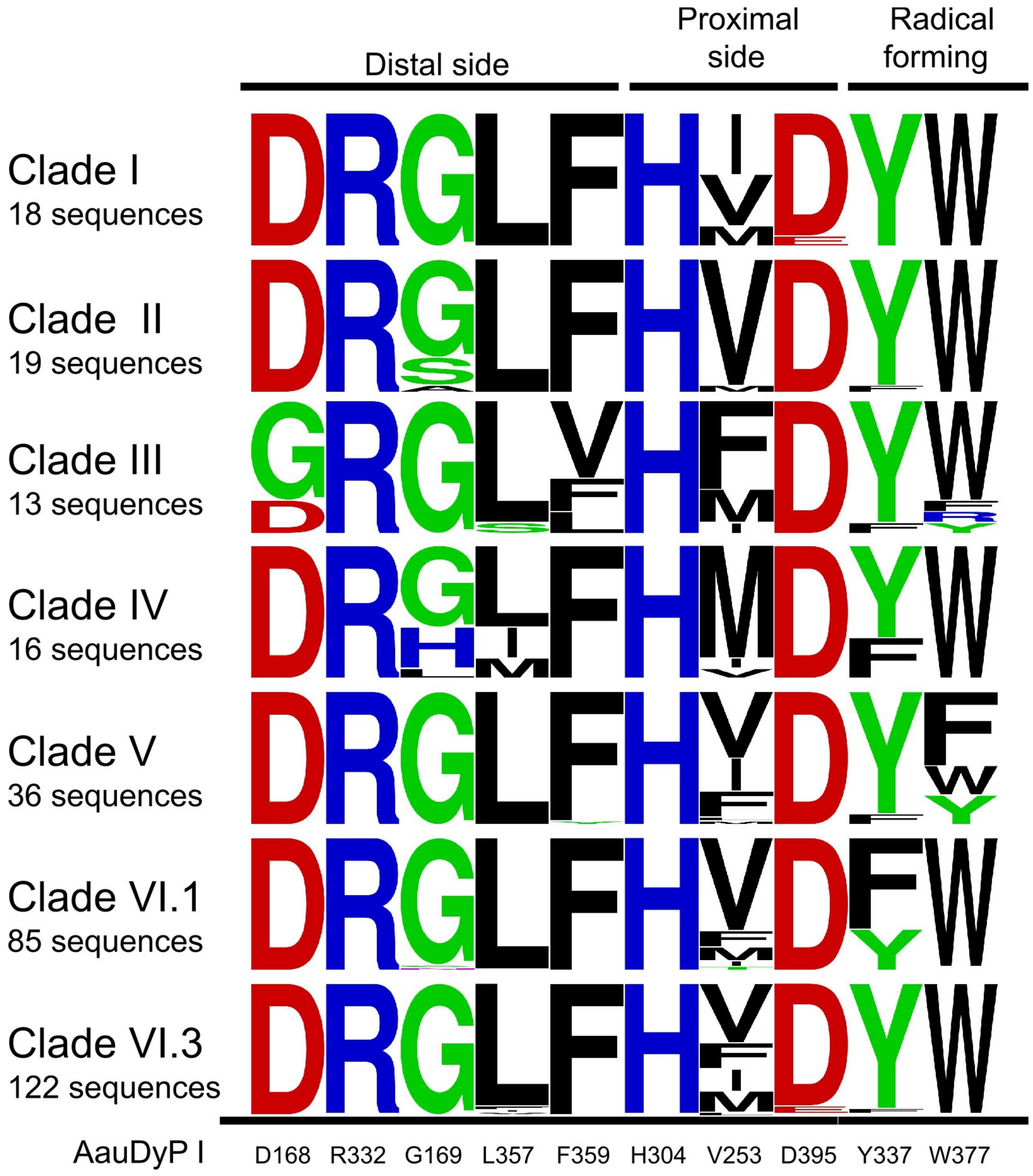

References:

Alfaro M, Castanera R, Lavalin JL, Grigoriev I V, Oguiza JA, Ramìrez L, Pisabarro AG (2016) Comparative and transcriptional analysis of the predicted secretome in the lignocellulose-degrading basidiomycete fungus *Pleurotus ostreatus*. Environ Microbiol 18:4710–4726

Almási É, Sahu N, Krizsán K, Bálint B, Kovács GM, Kiss B, Cseklye J, Drula E, Henrissat B, Nagy I, Chovatia M, Adam C, LaButti K, Lipzen A, Riley R, Grigoriev I V, Nagy LG (2019) Comparative genomics reveals unique wood-decay strategies and fruiting body development in the *Schizophyllaceae*. New Phytol 224:902–915

Amselem J, Cuomo CA, van Kan JAL, Viaud M, Benito EP, Couloux A, Coutinho PM, de Vries RP, Dyer PS, Fillinger S, Fournier E, Gout L, Hahn M, Kohn L, Lapalu N, Plummer KM, Pradier J-M, Quévillon E, Sharon A, Simon A, ten Have A, Tudzynski B, Tudzynski P, Wincker P, Andrew M, Anthouard V, Beever RE, Beffa R, Benoit I, Bouzid O, Brault B, Chen Z, Choquer M, Collémare J, Cotton P, Danchin EG, Da Silva C, Gautier A, Giraud C, Giraud T, Gonzalez C, Grossetete S, Güldener U, Henrissat B, Howlett BJ, Kodira C, Kretschmer M, Lappartient A, Leroch M, Levis C, Mauceli E, Neuvéglise C, Oeser B, Pearson M, Poulain J, Poussereau N, Quesneville H, Rascle C, Schumacher J, Ségurens B, Sexton A, Silva E, Sirven C, Soanes DM, Talbot NJ, Templeton M, Yandava C, Yarden O, Zeng Q, Rollins JA, Lebrun M-H, Dickman M (2011) Genomic Analysis of the Necrotrophic Fungal Pathogens Sclerotinia sclerotiorum and *Botrytis cinerea*. PLoS Genet 7:e1002230

Arnaud MB, Cerqueira GC, Inglis DO, Skrzypek MS, Binkley J, Chibucos MC, Crabtree J, Howarth C, Orvis J, Shah P, Wymore F, Binkley G, Miyasato SR, Simison M, Sherlock G, Wortman JR (2012) The *Aspergillus* Genome Database: recent developments in comprehensive multispecies curation, comparative genomics and community resources. Nucleic Acids Res 40:D653—D658

Aylward FO, Burnum-Johnson KE, Tringe SG, Teiling C, Tremmel DM, Moeller JA, Scott JJ, Barry KW, Piehowski PD, Nicora CD, Malfatti SA, Monroe ME, Purvine SO, Goodwin LA, Smith RD, Weinstock GM, Gerardo NM, Suen G, Lipton MS, Currie CR (2013) *Leucoagaricus gongylophorus* produces diverse enzymes for the degradation of recalcitrant plant polymers in leaf-cutter ant fungus gardens. Appl Environ Microbiol 79:3770–3778

Baker SE, Schackwitz W, Lipzen A, Martin J, Haridas S, LaButti K, Grigoriev I V, Simmons BA, McCluskey K (2015) Draft Genome Sequence of *Neurospora crassa* Strain FGSC 73. Genome Announc 3: e00074-15.

Balasundaram S V, Hess J, Durling MB, Moody SC, Thorbek L, Progida C, LaButti K, Aerts A, Barry K, Grigoriev I V, Boddy L, Högberg N, Kauserud H, Eastwood DC, Skrede I (2018) The fungus that came in from the cold: dry rot’s pre-adapted ability to invade buildings. ISME J 12:791–801

Banani H, Marcet-Houben M, Ballester A-R, Abbruscato P, González-Candelas L, Gabaldón T, Spadaro D (2016) Genome sequencing and secondary metabolism of the postharvest pathogen *Penicillium griseofulvum*. BMC Genomics 17:19

Bao D, Gong M, Zheng H, Chen M, Zhang L, Wang H, Jiang J, Wu L, Zhu Y, Zhu G, Zhou Y, Li C, Wang S, Zhao Y, Zhao G, Tan Q (2013) Sequencing and comparative analysis of the straw mushroom (*Volvariella volvacea*) genome. PLoS One 8: e58294.

Baroncelli R, Scala F, Vergara M, Thon MR, Ruocco M (2016) Draft whole-genome sequence of the *Diaporthe helianthi* 7/96 strain, causal agent of sunflower stem canker. Genom Data 10:151–152

Binder M, Justo A, Riley R, Salamov A, Lopez-Giraldez F, Sjökvist E, Copeland A, Foster B, Sun H, Larsson E, Larsson K-H, Townsend J, Grigoriev I V, Hibbett DS (2013) Phylogenetic and phylogenomic overview of the *Polyporales*. Mycologia 105:1350–1373

Blanco-Ulate B, Rolshausen PE, Cantu D (2013) Draft genome sequence of the grapevine dieback fungus *Eutypa lata* UCR-EL1. Genome Announc 3: e00074-15.

Branco S, Gladieux P, Ellison CE, Kuo A, LaButti K, Lipzen A, Grigoriev I V, Liao H-L, Vilgalys R, Peay KG, Taylor JW, Bruns TD (2015) Genetic isolation between two recently diverged populations of a symbiotic fungus. Mol Ecol 24:2747–2758

Casado López S, Peng M, Daly P, Andreopoulos B, Pangilinan J, Lipzen A, Riley R, Ahrendt S, Ng V, Barry K, Daum C, Grigoriev I V, Hildén KS, Mäkelä MR, de Vries RP (2019) Draft genome sequences of three monokaryotic isolates of the white-rot Basidiomycete fungus *Dichomitus squalens*. Microbiol Resour Announc 8: e00264-19.

Castanera R, Pérez G, López-Varas L, Amselem J, LaButti K, Singan V, Lipzen A, Haridas S, Barry K, Grigoriev I V, Pisabarro AG, Ramirez L (2017) Comparative genomics of *Coniophora olivacea* reveals different patterns of genome expansion in Boletales. BMC Genomics 18:

Chang T-C, Salvucci A, Crous PW, Stergiopoulos I (2016) Comparative genomics of the sigatoka disease complex on banana suggests a link between parallel evolutionary changes in Pseudocercospora fijiensis and Pseudocercospora eumusae and increased virulence on the banana host. PLoS Genet 12:e1005904

Chen L, Yue Q, Zhang X, Xiang M, Wang C, Li S, Che Y, Ortiz-López FJ, Bills GF, Liu X, An Z (2013) Genomics-driven discovery of the pneumocandin biosynthetic gene cluster in the fungus *Glarea lozoyensis*. BMC Genomics 14:339

Chen L, Gong Y, Cai Y, Liu W, Zhou Y, Xiao Y, Xu Z, Liu Y, Lei X, Wang G, Guo M, Ma X, Bian Y (2016) Genome sequence of the edible cultivated mushroom *Lentinula edodes* (shiitake) reveals insights into lignocellulose degradation. PLoS One 11:e0160336

Collins C, Keane TM, Turner DJ, O'Keeffe G, Fitzpatrick DA, Doyle S (2013) Genomic and proteomic dissection of the ubiquitous plant pathogen, *Armillaria mellea*: toward a new infection model system. J Proteome Res 12:2552–2570

Coradetti ST, Pinel D, Geiselman GM, Ito M, Mondo SJ, Reilly MC, Cheng Y-F, Bauer S, Grigoriev I V, Gladden JM, Simmons BA, Brem RB, Arkin AP, Skerker JM (2018) Functional genomics of lipid metabolism in the oleaginous yeast *Rhodosporidium toruloides*. Elife 7:e32110. https://doi.org/10.7554/eLife.32110

Cuomo CA, Bakkeren G, Khalil HB, Panwar V, Joly D, Linning R, Sakthikumar S, Song X, Adiconis X, Fan L, Goldberg JM, Levin JZ, Young S, Zeng Q, Anikster Y, Bruce M, Wang M, Yin C, McCallum B, Szabo LJ, Hulbert S, Chen X, Fellers JP (2017) Comparative analysis highlights variable genome content of wheat rusts and divergence of the mating loci. G3 7:361–376

de Vries RP, Riley R, Wiebenga A, Aguilar-Osorio G, Amillis S, Uchima CA, Anderluh G, Asadollahi M, Askin M, Barry K, Battaglia E, Bayram Ö, Benocci T, Braus-Stromeyer SA, Caldana C, Cánovas D, Cerqueira GC, Chen F, Chen W, Choi C, Clum A, dos Santos RAC, Damásio AR de L, Diallinas G, Emri T, Fekete E, Flipphi M, Freyberg S, Gallo A, Gournas C, Habgood R, Hainaut M, Harispe ML, Henrissat B, Hildén KS, Hope R, Hossain A, Karabika E, Karaffa L, Karányi Z, Kraševec N, Kuo A, Kusch H, LaButti K, Lagendijk EL, Lapidus A, Levasseur A, Lindquist E, Lipzen A, Logrieco AF, MacCabe A, Mäkelä MR, Malavazi I, Melin P, Meyer V, Mielnichuk N, Miskei M, Molnár ÁP, Mulé G, Ngan CY, Orejas M, Orosz E, Ouedraogo JP, Overkamp KM, Park H-S, Perrone G, Piumi F, Punt PJ, Ram AFJ, Ramón A, Rauscher S, Record E, Riaño-Pachón DM, Robert V, Röhrig J, Ruller R, Salamov A, Salih NS, Samson RA, Sándor E, Sanguinetti M, Schütze T, Sepčić K, Shelest E, Sherlock G, Sophianopoulou V, Squina FM, Sun H, Susca A, Todd RB, Tsang A, Unkles SE, van de Wiele N, van Rossen-Uffink D, Oliveira JV de C, Vesth TC, Visser J, Yu J-H, Zhou M, Andersen MR, Archer DB, Baker SE, Benoit I, Brakhage AA, Braus GH, Fischer R, Frisvad JC, Goldman GH, Houbraken J, Oakley B, Pócsi I, Scazzocchio C, Seiboth B, vanKuyk PA, Wortman J, Dyer PS, Grigoriev I V (2017) Comparative genomics reveals high biological diversity and specific adaptations in the industrially and medically important fungal genus *Aspergillus*. Genome Biol 18:

Duplessis S, Cuomo CA, Lin Y-C, Aerts A, Tisserant E, Veneault-Fourrey C, Joly DL, Hacquard S, Amselem J, Cantarel BL, Chiu R, Coutinho PM, Feau N, Field M, Frey P, Gelhaye E, Goldberg J, Grabherr MG, Kodira CD, Kohler A, Kües U, Lindquist EA, Lucas SM, Mago R, Mauceli E, Morin E, Murat C, Pangilinan JL, Park R, Pearson M, Quesneville H, Rouhier N, Sakthikumar S, Salamov AA, Schmutz J, Selles B, Shapiro H, Tanguay P, Tuskan GA, Henrissat B, de Peer Y, Rouzé P, Ellis JG, Dodds PN, Schein JE, Zhong S, Hamelin RC, Grigoriev I V, Szabo LJ, Martin F (2011) Obligate biotrophy features unraveled by the genomic analysis of rust fungi. Proc Natl Acad Sci U S A 108:9166–9171

Eastwood DC, Floudas D, Binder M, Majcherczyk A, Schneider P, Aerts A, Asiegbu FO, Baker SE, Barry K, Bendiksby M, Blumentritt M, Coutinho PM, Cullen D, de Vries RP, Gathman A, Goodell B, Henrissat B, Ihrmark K, Kauserud H, Kohler A, LaButti K, Lapidus A, Lavin JL, Lee Y-H, Lindquist E, Lilly W, Lucas S, Morin E, Murat C, Oguiza JA, Park J, Pisabarro AG, Riley R, Rosling A, Salamov A, Schmidt O, Schmutz J, Skrede I, Stenlid J, Wiebenga A, Xie X, Kües U, Hibbett DS, Hoffmeister D, Högberg N, Martin F, Grigoriev I V, Watkinson SC (2011) The plant cell wall-decomposing machinery underlies the functional diversity of forest fungi. Science (80-) 333:762–765

Ellison CE, Stajich JE, Jacobson DJ, Natvig DO, Lapidus A, Foster B, Aerts A, Riley R, Lindquist EA, Grigoriev I V, Taylor JW (2011) Massive changes in genome architecture accompany the transition to self-fertility in the filamentous fungus *Neurospora tetrasperma*. Genetics 189:55–69

Fedorova ND, Khaldi N, Joardar VS, Maiti R, Amedeo P, Anderson MJ, Crabtree J, Silva JC, Badger JH, Albarraq A, Angiuoli S, Bussey H, Bowyer P, Cotty PJ, Dyer PS, Egan A, Galens K, Fraser-Liggett CM, Haas BJ, Inman JM, Kent R, Lemieux S, Malavazi I, Orvis J, Roemer T, Ronning CM, Sundaram JP, Sutton G, Turner G, Venter JC, White OR, Whitty BR, Youngman P, Wolfe KH, Goldman GH, Wortman JR, Jiang B, Denning DW, Nierman WC (2008) Genomic Islands in the Pathogenic Filamentous Fungus *Aspergillus fumigatus*. PLoS Genet 4:e1000046

Fernández-Fueyo E, Ruiz-Dueñas FJ, Miki Y, Martinez MJ, Hammel KE, Martìnez AT (2012) Lignin-degrading peroxidases from genome of selective ligninolytic fungus *Ceriporiopsis subvermispora*. J Biol Chem 287:16903–16916

Firrincieli A, Otillar R, Salamov A, Schmutz J, Khan Z, Redman RS, Fleck ND, Lindquist E, Grigoriev I V, Doty SL (2015) Genome sequence of the plant growth promoting endophytic yeast *Rhodotorula graminis* WP1. Front Microbiol 6:978

Floudas D, Binder M, Riley R, Barry K, Blanchette RA, Henrissat B, Martinez AT, Otillar R, Spatafora JW, Yadav JS, Aerts A, Benoit I, Boyd A, Carlson A, Copeland A, Coutinho PM, de Vries RP, Ferreira P, Findley K, Foster B, Gaskell J, Glotzer D, Górecki Pawełand Heitman J, Hesse C, Hori C, Igarashi K, Jurgens JA, Kallen N, Kersten P, Kohler A, Kües U, Kumar TKA, Kuo A, LaButti K, Larrondo LF, Lindquist E, Ling A, Lombard V, Lucas S, Lundell T, Martin R, McLaughlin DJ, Morgenstern I, Morin E, Murat C, Nagy LG, Nolan M, Ohm RA, Patyshakuliyeva A, Rokas A, Ruiz-Dueñas FJ, Sabat G, Salamov A, Samejima M, Schmutz J, Slot JC, St John F, Stenlid J, Sun H, Sun S, Syed K, Tsang A, Wiebenga A, Young D, Pisabarro A, Eastwood DC, Martin F, Cullen D, Grigoriev I V, Hibbett DS (2012) The Paleozoic origin of enzymatic lignin decomposition reconstructed from 31 fungal genomes. Science 336:1715–1719

Floudas D, Held BW, Riley R, Nagy LG, Koehler G, Ransdell AS, Younus H, Chow J, Chiniquy J, Lipzen A, Tritt A, Sun H, Haridas S, LaButti K, Ohm RA, Kües U, Blanchette RA, Grigoriev I V., Minto RE, Hibbett DS (2015) Evolution of novel wood decay mechanisms in *Agaricales* revealed by the genome sequences of *Fistulina hepatica* and Cylindrobasidium torrendii. Fungal Genet Biol 76:78–92 . https://doi.org/10.1016/j.fgb.2015.02.002

Fricke J, Blei F, Hoffmeister D (2017) Enzymatic synthesis of psilocybin. Angew Chem Int Ed Engl 56:12352–12355

Gaskell J, Kersten P, Larrondo LF, Canessa P, Martinez D, Hibbett D, Schmoll M, Kubicek CP, Martinez AT, Yadav J, Master E, Magnuson JK, Yaver D, Berka R, Lail K, Chen C, LaButti K, Nolan M, Lipzen A, Aerts A, Riley R, Barry K, Henrissat B, Blanchette R, Grigoriev I V, Cullen D (2017) Draft genome sequence of a monokaryotic model brown-rot fungus *Postia* (*Rhodonia*) *placenta* SB12. Genom Data 14:21–23

Ge Y, Wang Y, Liu Y, Tan Y, Ren X, Zhang X, Hyde KD, Liu Y, Liu Z (2016) Comparative genomic and transcriptomic analyses of the Fuzhuan brick tea-fermentation fungus *Aspergillus cristatus*. BMC Genomics 17:428

Gianoulis TA, Griffin MA, Spakowicz DJ, Dunican BF, Alpha CJ, Sboner A, Sismour AM, Kodira C, Egholm M, Church GM, Gerstein MB, Strobel SA (2012) Genomic analysis of the hydrocarbon-producing, cellulolytic, endophytic fungus *Ascocoryne sarcoides*. PLoS Genet 8:e1002558

Gioti A, Nystedt B, Li W, Xu J, Andersson A, Averette AF, Münch K, Wang X, Kappauf C, Kingsbury JM, Kraak B, Walker LA, Johansson HJ, Holm T, Lehtiö J, Stajich JE, Mieczkowski P, Kahmann R, Kennell JC, Cardenas ME, Lundeberg J, Saunders CW, Boekhout T, Dawson TL, Munro CA, de Groot PWJ, Butler G, Heitman J, Scheynius A (2013) Genomic insights into the atopic eczema-associated skin commensal yeast *Malassezia sympodialis*. MBio 4:

Goodwin SB, Ben M'Barek S, Dhillon B, Wittenberg AHJ, Crane CF, Hane JK, Foster AJ, der Lee TAJ, Grimwood J, Aerts A, Antoniw J, Bailey A, Bluhm B, Bowler J, Bristow J, van der Burgt A, Canto-Canché B, Churchill ACL, Conde-Ferràez L, Cools HJ, Coutinho PM, Csukai M, Dehal P, De Wit P, Donzelli B, van de Geest HC, van Ham RCHJ, Hammond-Kosack KE, Henrissat B, Kilian A, Kobayashi AK, Koopmann E, Kourmpetis Y, Kuzniar A, Lindquist E, Lombard V, Maliepaard C, Martins N, Mehrabi R, Nap JPH, Ponomarenko A, Rudd JJ, Salamov A, Schmutz J, Schouten HJ, Shapiro H, Stergiopoulos I, Torriani SFF, Tu H, de Vries RP, Waalwijk C, Ware SB, Wiebenga A, Zwiers L-H, Oliver RP, Grigoriev I V, Kema GHJ (2011) Finished genome of the fungal wheat pathogen *Mycosphaerella graminicola* reveals dispensome structure, chromosome plasticity, and stealth pathogenesis. PLoS Genet 7:e1002070

Granchi Z, Peng M, Chi-A-Woeng T, de Vries RP, Hildén K, Mäkelä MR (2017) Genome Sequence of the Basidiomycete White-Rot Fungus *Trametes pubescens* FBCC735. Genome Announc 5:

Hess J, Skrede I, Wolfe BE, LaButti K, Ohm RA, Grigoriev I V, Pringle A (2014) Transposable element dynamics among asymbiotic and ectomycorrhizal *Amanita* fungi. Genome Biol Evol 6:1564–1578

Hori C, Ishida T, Igarashi K, Samejima M, Suzuki H, Master E, Ferreira P, Ruiz-Dueñas FJ, Held B, Canessa P, Larrondo LF, Schmoll M, Druzhinina IS, Kubicek CP, Gaskell JA, Kersten P, St John F, Glasner J, Sabat G, Splinter BonDurant S, Syed K, Yadav J, Mgbeahuruike AC, Kovalchuk A, Asiegbu FO, Lackner G, Hoffmeister D, Rencoret J, Gutiérrez A, Sun H, Lindquist E, Barry K, Riley R, Grigoriev I V, Henrissat B, Kües U, Berka RM, Martìnez AT, Covert SF, Blanchette RA, Cullen D (2014) Analysis of the *Phlebiopsis gigantea* genome, transcriptome and secretome provides insight into its pioneer colonization strategies of wood. PLoS Genet 10:e1004759

Hu X, Xiao G, Zheng P, Shang Y, Su Y, Zhang X, Liu X, Zhan S, St Leger RJ, Wang C (2014) Trajectory and genomic determinants of fungal-pathogen speciation and host adaptation. Proc Natl Acad Sci USA 111:16796–16801

Janbon G, Ormerod KL, Paulet D, Byrnes EJ, Yadav V, Chatterjee G, Mullapudi N, Hon C-C, Billmyre RB, Brunel F, Bahn Y-S, Chen W, Chen Y, Chow EWL, Coppée J-Y, Floyd-Averette A, Gaillardin C, Gerik KJ, Goldberg J, Gonzalez-Hilarion S, Gujja S, Hamlin JL, Hsueh Y-P, Ianiri G, Jones S, Kodira CD, Kozubowski L, Lam W, Marra M, Mesner LD, Mieczkowski PA, Moyrand F, Nielsen K, Proux C, Rossignol T, Schein JE, Sun S, Wollschlaeger C, Wood IA, Zeng Q, Neuvéglise C, Newlon CS, Perfect JR, Lodge JK, Idnurm A, Stajich JE, Kronstad JW, Sanyal K, Heitman J, Fraser JA, Cuomo CA, Dietrich FS (2014) Analysis of the genome and transcriptome of *Cryptococcus neoformans* var. *grubii* reveals complex RNA expression and microevolution leading to virulence attenuation. PLoS Genet 10:e1004261

Kämper J, Kahmann R, Bölker M, Ma L-J, Brefort T, Saville BJ, Banuett F, Kronstad JW, Gold SE, Müller O, Perlin MH, Wösten HAB, de Vries R, Ruiz-Herrera J, Reynaga-Peña CG, Snetselaar K, McCann M, Pérez-Mart\'\in J, Feldbrügge M, Basse CW, Steinberg G, Ibeas JI, Holloman W, Guzman P, Farman M, Stajich JE, Sentandreu R, González-Prieto JM, Kennell JC, Molina L, Schirawski J, Mendoza-Mendoza A, Greilinger D, Münch K, Rössel N, Scherer M, Vranes M, Ladendorf O, Vincon V, Fuchs U, Sandrock B, Meng S, Ho ECH, Cahill MJ, Boyce KJ, Klose J, Klosterman SJ, Deelstra HJ, Ortiz-Castellanos L, Li W, Sanchez-Alonso P, Schreier PH, Häuser-Hahn I, Vaupel M, Koopmann E, Friedrich G, Voss H, Schlüter T, Margolis J, Platt D, Swimmer C, Gnirke A, Chen F, Vysotskaia V, Mannhaupt G, Güldener U, Münsterkötter M, Haase D, Oesterheld M, Mewes H-W, Mauceli EW, DeCaprio D, Wade CM, Butler J, Young S, Jaffe DB, Calvo S, Nusbaum C, Galagan J, Birren BW (2006) Insights from the genome of the biotrophic fungal plant pathogen *Ustilago maydis*. Nature 444:97–101

Kis-Papo T, Weig AR, Riley R, Peršoh D, Salamov A, Sun H, Lipzen A, Wasser SP, Rambold G, Grigoriev I V, Nevo E (2014) Genomic adaptations of the halophilic Dead Sea filamentous fungus *Eurotium rubrum*. Nat Commun 5:3745

Kohler A, Kuo A, Nagy LG, Morin E, Barry KW, Buscot F, Canbäck B, Choi C, Cichocki N, Clum A, Colpaert J, Copeland A, Costa MD, Doré J, Floudas D, Gay G, Girlanda M, Henrissat B, Herrmann S, Hess J, Högberg N, Johansson T, Khouja H-R, LaButti K, Lahrmann U, Levasseur A, Lindquist EA, Lipzen A, Marmeisse R, Martino E, Murat C, Ngan CY, Nehls U, Plett JM, Pringle A, Ohm RA, Perotto S, Peter M, Riley R, Rineau F, Ruytinx J, Salamov A, Shah F, Sun H, Tarkka M, Tritt A, Veneault-Fourrey C, Zuccaro A, Mycorrhizal Genomics Initiative Consortium, Tunlid A, Grigoriev I V, Hibbett DS, Martin F (2015) Convergent losses of decay mechanisms and rapid turnover of symbiosis genes in mycorrhizal mutualists. Nat Genet 47:410–415

Konishi M, Hatada Y, Horiuchi J-I (2013) Draft genome sequence of the basidiomycetous yeast-like fungus Pseudozyma hubeiensis SY62, which produces an abundant amount of the biosurfactant mannosylerythritol lipids. Genome Announc 1:

Kourist R, Bracharz F, Lorenzen J, Kracht ON, Chovatia M, Daum C, Deshpande S, Lipzen A, Nolan M, Ohm RA, Grigoriev I V, Sun S, Heitman J, Brück T, Nowrousian M (2015) Genomics and transcriptomics analyses of the oil-accumulating Basidiomycete yeast *Trichosporon oleaginosus*: Insights into substrate utilization and alternative evolutionary trajectories of fungal mating systems. MBio 6:

Krizsán K, Almási É, Merényi Z, Sahu N, Virágh M, Kószó T, Mondo S, Kiss B, Bálint B, Kües U, Barry K, Cseklye J, Hegedüs B, Henrissat B, Johnson J, Lipzen A, Ohm RA, Nagy I, Pangilinan J, Yan J, Xiong Y, Grigoriev I V, Hibbett DS, Nagy LG (2019) Transcriptomic atlas of mushroom development reveals conserved genes behind complex multicellularity in fungi. Proc Natl Acad Sci U S A 116:7409–7418

Kusuya Y, Sakai K, Kamei K, Takahashi H, Yaguchi T (2016) Draft genome sequence of the pathogenic filamentous fungus *Aspergillus lentulus* IFM 54703 T. Genome Announc 4:

Kusuya Y, Takahashi-Nakaguchi A, Takahashi H, Yaguchi T (2015) Draft genome sequence of the pathogenic filamentous fungus *Aspergillus udagawae* strain IFM 46973 T. Genome Announc 3:

Kuuskeri J, Häkkinen M, Laine P, Smolander O-P, Tamene F, Miettinen S, Nousiainen P, Kemell M, Auvinen P, Lundell T (2016) Time-scale dynamics of proteome and transcriptome of the white-rot fungus *Phlebia radiata*: growth on spruce wood and decay effect on lignocellulose. Biotechnol Biofuels 9:192

Laurie JD, Ali S, Linning R, Mannhaupt G, Wong P, Güldener U, Münsterkötter M, Moore R, Kahmann R, Bakkeren G, Schirawski J (2012) Genome comparison of barley and maize smut fungi reveals targeted loss of RNA silencing components and species-specific presence of transposable elements. Plant Cell 24:1733–1745

Lenassi M, Gostinčar C, Jackman S, Turk M, Sadowski I, Nislow C, Jones S, Birol I, Cimerman NG, Plemenitaš A (2013) Whole genome duplication and enrichment of metal cation transporters revealed by de novo genome sequencing of extremely halotolerant black yeast Hortaea werneckii. PLoS One 8:e71328

Levasseur A, Lomascolo A, Chabrol O, Ruiz-Dueñas FJ, Boukhris-Uzan E, Piumi F, Kües U, Ram AFJ, Murat C, Haon M, Benoit I, Arfi Y, Chevret D, Drula E, Kwon MJ, Gouret P, Lesage-Meessen L, Lombard V, Mariette J, Noirot C, Park J, Patyshakuliyeva A, Sigoillot JC, Wiebenga A, Wösten HAB, Martin F, Coutinho PM, de Vries RP, Martìnez AT, Klopp C, Pontarotti P, Henrissat B, Record E (2014) The genome of the white-rot fungus *Pycnoporus cinnabarinus*: a basidiomycete model with a versatile arsenal for lignocellulosic biomass breakdown. BMC Genomics 15:486

Liers C, Bobeth C, Pecyna M, Ullrich R, Hofrichter M (2010) DyP-like peroxidases of the jelly fungus *Auricularia auricula-judae* oxidize nonphenolic lignin model compounds and high-redox potential dyes. Appl Microbiol Biotechnol 85:1869–1879

Linz JE, Wee J, Roze L V (2014) *Aspergillus parasiticus* SU-1 genome sequence, predicted chromosome structure, and comparative gene expression under aflatoxin-inducing conditions: Evidence that differential expression contributes to species phenotype. Eukaryot Cell 13:1113–1123

Liu Y, Wu Y, Zhang Y, Yang X, Yang E, Xu H, Yang Q, Chagan I, Cui X, Chen W, Yan J (2019) Lignin degradation potential and draft genome sequence of *Trametes trogii* S0301. Biotechnol Biofuels 12:256

Loftus BJ, Fung E, Roncaglia P, Rowley D, Amedeo P, Bruno D, Vamathevan J, Miranda M, Anderson IJ, Fraser JA, Allen JE, Bosdet IE, Brent MR, Chiu R, Doering TL, Donlin MJ, D'Souza CA, Fox DS, Grinberg V, Fu J, Fukushima M, Haas BJ, Huang JC, Janbon G, Jones SJM, Koo HL, Krzywinski MI, Kwon-Chung JK, Lengeler KB, Maiti R, Marra MA, Marra RE, Mathewson CA, Mitchell TG, Pertea M, Riggs FR, Salzberg SL, Schein JE, Shvartsbeyn A, Shin H, Shumway M, Specht CA, Suh BB, Tenney A, Utterback TR, Wickes BL, Wortman JR, Wye NH, Kronstad JW, Lodge JK, Heitman J, Davis RW, Fraser CM, Hyman RW (2005) The genome of the basidiomycetous yeast and human pathogen *Cryptococcus neoformans*. Science (80) 307:1321–1324

Lorenz S, Guenther M, Grumaz C, Rupp S, Zibek S, Sohn K (2014) Genome sequence of the basidiomycetous fungus *Pseudozyma aphidis* DSM70725, an efficient producer of biosurfactant mannosylerythritol lipids. Genome Announc 2:

Mardanov A V, Beletsky A V, Kadnikov V V, Ignatov AN, Ravin N V (2014) Draft genome sequence of *Sclerotinia borealis*, a psychrophilic plant pathogenic fungus. Genome Announc 2:

Martin F, Aerts A, Ahrén D, Brun A, Danchin EGJ, Duchaussoy F, Gibon J, Kohler A, Lindquist E, Pereda V, Salamov A, Shapiro HJ, Wuyts J, Blaudez D, Buée M, Brokstein P, Canbäck B, Cohen D, Courty PE, Coutinho PM, Delaruelle C, Detter JC, Deveau A, DiFazio S, Duplessis S, Fraissinet-Tachet L, Lucic E, Frey-Klett P, Fourrey C, Feussner I, Gay G, Grimwood J, Hoegger PJ, Jain P, Kilaru S, Labbé J, Lin YC, Legué V, Le Tacon F, Marmeisse R, Melayah D, Montanini B, Muratet M, Nehls U, Niculita-Hirzel H, Secq MPO-L, Peter M, Quesneville H, Rajashekar B, Reich M, Rouhier N, Schmutz J, Yin T, Chalot M, Henrissat B, Kües U, Lucas S, de Peer Y, Podila GK, Polle A, Pukkila PJ, Richardson PM, Rouzé P, Sanders IR, Stajich JE, Tunlid A, Tuskan G, Grigoriev I V (2008) The genome of *Laccaria bicolor* provides insights into mycorrhizal symbiosis. Nature 452:88–92

Mäkelä MR, Peng M, Granchi Z, Chin-A-Woeng T, Hegi R, van Pelt SI, Ahrendt S, Riley R, Hainaut M, Henrissat B, Grigoriev I V, de Vries RP, Hildén KS (2018) Draft genome sequence of the basidiomycete white-rot fungus *Phlebia centrifuga*. Genome Announc 6:

Miettinen O, Riley R, Barry K, Cullen D, de Vries RP, Hainaut M, Hatakka A, Henrissat B, Hildén K, Kuo R, LaButti K, Lipzen A, Mäkelä MR, Sandor L, Spatafora JW, Grigoriev I V, Hibbett DS (2016) Draft genome sequence of the basidiomycete white-rot fungus *Obba rivulosa* 3A-2. Genome Announc 4:

Miller ME, Zhang Y, Omidvar V, Sperschneider J, Schwessinger B, Raley C, Palmer JM, Garnica D, Upadhyaya N, Rathjen J, Taylor JM, Park RF, Dodds PN, Hirsch CD, Kianian SF, Figueroa M (2018) De Novo Assembly and Phasing of Dikaryotic Genomes from Two Isolates of Puccinia coronata f. sp. avenae, the Causal Agent of Oat Crown Rust. MBio 9:
Min B, Park H, Jang Y, Kim J-J, Kim KH, Pangilinan J, Lipzen A, Riley R, Grigoriev I V, Spatafora JW, Choi I-G (2015) Genome sequence of a white rot fungus *Schizopora paradoxa* KUC8140 for wood decay and mycoremediation. J Biotechnol 211:42–43

Miyauchi S, Rancon A, Drula E, Hage H, Chaduli D, Favel A, Grisel S, Henrissat B, Herpoël-Gimbert I, Ruiz-Dueñas FJ, Chevret D, Hainaut M, Lin J, Wang M, Pangilinan J, Lipzen A, Lesage-Meessen L, Navarro D, Riley R, Grigoriev I V, Zhou S, Raouche S, Rosso M-N (2018) Integrative visual omics of the white-rot fungus *Polyporus brumalis* exposes the biotechnological potential of its oxidative enzymes for delignifying raw plant biomass. Biotechnol Biofuels 11:

Mondego JMC, Carazzolle MF, Costa GGL, Formighieri EF, Parizzi LP, Rincones J, Cotomacci C, Carraro DM, Cunha AF, Carrer H, Vidal RO, Estrela RC, Garc\'\ia O, Thomazella DPT, de Oliveira B V, Pires AB, Rio MCS, Araújo MRR, de Moraes MH, Castro LAB, Gramacho KP, Gonçalves MS, Neto JPM, Neto AG, Barbosa L V, Guiltinan MJ, Bailey BA, Meinhardt LW, Cascardo JC, Pereira GAG (2008) A genome survey of Moniliophthora perniciosa gives new insights into Witches' Broom Disease of cacao. BMC Genomics 9:548
Mondo SJ, Dannebaum RO, Kuo RC, Louie KB, Bewick AJ, LaButti K, Haridas S, Kuo A, Salamov A, Ahrendt SR, Lau R, Bowen BP, Lipzen A, Sullivan W, Andreopoulos BB, Clum A, Lindquist E, Daum C, Northen TR, Kunde-Ramamoorthy G, Schmitz RJ, Gryganskyi A, Culley D, Magnuson J, James TY, O'Malley MA, Stajich JE, Spatafora JW, Visel A, Grigoriev I V (2017) Widespread adenine N6-methylation of active genes in fungi. Nat Genet 49:964–968

Moore GG, Mack BM, Beltz SB, Gilbert MK (2016) Draft genome sequence of an aflatoxigenic Aspergillus species, *A. bombycis*. Genome Biol Evol 8:3297–3300

Morales-Cruz A, Amrine KCH, Blanco-Ulate B, Lawrence DP, Travadon R, Rolshausen PE, Baumgartner K, Cantu D (2015) Distinctive expansion of gene families associated with plant cell wall degradation, secondary metabolism, and nutrient uptake in the genomes of grapevine trunk pathogens. BMC Genomics 16:469

Morin E, Kohler A, Baker AR, Foulongne-Oriol M, Lombard V, Nagy LG, Ohm RA, Patyshakuliyeva A, Brun A, Aerts AL, Bailey AM, Billette C, Coutinho PM, Deakin G, Doddapaneni H, Floudas D, Grimwood J, Hildén K, Kües U, Labutti KM, Lapidus A, Lindquist EA, Lucas SM, Murat C, Riley RW, Salamov AA, Schmutz J, Subramanian V, Wösten HAB, Xu J, Eastwood DC, Foster GD, Sonnenberg ASM, Cullen D, de Vries RP, Lundell T, Hibbett DS, Henrissat B, Burton KS, Kerrigan RW, Challen MP, Grigoriev I V, Martin F (2012) Genome sequence of the button mushroom *Agaricus bisporus* reveals mechanisms governing adaptation to a humic-rich ecological niche. Proc Natl Acad Sci U S A 109:17501–17506

Morita T, Koike H, Koyama Y, Hagiwara H, Ito E, Fukuoka T, Imura T, Machida M, Kitamoto D (2013) Genome sequence of the basidiomycetous yeast *Pseudozyma antarctica* T-34, a producer of the glycolipid biosurfactants mannosylerythritol lipids. Genome Announc 1:e0006413

Mujic AB, Kuo A, Tritt A, Lipzen A, Chen C, Johnson J, Sharma A, Barry K, Grigoriev I V, Spatafora JW (2017) Comparative genomics of the ectomycorrhizal sister species *Rhizopogon vinicolor* and *Rhizopogon vesiculosus* (Basidiomycota: Boletales) reveals a Divergence of the mating type B locus. G3 7:1775–1789

Nagy LG, Riley R, Tritt A, Adam C, Daum C, Floudas D, Sun H, Yadav JS, Pangilinan J, Larsson K-H, Matsuura K, Barry K, Labutti K, Kuo R, Ohm RA, Bhattacharya SS, Shirouzu T, Yoshinaga Y, Martin FM, Grigoriev I V, Hibbett DS (2016) Comparative genomics of early-diverging mushroom-forming fungi provides insights into the origins of lignocellulose decay capabilities. Mol Biol Evol 33:959–970

Nazareno ES, Li F, Smith M, Park RF, Kianian SF, Figueroa M (2018) *Puccinia coronata* f. sp. *avenae* : a threat to global oat production. Mol Plant Pathol 19:1047–1060

Nemri A, Saunders DGO, Anderson C, Upadhyaya NM, Win J, Lawrence GJ, Jones DA, Kamoun S, Ellis JG, Dodds PN (2014) The genome sequence and effector complement of the flax rust pathogen *Melampsora lini*. Front Plant Sci 5:98

Yew SM, Chan CL, Ngeow YF, Toh YF, Na SL, Lee KW, Hoh C-C, Yee W-Y, Ng KP, Kuan CS (2016) Insight into different environmental niches adaptation and allergenicity from the *Cladosporium sphaerospermum* genome, a common human allergy-eliciting Dothideomycetes. Sci Rep 6:27008

Nguyen HDT, McMullin DR, Ponomareva E, Riley R, Pomraning KR, Baker SE, Seifert KA (2016) Ochratoxin A production by *Penicillium thymicola*. Fungal Biol 120:1041–1049

Nielsen JC, Grijseels S, Prigent S, Ji B, Dainat J, Nielsen KF, Frisvad JC, Workman M, Nielsen J (2017) Global analysis of biosynthetic gene clusters reveals vast potential of secondary metabolite production in *Penicillium* species. Nat Microbiol 2:17044

Nowrousian M, Teichert I, Masloff S, Kück U (2012) Whole-genome sequencing of *Sordaria macrospora* mutants identifies developmental genes. G3 2:261–270

Ohm RA, de Jong JF, Lugones LG, Aerts A, Kothe E, Stajich JE, de Vries RP, Record E, Levasseur A, Baker SE, Bartholomew KA, Coutinho PM, Erdmann S, Fowler TJ, Gathman AC, Lombard V, Henrissat B, Knabe N, Kües U, Lilly WW, Lindquist E, Lucas S, Magnuson JK, Piumi F, Raudaskoski M, Salamov A, Schmutz J, Schwarze FWMR, vanKuyk PA, Horton JS, Grigoriev I V, Wösten HAB (2010) Genome sequence of the model mushroom *Schizophyllum commune*. Nat Biotechnol 28:957–963

de Wit PJGM, van der Burgt A, Ökmen B, Stergiopoulos I, Abd-Elsalam KA, Aerts AL, Bahkali AH, Beenen HG, Chettri P, Cox MP, Datema E, de Vries RP, Dhillon B, Ganley AR, Griffiths SA, Guo Y, Hamelin RC, Henrissat B, Kabir MS, Jashni MK, Kema G, Klaubauf S, Lapidus A, Levasseur A, Lindquist E, Mehrabi R, Ohm RA, Owen TJ, Salamov A, Schwelm A, Schijlen E, Sun H, van den Burg HA, van Ham RCHJ, Zhang S, Goodwin SB, Grigoriev I V, Collemare J, Bradshaw RE (2012) The genomes of the fungal plant pathogens *Cladosporium fulvum* and *Dothistroma septosporum* reveal adaptation to different hosts and lifestyles but also signatures of common ancestry. PLoS Genet 8:e1003088

Ohm RA, Feau N, Henrissat B, Schoch CL, Horwitz BA, Barry KW, Condon BJ, Copeland AC, Dhillon B, Glaser F, Hesse CN, Kosti I, LaButti K, Lindquist EA, Lucas S, Salamov AA, Bradshaw RE, Ciuffetti L, Hamelin RC, Kema GHJ, Lawrence C, Scott JA, Spatafora JW, Turgeon BG, de Wit PJGM, Zhong S, Goodwin SB, Grigoriev I V (2012) Diverse lifestyles and strategies of plant pathogenesis encoded in the genomes of eighteen *Dothideomycetes* fungi. PLoS Pathog 8:e1003037

Ohm RA, Riley R, Salamov A, Min B, Choi I-G, Grigoriev I V (2014) Genomics of wood-degrading fungi. Fungal Genet Biol 72:82–90

Oka T, Ekino K, Fukuda K, Nomura Y (2014) Draft genome sequence of the formaldehyde-resistant fungus *Byssochlamys spectabilis* No. 5 (Anamorph Paecilomyces variotii No. 5) ({NBRC109023}). Genome Announc 2:

Olson Å, Aerts A, Asiegbu F, Belbahri L, Bouzid O, Broberg A, Canbäck B, Coutinho PM, Cullen D, Dalman K, Deflorio G, van Diepen LTA, Dunand C, Duplessis S, Durling M, Gonthier P, Grimwood J, Fossdal CG, Hansson D, Henrissat B, Hietala A, Himmelstrand K, Hoffmeister D, Högberg N, James TY, Karlsson M, Kohler A, Kües U, Lee Y-H, Lin Y-C, Lind M, Lindquist E, Lombard V, Lucas S, Lundén K, Morin E, Murat C, Park J, Raffaello T, Rouzé P, Salamov A, Schmutz J, Solheim H, Ståhlberg J, Vélëz H, de Vries RP, Wiebenga A, Woodward S, Yakovlev I, Garbelotto M, Martin F, Grigoriev I V, Stenlid J (2012) Insight into trade-off between wood decay and parasitism from the genome of a fungal forest pathogen. New Phytol 194:1001–1013

Padamsee M, Kumar TKA, Riley R, Binder M, Boyd A, Calvo AM, Furukawa K, Hesse C, Hohmann S, James TY, LaButti K, Lapidus A, Lindquist E, Lucas S, Miller K, Shantappa S, Grigoriev I V, Hibbett DS, McLaughlin DJ, Spatafora JW, Aime MC (2012) The genome of the xerotolerant mold *Wallemia sebi* reveals adaptations to osmotic stress and suggests cryptic sexual reproduction. Fungal Genet Biol 49:217–226

Parent-Michaud M, Dufresne PJ, Fournier É, Martineau C, Moreira S, Perkins V, de Repentigny L, Dufresne SF (2019) Draft genome sequence of azole-resistant *Aspergillus thermomutatus* (*Neosartorya pseudofischeri*) strain HMR-AF-39, isolated from a human nasal septum abscess aspirate. Microbiol Resour Announc 8:

Park H, Min B, Jang Y, Kim J, Lipzen A, Sharma A, Andreopoulos B, Johnson J, Riley R, Spatafora JW, Henrissat B, Kim KH, Grigoriev I V, Kim J-J, Choi I-G (2019) Comprehensive genomic and transcriptomic analysis of polycyclic aromatic hydrocarbon degradation by a mycoremediation fungus, *Dentipellis* sp. KUC8613. Appl Microbiol Biotechnol 103:8145–8155

Pendleton AL, Smith KE, Feau N, Martin FM, Grigoriev I V, Hamelin R, Nelson CD, Burleigh JG, Davis JM (2014) Duplications and losses in gene families of rust pathogens highlight putative effectors. Front Plant Sci 5:299

Peng M, Dilokpimol A, Mäkelä MR, Hildén K, Bervoets S, Riley R, Grigoriev I V, Hainaut M, Henrissat B, de Vries RP, Granchi Z (2017) The draft genome sequence of the ascomycete fungus *Penicillium subrubescens* reveals a highly enriched content of plant biomass related CAZymes compared to related fungi. J Biotechnol 246:1–3

Perlin MH, Amselem J, Fontanillas E, Toh SS, Chen Z, Goldberg J, Duplessis S, Henrissat B, Young S, Zeng Q, Aguileta G, Petit E, Badouin H, Andrews J, Razeeq D, Gabaldón T, Quesneville H, Giraud T, Hood ME, Schultz DJ, Cuomo CA (2015) Sex and parasites: genomic and transcriptomic analysis of *Microbotryum lychnidis-dioicae*, the biotrophic and plant-castrating anther smut fungus. BMC Genomics 16:461

Peter M, Kohler A, Ohm RA, Kuo A, Krützmann J, Morin E, Arend M, Barry KW, Binder M, Choi C, Clum A, Copeland A, Grisel N, Haridas S, Kipfer T, Labutti K, Lindquist E, Lipzen A, Maire R, Meier B, Mihaltcheva S, Molinier V, Murat C, Pöggeler S, Quandt CA, Sperisen C, Tritt A, Tisserant E, Crous PW, Henrissat B, Nehls U, Egli S, Spatafora JW, Grigoriev I V., Martin FM (2016) Ectomycorrhizal ecology is imprinted in the genome of the dominant symbiotic fungus *Cenococcum geophilum*. Nat Commun 7:12662. https://doi.org/10.1038/ncomms12662

Riley R, Salamov AA, Brown DW, Nagy LG, Floudas D, Held BW, Levasseur A, Lombard V, Morin E, Otillar R, Lindquist EA, Sun H, LaButti KM, Schmutz J, Jabbour D, Luo H, Baker SE, Pisabarro AG, Walton JD, Blanchette RA, Henrissat B, Martin F, Cullen D, Hibbett DS, Grigoriev I V (2014) Extensive sampling of basidiomycete genomes demonstrates inadequacy of the white-rot/brown-rot paradigm for wood decay fungi. Proc Natl Acad Sci USA 111:9923–9928

Ruiz-Dueñas FJ, Lundell T, Floudas D, Nagy LG, Barrasa JM, Hibbett DS, Martìnez AT (2013) Lignin-degrading peroxidases in Polyporales: an evolutionary survey based on 10 sequenced genomes. Mycologia 105:1428–1444

Schirawski J, Mannhaupt G, Münch K, Brefort T, Schipper K, Doehlemann G, Di Stasio M, Rössel N, Mendoza-Mendoza A, Pester D, Müller O, Winterberg B, Meyer E, Ghareeb H, Wollenberg T, Münsterkötter M, Wong P, Walter M, Stukenbrock E, Güldener U, Kahmann R (2010) Pathogenicity determinants in smut fungi revealed by genome comparison. Science 330:1546–1548

Schwessinger B, Sperschneider J, Cuddy WS, Garnica DP, Miller ME, Taylor JM, Dodds PN, Figueroa M, Park RF, Rathjen JP (2018) A near-complete haplotype-phased genome of the dikaryotic wheat stripe rust fungus *Puccinia striiformis* f. sp. *tritici* reveals high interhaplotype diversity. MBio 9(1): e02275-17.

Sipos G, Prasanna AN, Walter MC, O’Connor E, Bálint B, Krizsán K, Kiss B, Hess J, Varga T, Slot J, Riley R, Bóka B, Rigling D, Barry K, Lee J, Mihaltcheva S, LaButti K, Lipzen A, Waldron R, Moloney NM, Sperisen C, Kredics L, Vágvölgyi C, Patrignani A, Fitzpatrick D, Nagy I, Doyle S, Anderson JB, Grigoriev I V, Güldener U, Münsterkötter M, Nagy LG (2017) Genome expansion and lineage-specific genetic innovations in the forest pathogenic fungi *Armillaria*. Nat Ecol Evol 1:1931–1941

Stajich JE, Wilke SK, Ahrén D, Au CH, Birren BW, Borodovsky M, Burns C, Canbäck B, Casselton LA, Cheng CK, Deng J, Dietrich FS, Fargo DC, Farman ML, Gathman AC, Goldberg J, Guigó R, Hoegger PJ, Hooker JB, Huggins A, James TY, Kamada T, Kilaru S, Kodira C, Kües U, Kupfer D, Kwan HS, Lomsadze A, Li W, Lilly WW, Ma L-J, Mackey AJ, Manning G, Martin F, Muraguchi H, Natvig DO, Palmerini H, Ramesh MA, Rehmeyer CJ, Roe BA, Shenoy N, Stanke M, Ter-Hovhannisyan V, Tunlid A, Velagapudi R, Vision TJ, Zeng Q, Zolan ME, Pukkila PJ (2010) Insights into evolution of multicellular fungi from the assembled chromosomes of the mushroom *Coprinopsis cinerea* (*Coprinus cinereus*). Proc Natl Acad Sci U S A 107:11889–11894

Stukenbrock EH, Jørgensen FG, Zala M, Hansen TT, McDonald BA, Schierup MH (2010) Whole-genome and chromosome evolution associated with host adaptation and speciation of the wheat pathogen *Mycosphaerella graminicola*. PLoS Genet 6:e1001189

Suzuki H, MacDonald J, Syed K, Salamov A, Hori C, Aerts A, Henrissat B, Wiebenga A, VanKuyk PA, Barry K, Lindquist E, LaButti K, Lapidus A, Lucas S, Coutinho P, Gong Y, Samejima M, Mahadevan R, Abou-Zaid M, de Vries RP, Igarashi K, Yadav JS, Grigoriev I V, Master ER (2012) Comparative genomics of the white-rot fungi, *Phanerochaete carnosa* and *P. chrysosporium*, to elucidate the genetic basis of the distinct wood types they colonize. BMC Genomics 13:444

Tang JD, Perkins AD, Sonstegard TS, Schroeder SG, Burgess SC, Diehl SV (2012) Short-read sequencing for genomic analysis of the brown rot fungus *Fibroporia radiculosa*. Appl Environ Microbiol 78:2272–2281

Teixeira MM, Moreno LF, Stielow BJ, Muszewska A, Hainaut M, Gonzaga L, Abouelleil A, Patané JSL, Priest M, Souza R, Young S, Ferreira KS, Zeng Q, da Cunha MML, Gladki A, Barker B, Vicente VA, de Souza EM, Almeida S, Henrissat B, Vasconcelos ATR, Deng S, Voglmayr H, Moussa TAA, Gorbushina A, Felipe MSS, Cuomo CA, de Hoog GS (2017) Phylogeny and taxonomy of the scab and spot anthracnose fungus Elsinoë (Myriangiales, Dothideomycetes). Stud Mycol 86:1–28

Thines M, Telle S, Choi Y-J, Tan YP, Shivas RG (2015) *Baobabopsis*, a new genus of graminicolous downy mildews from tropical Australia, with an updated key to the genera of downy mildews. IMA Fungus 6:483–491

Toome M, Kuo A, Henrissat B, Lipzen A, Tritt A, Yoshinaga Y, Zane M, Barry K, Grigoriev I V, Spatafora JW, Aime MC (2014) Draft genome sequence of a rare smut relative, *Tilletiaria anomala* UBC 951. Genome Announc 2(3): e00539-14.

Toome M, Ohm RA, Riley RW, James TY, Lazarus KL, Henrissat B, Albu S, Boyd A, Chow J, Clum A, Heller G, Lipzen A, Nolan M, Sandor L, Zvenigorodsky N, Grigoriev I V, Spatafora JW, Aime MC (2014) Genome sequencing provides insight into the reproductive biology, nutritional mode and ploidy of the fern pathogen *Mixia osmundae*. New Phytol 202:554–564

Varga T, Krizsán K, Földi C, Dima B, Sánchez-Garcia M, Sánchez-Ramirez S, Szöll H osi GJ, Szarkándi JG, Papp V, Albert L, Andreopoulos W, Angelini C, Antonìn V, Barry KW, Bougher NL, Buchanan P, Buyck B, Bense V, Catcheside P, Chovatia M, Cooper J, Dämon W, Desjardin D, Finy P, Geml J, Haridas S, Hughes K, Justo A, Karasiński D,

Kautmanova I, Kiss B, Kocsubé S, Kotiranta H, LaButti KM, Lechner BE, Liimatainen K, Lipzen A, Lukács Z, Mihaltcheva S, Morgado LN, Niskanen T, Noordeloos ME, Ohm RA, Ortiz-Santana B, Ovrebo C, Rácz N, Riley R, Savchenko A, Shiryaev A, Soop K, Spirin V, Szebenyi C, Tomšovský M, Tulloss RE, Uehling J, Grigoriev I V, Vágvölgyi C, Papp T, Martin FM, Miettinen O, Hibbett DS, Nagy LG (2019) Megaphylogeny resolves global patterns of mushroom evolution. Nat Ecol Evol 3:668–678

Verma S, Gazara RK, Nizam S, Parween S, Chattopadhyay D, Verma PK (2016) Draft genome sequencing and secretome analysis of fungal phytopathogen *Ascochyta rabiei* provides insight into the necrotrophic effector repertoire. Sci Rep 6:24638

Walker AK, Frasz SL, Seifert KA, Miller JD, Mondo SJ, LaButti K, Lipzen A, Dockter RB, Kennedy MC, Grigoriev I V, Spatafora JW (2016) Full genome of *Phialocephala scopiformis* DAOMC 229536, a fungal endophyte of spruce producing the potent anti-insectan compound rugulosin. Genome Announc 4:

Wang Y-Y, Liu B, Zhang X-Y, Zhou Q-M, Zhang T, Li H, Yu Y-F, Zhang X-L, Hao X-Y, Wang M, Wang L, Wei J-C (2014) Genome characteristics reveal the impact of lichenization on lichen-forming fungus *Endocarpon pusillum* Hedwig (Verrucariales, Ascomycota). BMC Genomics 15:34

Wawrzyn GT, Quin MB, Choudhary S, López-Gallego F, Schmidt-Dannert C (2012) Draft genome of *Omphalotus olearius* provides a predictive framework for sesquiterpenoid natural product biosynthesis in Basidiomycota. Chem Biol 19:772–783

Wibberg D, Jelonek L, Rupp O, Hennig M, Eikmeyer F, Goesmann A, Hartmann A, Borriss R, Grosch R, Pühler A, Schlüter A (2013) Establishment and interpretation of the genome sequence of the phytopathogenic fungus *Rhizoctonia solani* {AG1-IB} isolate 7/3/14. J Biotechnol 167:142–155

Wingfield BD, Barnes I, de Beer Z, De Vos L, Duong TA, Kanzi AM, Naidoo K, Nguyen HDT, Santana QC, Sayari M, Seifert KA, Steenkamp ET, Trollip C, van der Merwe NA, van der Nest MA, Markus Wilken P, Wingfield MJ (2015) {IMA} {Genome-F} 5: Draft genome sequences of *Ceratocystis eucalypticola, Chrysoporthe cubensis, C. deuterocubensis, Davidsoniella virescens, Fusarium temperatum,Graphilbum fragrans, Penicillium nordicum,* and *Thielaviopsis musarum*. IMA Fungus 6:493–506

Xu J, Saunders CW, Hu P, Grant RA, Boekhout T, Kuramae EE, Kronstad JW, Deangelis YM, Reeder NL, Johnstone KR, Leland M, Fieno AM, Begley WM, Sun Y, Lacey MP, Chaudhary T, Keough T, Chu L, Sears R, Yuan B, Dawson Jr TL (2007) Dandruff-associated *Malassezia* genomes reveal convergent and divergent virulence traits shared with plant and human fungal pathogens. Proc Natl Acad Sci U S A 104:18730–18735

Yang RY, Li HT, Zhu H, Zhou GP, Wang M, Wang L (2012) Genome sequence of the Trichosporon asahii environmental strain {CBS} 8904. Eukaryot Cell 11:1586–1587

Zajc J, Liu Y, Dai W, Yang Z, Hu J, Gostinčar C, Gunde-Cimerman N (2013) Genome and transcriptome sequencing of the halophilic fungus *Wallemia ichthyophaga*: haloadaptations present and absent. BMC Genomics 14:617

Zeiner CA, Purvine SO, Zink EM, Paša-Tolić L, Chaput DL, Haridas S, Wu S, LaButti K, Grigoriev I V, Henrissat B, Santelli CM, Hansel CM (2016) Comparative analysis of secretome profiles of manganese(II)-oxidizing Ascomycete fungi. PLoS One 11:e0157844

Zuccaro A, Lahrmann U, Güldener U, Langen G, Pfiffi S, Biedenkopf D, Wong P, Samans B, Grimm C, Basiewicz M, Murat C, Martin F, Kogel K-H (2011) Endophytic life strategies decoded by genome and transcriptome analyses of the mutualistic root symbiont *Piriformospora indica.* PLoS Pathog 7:e1002290
